# An Isochore Framework Underlies Chromatin Architecture

**DOI:** 10.1371/journal.pone.0168023

**Published:** 2017-01-06

**Authors:** Kamel Jabbari, Giorgio Bernardi

**Affiliations:** 1 Max Planck Institute for Biology of Ageing, Joseph-Stelzmann-Straße 9B, Köln, Germany; 2 Science Department, Roma Tre University, Viale Marconi, Rome, Italy, and Stazione Zoologica Anton Dohrn, Villa Comunale, Naples, Italy; Fralin Life Science Institute, Virginia Tech, UNITED STATES

## Abstract

A recent investigation showed the existence of correlations between the architectural features of mammalian interphase chromosomes and the compositional properties of isochores. This result prompted us to compare maps of the Topologically Associating Domains (TADs) and of the Lamina Associated Domains (LADs) with the corresponding isochore maps of mouse and human chromosomes. This approach revealed that: 1) TADs and LADs correspond to isochores, *i*.*e*., isochores are the genomic units that underlie chromatin domains; 2) the conservation of TADs and LADs in mammalian genomes is explained by the evolutionary conservation of isochores; 3) chromatin domains corresponding to GC-poor isochores (*e*.*g*., LADs) show not only self-interactions but also intrachromosomal interactions with other domains also corresponding to GC-poor isochores even if located far away; in contrast, chromatin domains corresponding to GC-rich isochores (*e*.*g*., TADs) show more localized chromosomal interactions, many of which are inter-chromosomal. In conclusion, this investigation establishes a link between DNA sequences and chromatin architecture, explains the evolutionary conservation of TADs and LADs and provides new information on the spatial distribution of GC-poor/gene-poor and GC-rich/gene-rich chromosomal regions in the interphase nucleus.

## Introduction

By assessing the probability of physical proximity between pairs of loci, the chromosome conformation capture (3C) approach [1] and its further developments, such as Hi-C [2] and *in situ* Hi-C [3], have revolutionized our understanding of mammalian chromatin architecture. The three-dimensional structure of chromatin is now seen as consisting of topologically associating domains (TADs), a system of chromatin loops and boundaries 0.2–2 Mb in size [4,5], the majority of which can be resolved into contact domains, 185 Kb in median size [3]. Another approach, DamID technology, has shown that lamina-associated domains (LADs) have a median size of 500 Kb are GC-poor and are scattered over all chromosomes [6,7]. It is also well established that TADs and contact domains are largely conserved across different mouse and human cell types [3,4,8]. Moreover: 1) the regulation of gene expression is affected by topological changes such as boundary deletions and domain perturbations (see [9–16] for recent reviews); and 2) senescence is accompanied by alterations in LAD/lamina interactions [17]. However, the formation mechanism of topological domains is not yet understood, in spite of the recent advances in the field of chromatin architecture and of the interesting models proposed so far ([2, 3, 8, 18–20]; see [11] for a critical review).

The investigations just mentioned (and many others not cited here) were mainly focused on the connections between interphase chromatin structure and gene expression/regulation and replication on the one hand and on chromatin/lamina interactions on the other. We were aware for a long time, however, of the existence of correlations between the base composition (and related properties) of isochores and the structural features of chromosomes (see Table A-B in S1 File and [21]). Moreover, a recent analysis revealed that correlations exist between the two main compositional compartments of isochores (the large GC-poor/gene-poor genome desert and the small GC-rich/gene-rich genome core) and the architecture of chromatin (see Table B in S1 File and [21]). These correlations encouraged us to investigate in detail the genomic basis of chromatin architecture. This was done by comparing maps of TADs and LADs with the corresponding maps of isochores from mouse and human chromosomes and by looking for an explanation for the evolutionary conservation of TADs.

## Materials and Methods

### Data sets

Ensembl annotation [22] was used to collect a non-redundant set of human genes with HGNC (HUGO Gene Nomenclature Committee) protein coding entries (19,202 genes). To study the correlation between isochores and chromatin structures in mammalian cells, we used TAD coordinates from the genome-wide chromatin interaction frequencies (Hi-C experiments) performed on human embryonic stem cells ES and human fibroblasts IMR90 [4]. UCSC batch coordinate conversion (liftOver) was used to convert isochore coordinates (from [23]) and genome annotation files to hg19 release. Isochore maps were generated using "draw-chromosome-gc.pl" [24]. *In situ* HiC maps of human IMR90 cells were visualized at different resolution using Juicebox [3], a software for plotting data from proximity mapping experiments, HiC maps are stored and can be visualized at different resolutions as well as with the normalization algorithm of choice. Here we visualized interaction maps using the normalization of [3], which is based on a matrix-balancing algorithm. The same approach was used to examine mouse TAD maps from [8].

### Syntenic regions

Orthologous gene pairs from three mammalian species (human, mouse and dog) were extracted from Ensembl database. Orthologous pairs and their annotation were downloaded via the customizable BioMart data-mining tool. Syntenic blocks were restricted to those intervals embedding at least 3 orthologs on the same chromosome arm. Orthologous gene sets with conserved order were grouped into genomic blocks of synteny. The 1,348 human/mouse synteny blocks so obtained have an average size of 813,739 bp (min = 58,490 bp, max = 10,547,667 bp). The 1,358 human/dog synteny blocks have an average size of 785,204 bp (min = 60,410 bp, max = 6,020,611 bp). Using the intersection between the syntenic blocks and the human isochores coordinates, a set of orthologous isochores was obtained.

### Statistical analysis of the isochore/TADs correspondence

We asked whether the isochores sets overlap with the TADs sets more than expected by chance alone. To answer and assess quantitatively these trends we performed an association analysis of genomic regions based on permutation tests using the R/Bioconductor package regioneR [25]. The results of the permutation outcomes were subsequently evaluated with the *p*-values and the z-scores of the test. Fig S1A and Fig S1B in S2 File show that the test was very significant (*p*-values < 0.001), reflecting the non-random association between the two sets of genomic intervals (isochores and TADs). We have also analyzed the association between TADs from ES cells and IMR90 fibroblasts and used it as a validation test and reference for the strength of the association between isochores and TADs (Fig S1C in S2 File). The concordance between TADs from different cell types was found to be almost as strong as the concordance between isochores and TADs.

When performing an association analysis, it is possible to detect associations that, while statistically significant, are not specific. With the "localZscore" function [25] one can verify if the association between TADs and isochores is specifically linked to the common boundaries positions of the analyzed regions sets. The main function to perform a permutation test with *regioneR* (an R package) is “permTest”, a function taking a region set (RS), a randomization function and an evaluation function and returning a permTestResults object. The function performs the whole permutation test analysis, evaluating the original RS, creating a number of randomizations and evaluating them and finally computing the p-value and z-score. Such a z-score can be calculated several times by moving the RS and produces z-scores.

Basically, the RS is shifted a number of bases to 5' and 3' from its original position and the evaluation function is computed for every shifted RS. Plotting the evaluations over the shifted positions, we can appreciate how the value of the z-score changes when moving the interval: a peak at the center indicates that the association is highly dependent on the genomic coordinates while a flat profile will indicate that the association is regional (http://bioconductor.org/packages/release/bioc/vignettes/regioneR/inst/doc/regioneR.pdf).

Fig S2A in S2 File shows how as soon as we displace the regions away from their original positions, the z-score drops considerably in a similar fashion to the TADs-ES vs TADs-IMR90 z-score drops, where a sharp peak, indicative of a better fit among intervals, is observed (Fig S2B in S2 File). These results provide strong evidence for the proposal that TADs and isochores specifically match each other; discrepancies are likely due to resolution differences and boundary definitions between the compared sets and cell specific chromatin folding state, which is stressed by the observation that 65% to 72% of TADs boundaries are common between hESC cells and IMR90 fibroblast [4].

### Isochores/TADs/LADs alignments

Juicebox [3] offers the possibility of navigation through Hi-C data archive from studies published between 2009 and 2015; we explored this data and focused on high quality and resolution maps [3,8], namely, mouse CH12-LX B-lymphoblasts from HC088-HIC102 (combined *in situ* data) experiments, IMR90 cells from experiment HIC050-HIC056 (combined *in situ* data) [3] and mouse interaction maps from [22]. Manual alignment of isochore maps and TADs was performed and the recently published human LAD maps [7] were juxtaposed to the isochores map by coordinate adjustments. Nuclear Lamina (NL) contact frequencies (CF) were defined as the proportion of cells in which this segment contacted the NL [7].

Although TADs comprise by definition all the topologically associating domains, in the context of this article (and whenever appropriate) we refer to TADs as the topologically associating domains that do not overlap with LADs, the lamina associated domains.

## Results

### TADs, LADs and isochores from human and mouse chromosomes

**Fig 1:**
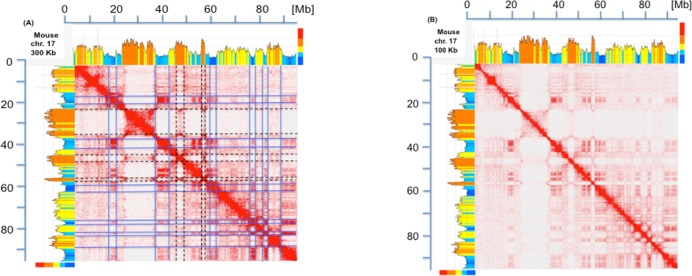
Heat map of chromatin interactions and isochores map of mouse chromosome 17. (A). The heat map of chromatin interactions in mouse chromosome 17 (from [8]) is compared with the corresponding compositional profile (drawn from mm 10 genome assembly using a sliding window of 300 Kb and the program of [24]). Isochore families L1 to H3, characterized by increasing GC levels are defined according to the “fixed” boundaries between isochore families (see Table A in S1 File) and are represented in different colors, deep blue, light blue, yellow, orange and red, respectively; the multicolored vertical bars on the top right indicate GC levels that correspond to the compositional boundaries among isochore families. The self-interactions along the diagonal, as well as the interactions along the two major axes, correspond to short isochore blocks or to individual isochores, as stressed by lines through the coinciding boundaries of TADs (blue and broken black lines correspond to GC-poor and GC-rich isochores or isochore blocks, respectively; not all lines were drawn to avoid readability problems). Interactions corresponding to GC-poor regions are also seen in domains corresponding to other GC-poor regions even when located far away on the chromosomes, the intensity of such interactions decreasing, however, with distance. In the case of interactions corresponding to GC-rich isochores, much weaker signals are present outside the diagonal, an indication of more localized interactions. Note that mouse chromosome 17 is an acrocentric chromosome and that the centromeric sequences correspond to the region near the origin of the Megabase (Mb) scale. Unless otherwise stated, all the interaction maps presented in this article are shown at a 250 Kb resolution and isochores are visualized using a 300 Kb sliding window across chromosomes. (B) The heat map of mouse chromosome 17 is compared at a resolution of 100 Kb with the corresponding isochore profile. Interactions along the diagonal are weaker because split into smaller domains, that reveal fine details. For example, the largest GC-rich region on the chromosome (around 30 Mb) is now resolved into several domains that present a finer correspondence with isochores.

The isochores/TADs comparison of mouse chromosomes indicated a good degree of match between the boundaries of TADs and the boundaries of the corresponding isochores or short isochore blocks, as outlined in Fig 1A for chromosome 17. In the case of GC-poor isochores, the correspondence not only concerned the self-interactions and the short-range interaction frequencies on the diagonal of the contact frequency matrix, but also the long-range ones along the two major axes. The latter interactions were found to concern domains that correspond to other, even faraway, GC-poor isochores. In contrast, TADs corresponding to GC-rich isochores showed strong interactions only with nearby TADs also corresponding to GC-rich isochores. At a higher resolution (100 Kb *vs*. 300 Kb), boundaries corresponding to isochore blocks (such as the rightmost one) appeared to be often resolved into boundaries corresponding to individual isochores (see Fig 1B).

**Fig 2:**
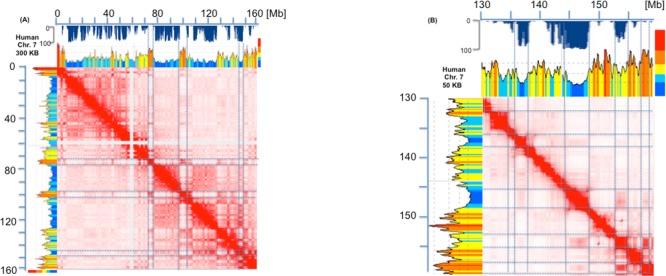
Heat map of chromatin interactions and isochores map of human chromosome 7. (A) The heat map of chromatin interactions of human chromosome 7 (from [3]) and the corresponding LAD map (from [7]; blue inverted profile under the Megabase axis) are compared with the corresponding compositional profile (using the matched b37 assembly of [3], and the program of [24]). The results are comparable with those of Fig 1A, except that the centromeric repeats (that have been sequenced in this chromosome) do not interact with any other sequence and are responsible for the two orthogonal blank stripes on the heat map. Blue lines correspond to TADs, LADs and inter-LADs. (B). The heat map of chromatin interactions over ~30 Mb of human chromosome 7 is analyzed at a resolution of 50 Kb. In this case a finer correspondence of isochore boundaries with LAD and TAD boundaries can be observed.

Results for human chromosomes (see Fig 2A and 2B for chromosome 7 at resolutions of 300 Kb and 50 Kb, respectively) were similar to those of Fig 1 (see the rightmost block of Fig 2B) in spite of the greater compositional complexity of the human genome (in which isochores extend further on both GC-poor and GC-rich sides and cover a ~25% GC range;[21]).

In this case, LAD maps were also available [7] and could be compared with isochore maps. This revealed a very good match between LADs and GC-poor isochores (as expected from previous work; 6, 7) and no match between LADs and GC-rich isochores. The findings of Figs 1 and 2 (that were extended to all mouse and human chromosomes; see a later section) indicate that TADs and LADs appear to correspond to individual isochores. Indeed, the cases in which TAD boundaries correspond to short isochore blocks seem to be essentially due to the structural complexity of a number of TADs, to the comparison of TADs from a given source with a genomic sequence from a different source (see legend of Fig 2A), and also to some oversegmentation of isochores due to the use of “fixed” boundaries” for the definition of isochore families (see Table A in S1 File). The correspondence of TADs and LADs with isochores is also supported by the independent evidence that isochores are replication units, characterized by all early or all late replicons in GC-rich and GC-poor isochores, respectively [26], and that TADs are stable units of replication timing [27].

**Fig 3:**
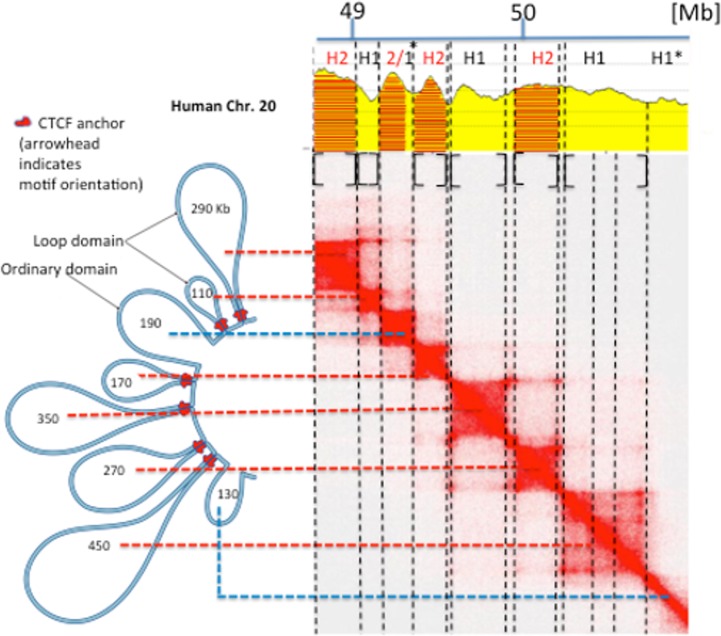
Chromatin loops and isochores from a 2.1 Mb region of human chromosome 20. The chromatin loops from a 2.1 Mb region of human chromosome 20 (Fig 6F from [3]) have been aligned with the corresponding heat map which was used to segment the corresponding DNA sequence into isochores. In this Figure the “extended” isochore ranges of Table A in S1 File were used to assign isochores to families, in order to take care of some minimal trespassings of the 46% GC upper threshold of H1 isochores. Asterisks indicate anomalies in the isochores/domains correspondence (see Text).

At a higher resolution, Fig 3 shows as a case study a comparison of loops and isochores (that can be identified with TADs) from a 2.1 Mb segment of human chromosome 20 (used as a paradigm in [3]) along with the corresponding contact matrix (also from [3]). Red and blue horizontal broken lines connect “loop domains” (anchored by CTCF, the CCCTC-binding factor) and “ordinary domains” (not anchored by CTCF), respectively, with the corresponding squares in the heat map. Thin vertical lines along the borders of the squares (double lines corresponding to intervals between loops whenever large enough) were used to segment the corresponding compositional profile of DNA. This approach was shown to segment DNA into a series of alternating H1 and H2 isochores and to establish a correspondence between chromatin loops and isochores (which is stressed by the square brackets under the compositional profile).

It should be noted 1) that the only exceptions to the alternating isochore rule concerns the two “ordinary domains”: the 190 Kb domain encompasses a H2/H1 sequence and the 130 Kb domain is formed by a H1 isochore which follows another H1 isochore; 2) that the largest 450 Kb loop consists of three sub-domains that are clearly visible on the heat map; and 3) that the 2.1 Mb region does not present any LAD signal, as expected from its GC-richness.

### Additional observations on isochores, TADs and LADs

A number of other observations are relevant: 1) the density of CTCF binding sites is decreasing in isochores from families of increasingly lower GC and especially low in family L1 (Fig S3 in S2 File); 2) normalized (mapping quality MAPQ> = 30) inter-chromosomal interactions (from [3]) have a very strong preference for GC-rich regions (see Fig 4). The analysis of inter-chromosomal interactions using pairwise interaction from CH12-LX_interchromosomal_contact_matrix (available for 20 human chromosomes) showed that for the most frequent 100 interactions (38,000 interactions, for all chromosome pairs at a 250 kb resolution), ~75% of mapped GC-rich isochores (H1+H2+H3) are involved in inter-chromosomal interactions, compared to 25% for GC-poor (L1+L2) isochores; 3) a comparison of isochore profiles with the sub-compartments of contact domains (from[3]) shows that, in general, A1 sub-compartments correspond to H2/H3 isochores (sometimes including flanking isochores from the H1 and even from the L2 family), A2 sub-compartments to H1 and L2 isochores, B1-B3 sub-compartments to L2 and L1 isochores (see Fig S4 in S2 File and its legend for further comments); needless to say, these results provide a more detailed picture (in the direction of isochore families) of the previously noted general correspondence [21] between (i) the open A and closed B chromatin compartments [2] and (ii) the genome core and the genome desert; 4) Figs S5-S23 and Figs S24-S45 in S2 File display the correspondence of isochores with TADs (and LADs) along all mouse and human chromosomes, respectively, so demonstrating the generality of the results of Figs 1 and 2.

**Fig 4:**
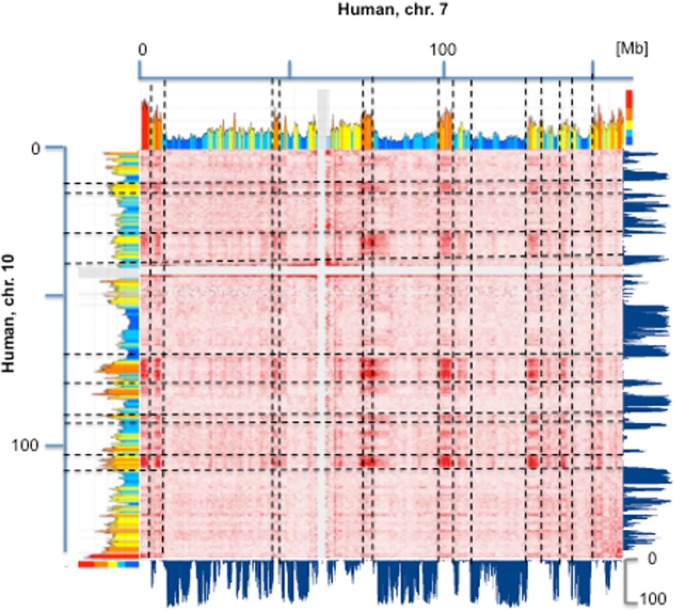
Inter-chromosomal interactions and isochores. The heat maps of chromatin interactions of human chromosomes 7 and 10 (from [3]) are compared with the corresponding compositional and LAD profiles (from [7]). Interactions appear to correspond to GC-rich isochores and inter-LADS and to be widely spread over the two chromosomes. Lines are used to guide the visual inspection of these features.

A detailed statistical analysis of isochores and TADs (from [4]) based on permutation tests [25] showed a non-random associations with a *p*-value <0.001; moreover, it was demonstrated that the association between isochores and TADs was specifically linked to common boundary positions (see Fig S1 and Fig S2 in S2 File). Using the same statistics as in TADs (*Permutation Test*), the overlapping between LADs and isochores is highly significant (p = 0.00093).

### Evolutionary conservations of TADs and isochores

**Fig 5:**
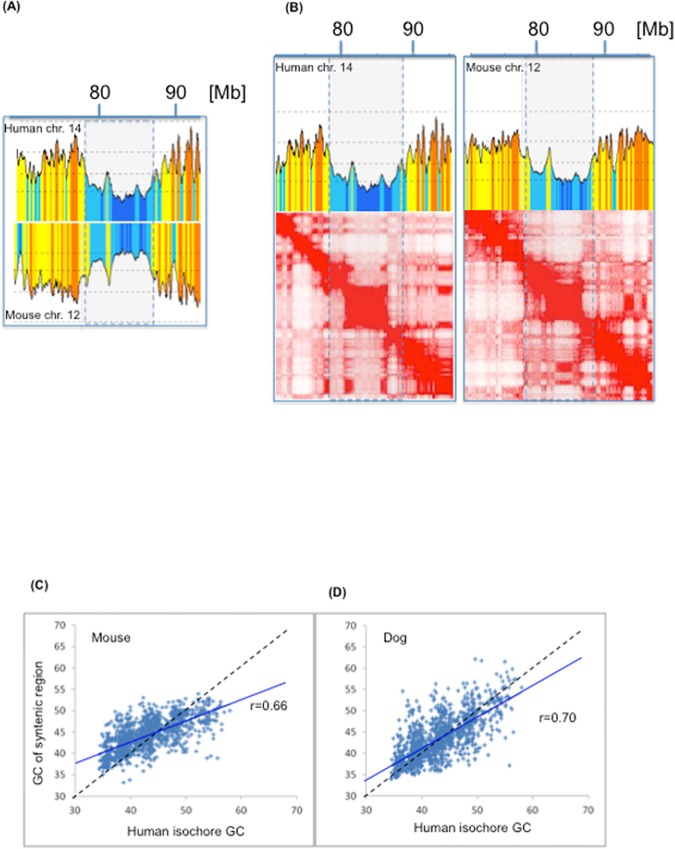
Evolutionary conservation of TADs and isochores. (A) The compositional profiles of two syntenic regions located on human chromosome 14 and mouse chromosome 12 (from [3, 8] respectively) are almost mirror images of each other, showing the compositional conservation of syntenic regions in evolution. (B) A comparison of compositional profiles and heat maps (from [3]) of the two syntenic regions from panel A. (C and D) GC levels of 1,348 and 1,358 syntenic regions from mouse (left) and dog (right) are plotted against the GC levels of human isochores.

As far as the evolutionary conservation of TADs is concerned, we examined the possible connections between the conservation of isochores [28, 29] and the conservation of chromatin domains [3, 4, 8] in syntenic loci. Fig 5 compares two syntenic regions of human chromosome 14 and mouse chromosome 12 and shows that the isochore profiles are almost mirror images of each other (Fig 5A) and that the regions correspond to similar heat maps of chromatin interactions (Fig 5B). Fig 5C and 5D show the correlations between GC levels of human isochores and the GC levels of 1,348 and 1,358 syntenic regions from mouse and dog, respectively. In both plots, highly significant correlations were found across the whole GC spectrum. This is an indication that GC-poor isochores (many of which correspond to LADs) are also conserved in evolution, in agreement with the evolutionary conservation of isochore families [30]. This provides an additional point in favor of the correspondence of isochores with TADs and LADs, because the conservation of isochores accounts for the conservation of chromatin architecture.

## Conclusions

The main conclusions of the present investigations are presented below.

1) The long-distance interactions of GC-poor domains mainly concerning LADs, on the one hand, and the “local” and interchromosomal interactions of GC-rich domains, on the other hand, provide a view of chromosomes in the interphase nucleus which is in line with results obtained using different approaches. Indeed, previous results indicated that GC-rich, early replicating, transcriptionally active chromatin regions are located in the nuclear interior [31] and that the gene-rich regions display a much more spread-out conformation [32, 33]. We understand now that this facilitates interchromosomal interactions that are not possible in the case of the gene-poor regions located at the nuclear periphery. In contrast, LADs (that correspond to GC-poor isochores) exhibit long range interactions that are, however, linked to the same chromosomes, a strong indication that such LADs may share a remarkable degree of contiguity on the lamina.

2) The isochore/loop correspondence implies the existence of different GC levels and different frequencies of di- and tri-nucleotides [26], in different TADs and LADs (as shown in Fig S3 and in Figs S5-S45 in S2 File. In other words, while the genome is a compositional mosaic of isochores [34, 35], interphase chromatin is a mosaic of different TADs and LADs. In turn, this is in agreement with different structural/functional properties of TADs (Table B-C in S1 File), whereas in the case of LADs, L1 isochores possibly correspond to the “stable contacts”, L2 isochores to the “variable contacts” of [7] (see also the legend of Figs S24-S45 in S2 File).

3) The key finding of these investigations, namely the correspondence of TADs and LADs with isochores, indicates that isochores should be visualized as the framework of chromatin architecture. In fact, the present results lead to a new paradigm of chromatin architecture which was obtained by taking into consideration its DNA backbone, the isochores, “a fundamental level of genome organization”[36], and by showing that isochores are the genomic units that underlie TADs and LADs. This establishes a connection between DNA sequences and chromatin architecture and opens the way to studying changes in chromatin architecture at the sequence level.

## Supporting Information

S1 File Table A, Isochore families in the human genome, Table B, Structural and functional properties of the genome core *vs*. the genome desert. Table C, Isochores & interphase chromatin.
(PDF)

S2 File Fig S1. Association analysis of genomic regions based on permutation test: (A) TADs from human IMR90 fibroblast overlap significantly with TADs from human hESC cells. (B) Human isochores overlap significantly with TADs from human cells. (C) Human isochores overlap significantly with TADs from IMR90 fibroblast. Fig S2. LocalZscore" as a function of shifted intervals. As soon as we displace the regions away from their original positions, TADs *vs*. isochores z-scores (A) drop considerably in a similar fashion to the TADs-hESC vs TADs-IMR90 z-scores (B). Fig S3. CTCF binding sites density. Density of 23,751 CTCF binding sites (from Li et al, 2013) in the five isochore families of the human genome. Fig S4. Isochores and chromatin subcompartments. The figure shows an average match between isochores/isochore blocks and chromatin subcompartments. Blue/Black triangles are examples of good/bad matches. An additional subcompartment called B4 was detected only in human chr19 (Rao et al, 2014), a chromosome composed (see Fig S42) mainly of GC-rich isochores and short/weak anchors to the lamina; its GC-poor chromatin configuration can be viewed as a particular case of compartment B3. Figs S5-S23. Chromatin interactions of mouse chromosomes. Chromatin interactions (from Vietri Rudan et al, 2013) are compared at a resolution of 250 Kb with the corresponding isochore profiles (see legend of Fig 1A for further information). H3 isochores are known to be very scarce in the mouse compared to the human genome; this makes the mouse genomic landscape less rugged and easier to be visually explored (this is so even for small chromosomes; see for instance chr18). A common emerging property is that intrachromosomal interactions have similar features across all mouse chromosomes; GC-poor isochores (L1 and L2 families) interact with each other, whereas GC-rich isochore interact only weakly (see for example chr8). Figs S24-S45. Chromatin interactions of human chromosomes. Chromatin interactions (from Rao et al, 2014) are compared at a resolution of 250 Kb with the corresponding isochore profiles (see Legend of Fig 2A for further information). The remarks made above about mouse chromosomes are equally valid for the human chromosomes, but in addition to the TADs/isochores co-mapping, LADs (from Kind et al, 2015) could also be compared. An almost perfect fit is observed between isochores boundaries and LAD borders; high contact frequencies (CF) values (>80%), correspond almost exclusively to the GC-poorest/gene-poorest isochores (L1), which very likely correspond to “stable” contact sites (Kind *et al*, 2015) with 20-fold lower gene density compared to regions with no nuclear lamina contacts (H2 and H3 isochores). The higher complexity of the human isochore mosaic compared to the mouse one, is also reflected in TAD signals, but the picture becomes clearer when LADs, TADs and isochores are simultaneously visualized and compared (see, for example, chr15, chr16, chr17 and chr21). In chr4, which is enriched in H1, L1 and L2 isochore families, H1 and L2 isochores are excluded from the lamina, whereas in chromosomes enriched in GC-rich isochores (see, for example, chr9, chr15, chr20), L2 and H1 (at low CF values) isochores are docked to the lamina. Notice that LADs from chr8 are not shown because the model system used to develop single-cell DamID experiments, haploid human myeloid leukemia cell line KBM7, is not haploid for chr8 and for a small part of chr15. Moreover, chr22 is not represented, as in these cell lines there is a balanced translocation between chr9 and chr22. chrX plus ChrY LAD maps were not reported in Kind et al, (2015). As a consequence, they are not represented.
(PDF)
