# Supplementary material for: An Isochore Framework Underlies Chromatin Architecture
Source: PLoS One. 2017 Jan 6;12(1):e0168023. doi: 10.1371/journal.pone.0168023 (PMC5218411; doi:10.1371/journal.pone.0168023)
Supplement: S1 File — Table A, Isochore families in the human genome, Table B, Structural and functional properties of the genome core vs. the genome desert. Table C, Isochores & interphase chromatin. (PDF) [file pone.0168023.s001.pdf]

1. Supplemental Tables

**TABLE A. ISOCHORE FAMILIES IN THE HUMAN GENOME [a]**					
	Amount %	Maxima GC	Minima GC	"Fixed" GC Ranges	"Extended" GC Ranges[b]
L1	19	35.5		33-37	→37.6
			37		
L2	36	38.7		37-41	36.4-42
			41		
H1	31	43.0		41-46	39.6-46.9
	**86**		46		
H2	11	48.5		46-53	45-54
			53		
H3	3	55.0		53-59	52→
	**14**				

(a) Modified from ref. 1. Values in bold are total amounts of L1+L2+H1 and H2+H3 families and correspond to the genome desert and the genome core, respectively.

(b) "Extended" ranges eliminate very small, local GC spikes (positive or negative) and avoid the oversegmentation linked to "fixed" ranges (Bernardi, in preparation).

TABLE B. STRUCTURAL AND FUNCTIONAL PROPERTIES OF THE GENOME CORE *VS.* THE GENOME DESERT [(a)]

**GC level**	**+**	**(2)**	**Housekeeping genes**	**+**	**(2)**
**LINE density**	**-**	**(3)**	**Developmental genes**	**-**	**(19,20)**
**SINE density**	**+**	**(4)**	**tRNA genes**	**+**	**(21)**
**Gene density**	**+**	**(5,6)**	**Reverse bands**	**+**	**(22-24)**
**Chromatin**	Open	**(7,8)**	**Nuclear location**	Central	**(7,8,25)**
**Gene expression**	**+**	**(9-11)**	**Translocations**	**+**	**(26)**
**Supercoiling**	**-**	**(12)**	**Fragile sites**	**+**	**(27,28)**
**GC-rich trinucleotides**	**+**	**(13,14)**	**Proviral integration**	**+**	**(2,29)**
**CpG, mC, CpG islands**	**+**	**(15,16)**	**Insertions/deletions**	**+**	**(30)**
**Isochore size**	**-**	**(17)**	**Recombination**	**+**	**(31)**
**GC heterogeneity**	**+**	**(17)**	**Point mutations**	**+**	**(32)**
**Intron, UTR size**	**-**	**(18)**	**Replication**	Early	**(33-36)**

(a) Modified from ref. 1. In general, the properties of the genome core are just opposite to those of the genome desert. +/- signs indicate positive/negative differences of the properties of the genome core compared to those of the genome desert.

TABLE C. ISOCHORES & INTERPHASE CHROMATIN[(a)]

**ISOCHORES**	
**Genome desert**[1]<br>(L1, L2, H1 families)<br>gene-poor, LINES<br>closed chromatin<br>late replicating<br>86% of the genome	**Genome core**[2]<br>(H2, H3 families)<br>gene-rich, SINES<br>open chromatin<br>early replicating<br>CpG islands, HK genes[(a)]
(av. size 0.9Mb)	
**COMPARTMENTS** [(37)]	
**B:** closed chromatin	**A:** open chromatin
**DOMAINS AND BORDERS/BOUNDARIES**	
**Domains:**<br>LADs, lamina-<br>-associated domains [(38)]<br>gene-poor, H3K27me3	**Borders:**<br>CTCF, promoters,<br>CpG islands<br>gene-rich, H3K4me2
**cLADs**, constitutive<br>LADs, GC-poor [(39)]	**ciLADs**, constitutive inter-LADs,<br>GC-rich
**Topological domains** [(21)]<br>91% of the genome<br>(av. size 0.88Mb)<br>Topologically associating<br>domains TADs[(40)]	**Boundaries** +<br>"**unorganized chromatin**"<br>(av. size 0.56 Mb) 9%of the genome<br>tRNAs, SINES, TSS[(b)]<br>Housekeeping genes
**Physical domains** [(c)] [(41,42)]<br>repressive marks	**Boundaries**<br>active marks
**CHROMATIN STATES** [(43)]	
Large-scale repressed<br>Repeat-associated	Promoter-associated<br>Transcription-associated<br>Active intergenic
**CHROMATIN TYPES** [(c)] [(44)]	
Blue/Green:<br>Heterochromatin<br>Polycomb/H3K27me3,<br>HPI/H3K9me2<br>Black: repressive chromatin<br>all late replicating	Red, Yellow:<br>active chromatin<br>H3K4me2, H3K79me3<br>both early replicating
**SUB-COMPARTMENTS** [(45)]	
B1, H3K27me3;<br>facultative heterochromatin;<br>replication→middle S phase<br>B2,B3, replication→end S phase	A1,A2, gene-rich,<br>high expression<br>H3K36me3, H3K79me2<br>H3K27ac, H3K4me1<br>both early-replicating
**CHROMATIN** [(46)]	
Heterochromatin<br>gene-poor, LINEs	Euchromatin<br>gene-rich, SINEs

(a) Modified from ref. 1; properties in red are shared or comparable between genome desert/genome core and the corresponding chromatin compartments, sub-compartments,

etc. (b) Abbreviations: HK, housekeeping; TSS, transcription start sites. (c) Drosophila. Other data concern mammalian cells.

References to Supplemental Tables S1-S3

1. Bernardi G (2015) Chromosome Architecture and Genome Organization. *PLoS One* 10: e0143739. http://dx.plos.org/10.1371/journal.pone.0143739
2. Bernardi G (2004) *Structural and evolutionary genomics: natural selection in genome evolution.* Elsevier, Amsterdam. This out-of-print book is freely available at www.giorgiobernardi.eu
3. Meunier-Rotival M, Soriano P, Cuny G, Strauss F, Bernardi G (1982) Sequence organization and genomic distribution of the major family of interspersed repeats of mouse DNA. *Proc Natl Acad Sci U S A* 79: 355–359.
4. Soriano P, Meunier-Rotival M, Bernardi G (1983) The distribution of interspersed repeats is nonuniform and conserved in the mouse and human genomes. *Proc Natl Acad Sci U S A* 80: 1816–1820.
5. Mouchiroud D, D'Onofrio G, Aissani B, Macaya G, Gautier C, Bernardi G (1991) The distribution of genes in the human genome. *Gene* 100: 181–187.
6. Zoubak S, Clay O, Bernardi G (1996) The gene distribution of the human genome. *Gene* 174: 95–102.
7. Saccone S, Federico C, Bernardi G (2002) Localization of the gene-richest and the gene-poorest isochores in the interphase nuclei of mammals and birds. *Gene* 300: 169–178.
8. Gilbert N, Boyle S, Fiegler H, Woodfine K, Carter NP, Bickmore WA (2004) Chromatin architecture of the human genome: Gene-rich domains are enriched in open chromatin fibers. *Cell* 118: 555–566.
9. Caron H, van Schaik B, van der Mee M, Baas F, Riggins G, van Sluis P, Hermus MC, van Asperen R, Boon K, Voûte P, Heisterkamp S, van Kampen a & Versteeg R (2001) The human transcriptome map: clustering of highly expressed genes in chromosomal domains. *Science* 291**:** 1289–1292
10. Konu O, Li MD (2002) Correlations between mRNA expression levels and GC contents of coding and untranslated regions of genes in rodents. *J Mol Evol* 54: 35–41.
11. Versteeg R, van Schaik BDC, van Batenburg MF, Roos M, Monajemi R, Caron H, Bussemaker HJ, van Kampen AHC (2003) The human transcriptome map reveals extremes in gene dentistry, intron length, GC content, and repeat pattern for domains of highly and weakly expressed genes. *Genome Res* 13: 1998–2004.
12. Naughton C, Avlonitis N, Corless S, Prendergast JG, Mati IK, Eijk PP, Cockroft SL, Bradley M, Ylstra B, Gilbert N (2013) Transcription forms and remodels supercoiling domains unfolding large-scale chromatin structures. *Nat Struct Mol Biol* 20: 387–395.
13. Costantini M, Bernardi G (2008) The short-sequence designs of isochores from the human genome. *Proc Natl Acad Sci* 105: 13971–13976.
14. Arhondakis S, Auletta F, Bernardi G (2011) Isochores and the regulation of gene expression in the human genome. *Genome Biol Evol* 3: 1080–1089.
15. Jabbari K, Bernardi G (2004) Cytosine methylation and CpG, TpG (CpA) and TpA frequencies. *Gene* 333: 143–149.
16. Varriale A, Bernardi G (2010) Distribution of DNA methylation, CpGs, and CpG islands in human isochores. *Genomics* 95: 25–28.

17. Costantini M, Clay O, Auletta F, Bernardi G (2006) An isochore map of human chromosomes. *Genome Res* 16: 536–541.
18. Duret L, Mouchiroud D, Gautier C (1995) Statistical analysis of vertebrate sequences reveals that long genes are scarce in GC-rich isochores. *J Mol Evol* 40: 308–317.
19. Kikuta H, Laplante M, Navratilova P, Komisarczuk AZ, Engstrom PG, Fredman D, Akalin A, Caccamo M, Sealy I, Howe K, Ghislain J, Pezeron G, Mourrain P, Ellingsen S, Oates AC, Thisse C, Thisse B, Foucher I, Adolf B, Geling A, et al (2007) Genomic regulatory blocks encompass multiple neighboring genes and maintain conserved synteny in vertebrates. *Genome Res.* 17**:** 545–555
20. Jabbari K, Nürnberg P (2016) A genomic view on epilepsy and autism candidate genes. *Genomics* 108: 31–36.
21. Dixon JR, Selvaraj S, Yue F, Kim A, Li Y, Shen Y, Hu M, Liu JS, Ren B (2012) Topological domains in mammalian genomes identified by analysis of chromatin interactions. *Nature* 485: 376–380.
22. Saccone S, De Sario A, Della Valle G, Bernardi G (1992) The highest gene concentrations in the human genome are in telomeric bands of metaphase chromosomes. *Proc Natl Acad Sci U S A* 89: 4913–4917.
23. Saccone S, Pavliček A, Federico C, Pačes J, Bernardi G (2001) Genes, isochores and bands in human chromosomes 21 and 22. *Chromosom Res* 9: 533–539.
24. Costantini M, Clay O, Federico C, Saccone S, Auletta F, Bernardi G (2007) Human chromosomal bands: Nested structure, high-definition map and molecular basis. *Chromosoma* 116: 29–40.
25. Sadoni N, Langer S, Fauth C, Bernardi G, Cremer T, Turner BM, Zink D (1999) Nuclear organization of mammalian genomes: Polar chromosome territories build up functionally distinct higher order compartments. *J Cell Biol* 146: 1211–1226.
26. Fisher AM, Strike P, Scott C, Moorman A V (2005) Breakpoints of variant 9;22 translocations in chronic myeloid leukemia locate preferentially in the CG-richest regions of the genome. *Genes Chromosom Cancer* 43: 383–389.
27. Lemaitre C, Zaghloul L, Sagot M-F, Gautier C, Arneodo A, Tannier E, Audit B (2009) Analysis of fine-scale mammalian evolutionary breakpoints provides new insight into their relation to genome organisation. *BMC Genomics* 10: 335.
28. Berthelot C, Muffato M, Abecassis J, Roest Crollius H (2015) The 3D organization of chromatin explains evolutionary fragile genomic regions. *Cell Rep* 10: 1913–1924.
29. Rynditch A V., Zoubak S, Tsyba L, Tryapitsina-Guley N, Bernardi G (1998) The regional integration of retroviral sequences into the mosaic genomes of mammals. *Gene* 222: 1–16.
30. Costantini M, Bernardi G (2009) Mapping insertions, deletions and SNPs on Venter's chromosomes. *PLoS One* doi.org/10.1371/journal.pone.0005972
31. Fullerton SM, Bernardo Carvalho A, Clark AG (2001) Local rates of recombination are positively correlated with GC content in the human genome. *Mol Biol Evol* 18: 1139–1142.
32. Hodgkinson A, Chen Y, Eyre-Walker A (2012) The large-scale distribution of somatic mutations in cancer genomes. *Hum Mutat* 33: 136–143.
33. Watanabe Y, Fujiyama A, Ichiba Y, Hattori M, Yada T, Sakaki Y, Ikemura T (2002) Chromosome-wide assessment of replication timing for human chromosomes 11q and 21q: disease-related genes in timing-switch regions. *Hum Mol Genet* 11: 13–21.
34. Schmegner C, Hoegel J, Vogel W, Assum G (2007) The rate, not the spectrum, of base pair substitutions changes at a GC-content transition in the human NF1 gene region: implications for the evolution of the mammalian genome structure. *Genetics* 175: 421–428.

35. Costantini M, Bernardi G (2008) Replication timing, chromosomal bands, and isochores. *Proc Natl Acad Sci U S A* 105: 3433–3437.
36. Pope BD, Ryba T, Dileep V, Yue F, Wu W, Denas O, Vera DL, Wang Y, Hansen RS, Canfield TK, Thurman RE, Cheng Y, Gülsoy G, Dennis JH, Snyder MP, Stamatoyannopoulos JA, Taylor J, Hardison RC, Kahveci T, Ren B, *et al* (2014) Topologically associating domains are stable units of replication-timing regulation. *Nature* 515**:** 402–405 Available at:
37. Lieberman-Aiden E, van Berkum NL, Williams L, Imakaev M, Ragoczy T, Telling A, Amit I, Lajoie BR, Sabo PJ, Dorschner MO, Sandstrom R, Bernstein B, Bender MA, Groudine M, Gnirke A, Stamatoyannopoulos J, Mirny LA, Lander ES & Dekker J (2009) Comprehensive mapping of long-range interactions reveals folding principles of the human genome. *Science* 326**:** 289–93
38. Guelen L, Pagie L, Brasset E, Meuleman W, Faza MB, Talhout W, Eussen BH, de Klein A, Wessels L, de Laat W & van Steensel B (2008) Domain organization of human chromosomes revealed by mapping of nuclear lamina interactions. *Nature* 453**:** 948–51
39. Meuleman W, Peric-Hupkes D, Kind J, Beaudry JB, Pagie L, Kellis M, Reinders M, Wessels L, Van Steensel B (2013) Constitutive nuclear lamina-genome interactions are highly conserved and associated with A/T-rich sequence. *Genome Res* 23: 270–280.
40. Nora EP, Lajoie BR, Schulz EG, Giorgetti L, Okamoto I, Servant N, Piolot T, van Berkum NL, Meisig J, Sedat J, Gribnau J, Barillot E, Blüthgen N, Dekker J & Heard E (2012) Spatial partitioning of the regulatory landscape of the X-inactivation centre. *Nature* 485**:** 381–385
41. Hou C, Li L, Qin ZS, Corces VG (2012) Gene Density, Transcription, and Insulators Contribute to the Partition of the Drosophila Genome into Physical Domains. *Mol Cell* 48: 471–484.
42. Sexton T, Yaffe E, Kenigsberg E, Bantignies F, Leblanc B, Hoichman M, Parrinello H, Tanay A, Cavalli G (2012) Three-dimensional folding and functional organization principles of the Drosophila genome. *Cell* 148: 458–472.
43. Ernst J, Kellis M (2012) ChromHMM: automating chromatin-state discovery and characterization. *Nat Methods* 9: 215–216.
44. Filion GJ, van Bemmel JG, Braunschweig U, Talhout W, Kind J, Ward LD, Brugman W, de Castro IJ, Kerkhoven RM, Bussemaker HJ & van Steensel B (2010) Systematic Protein Location Mapping Reveals Five Principal Chromatin Types in Drosophila Cells. *Cell* 143**:** 212–224
45. Rao SSP, Huntley MH, Durand NC, Stamenova EK, Bochkov ID, Robinson JT, Sanborn AL, Machol I, Omer AD, Lander ES & Aiden EL (2014) A 3D map of the human genome at kilobase resolution reveals principles of chromatin looping. *Cell* 159**:** 1665–1680
46. Solovei I, Thanisch K, Feodorova Y (2016) How to rule the nucleus: divide et impera. *Curr Opin Cell Biol* 40: 47–59.
