# Supplementary material for: An Isochore Framework Underlies Chromatin Architecture
Source: PLoS One. 2017 Jan 6;12(1):e0168023. doi: 10.1371/journal.pone.0168023 (PMC5218411; doi:10.1371/journal.pone.0168023)

Fig. S1A

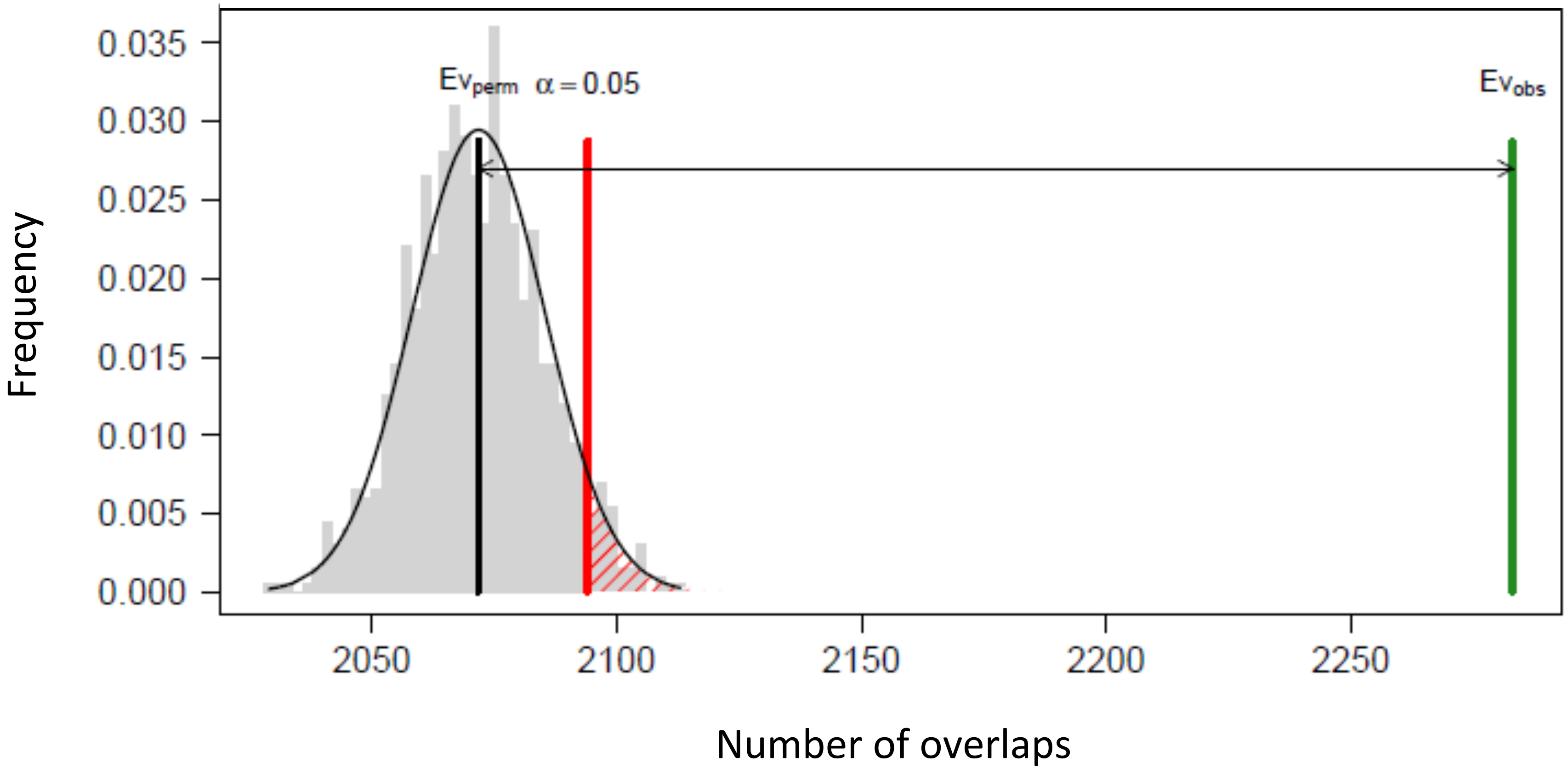

Frequency
0.035
0.030
0.025
0.020
0.015
0.010
0.005
0.000
Ev_perm
α = 0.05
Ev_obs
2050
2100
2150
2200
2250
Number of overlaps

Fig. S1B

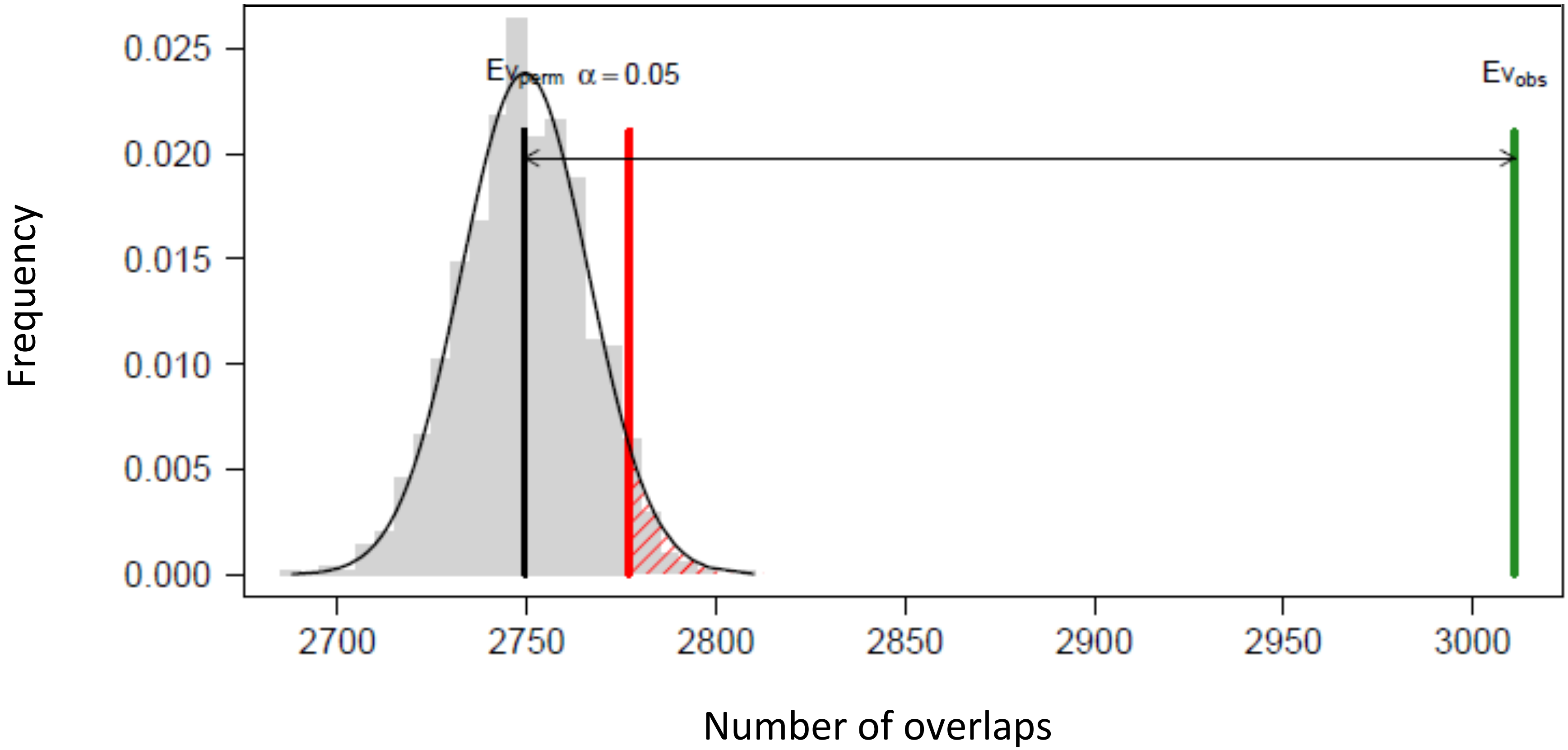
0.025
0.020
0.015
0.010
0.005
0.000
Frequency
Ev_{perm}
$\alpha = 0.05$
Ev_{obs}
2700
2750
2800
2850
2900
2950
3000
Number of overlaps

Fig. S1C

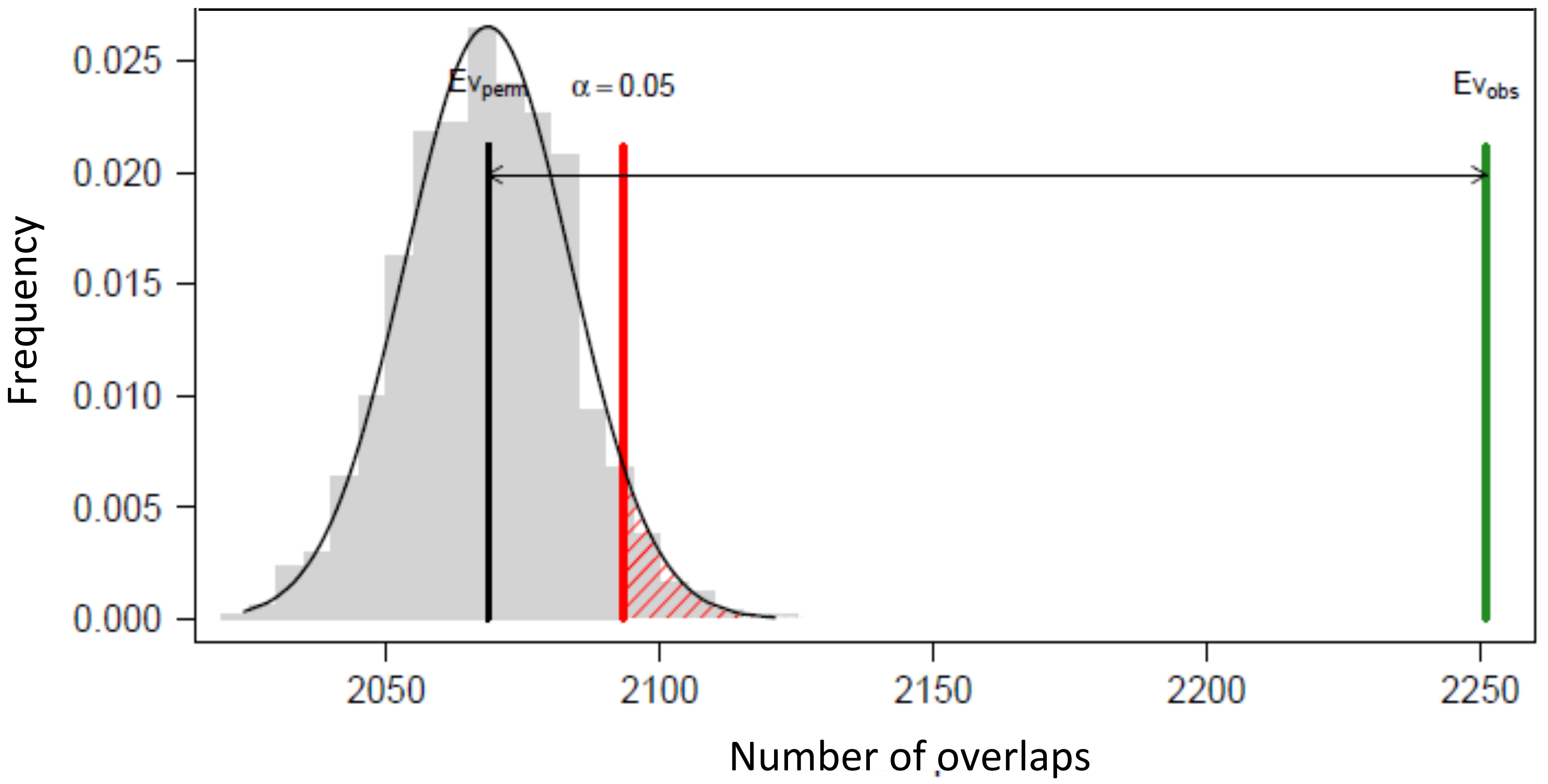

0.025
0.020
0.015
0.010
0.005
0.000
Frequency
Ev_{perm}
$\alpha = 0.05$
Ev_{obs}
2050
2100
2150
2200
2250
Number of overlaps

Fig. S2

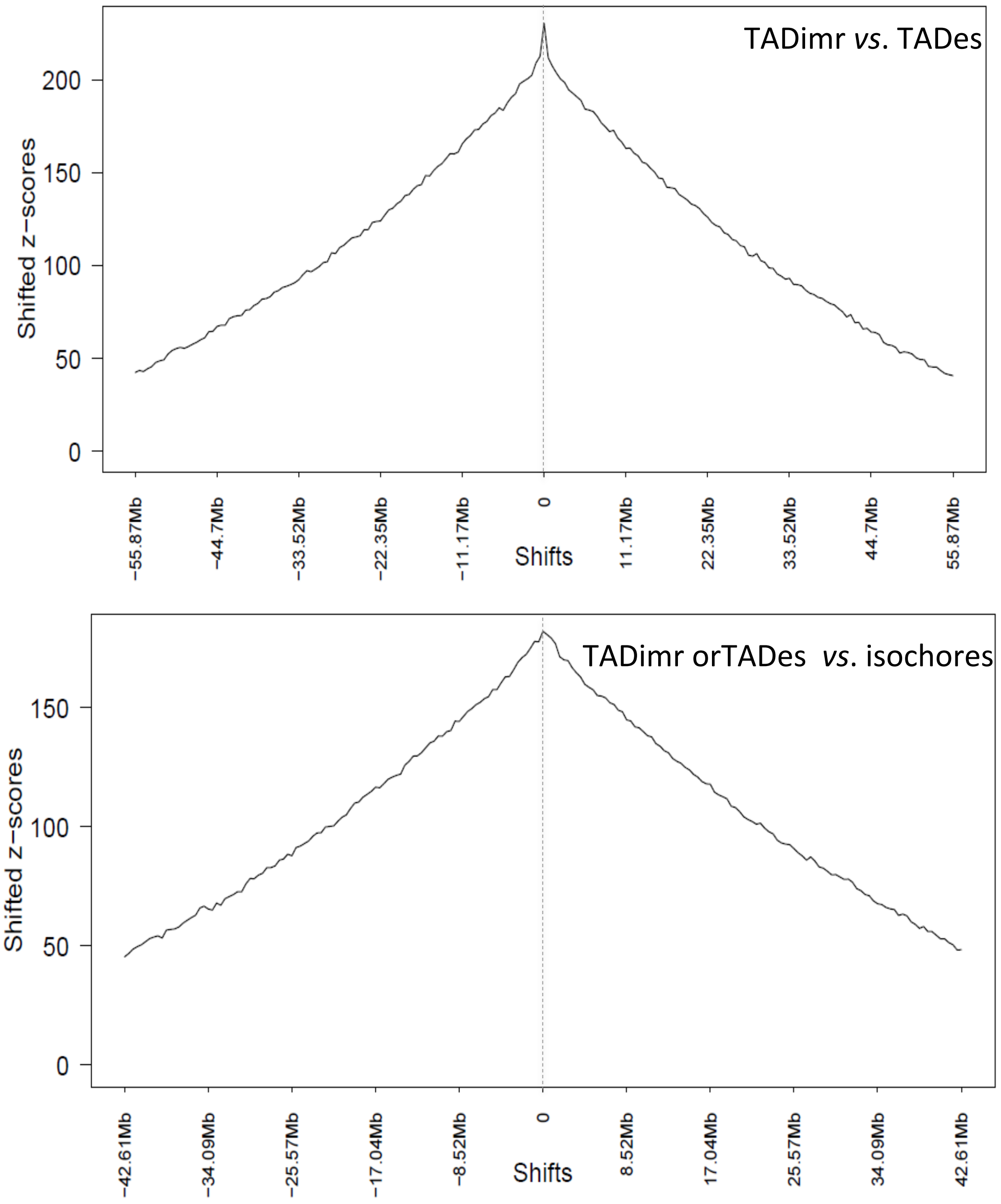

(A)
TADimr *vs*. TADes
Shifted z−scores
0
50
100
150
200
Shifts
−55.87Mb
−44.7Mb
−33.52Mb
−22.35Mb
−11.17Mb
0
11.17Mb
22.35Mb
33.52Mb
44.7Mb
55.87Mb
(B)
TADimr orTADes *vs*. isochores
Shifted z−scores
0
50
100
150
Shifts
−42.61Mb
−34.09Mb
−25.57Mb
−17.04Mb
−8.52Mb
0
8.52Mb
17.04Mb
25.57Mb
34.09Mb
42.61Mb

Fig. S3

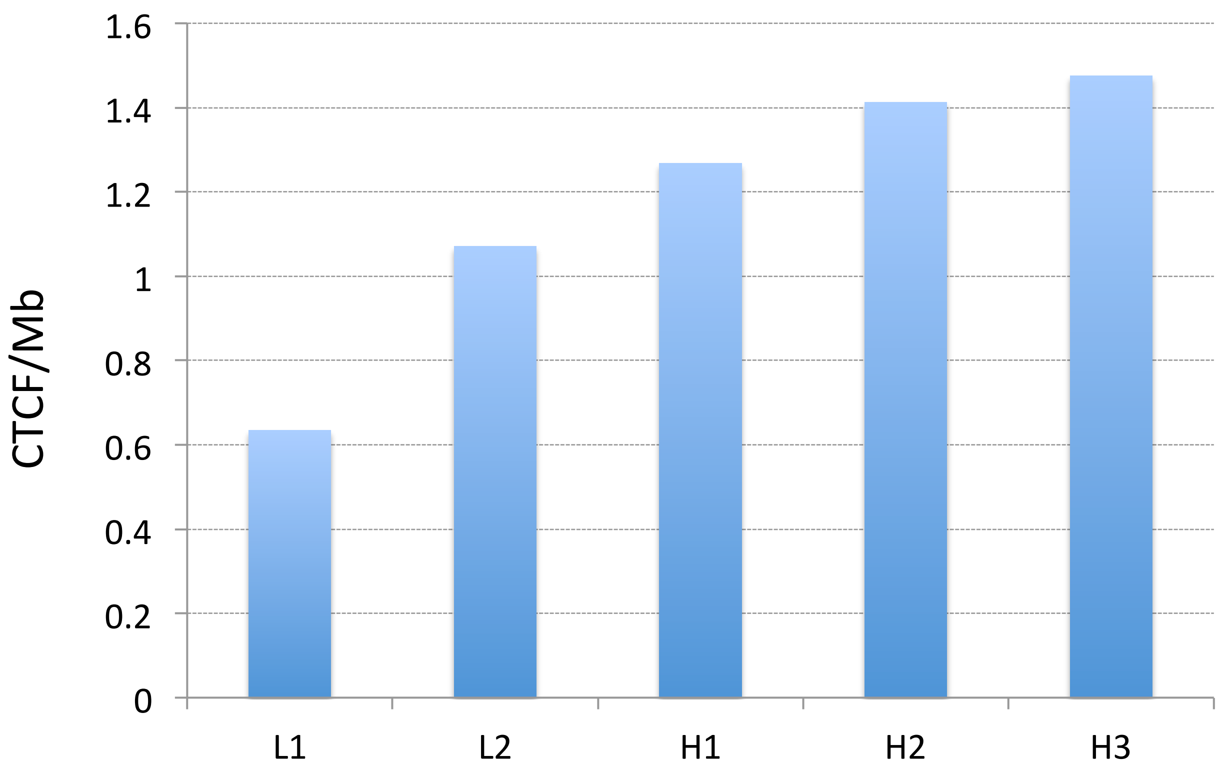

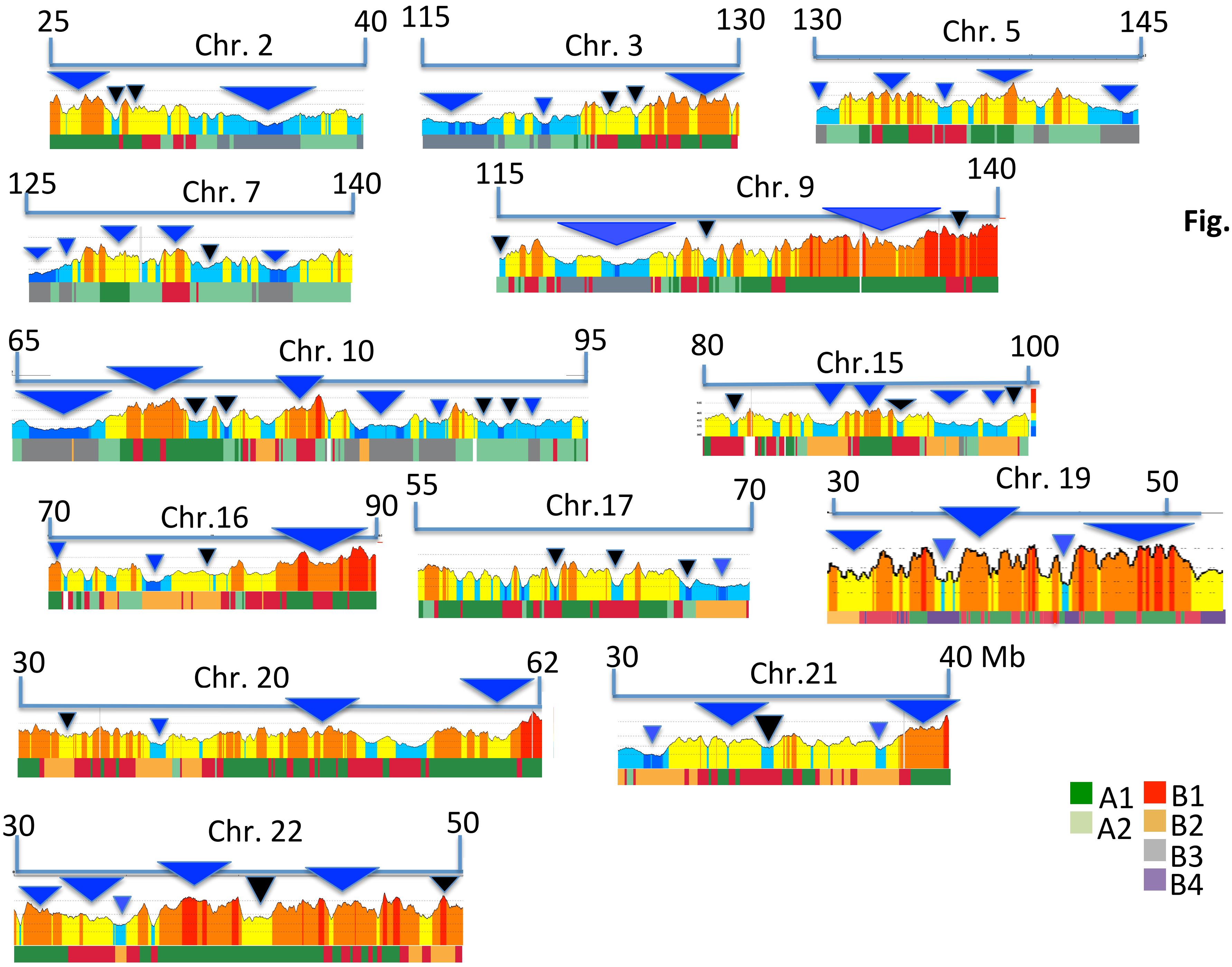
25
Chr. 2
40
115
Chr. 3
130
130
Chr. 5
145
125
Chr. 7
140
115
Chr. 9
140
65
Chr. 10
95
80
Chr.15
100
70
Chr.16
90
55
Chr.17
70
30
Chr. 19
50
30
Chr. 20
62
30
Chr.21
40 Mb
30
Chr. 22
50
A1
A2
B1
B2
B3
B4

Fig. S4

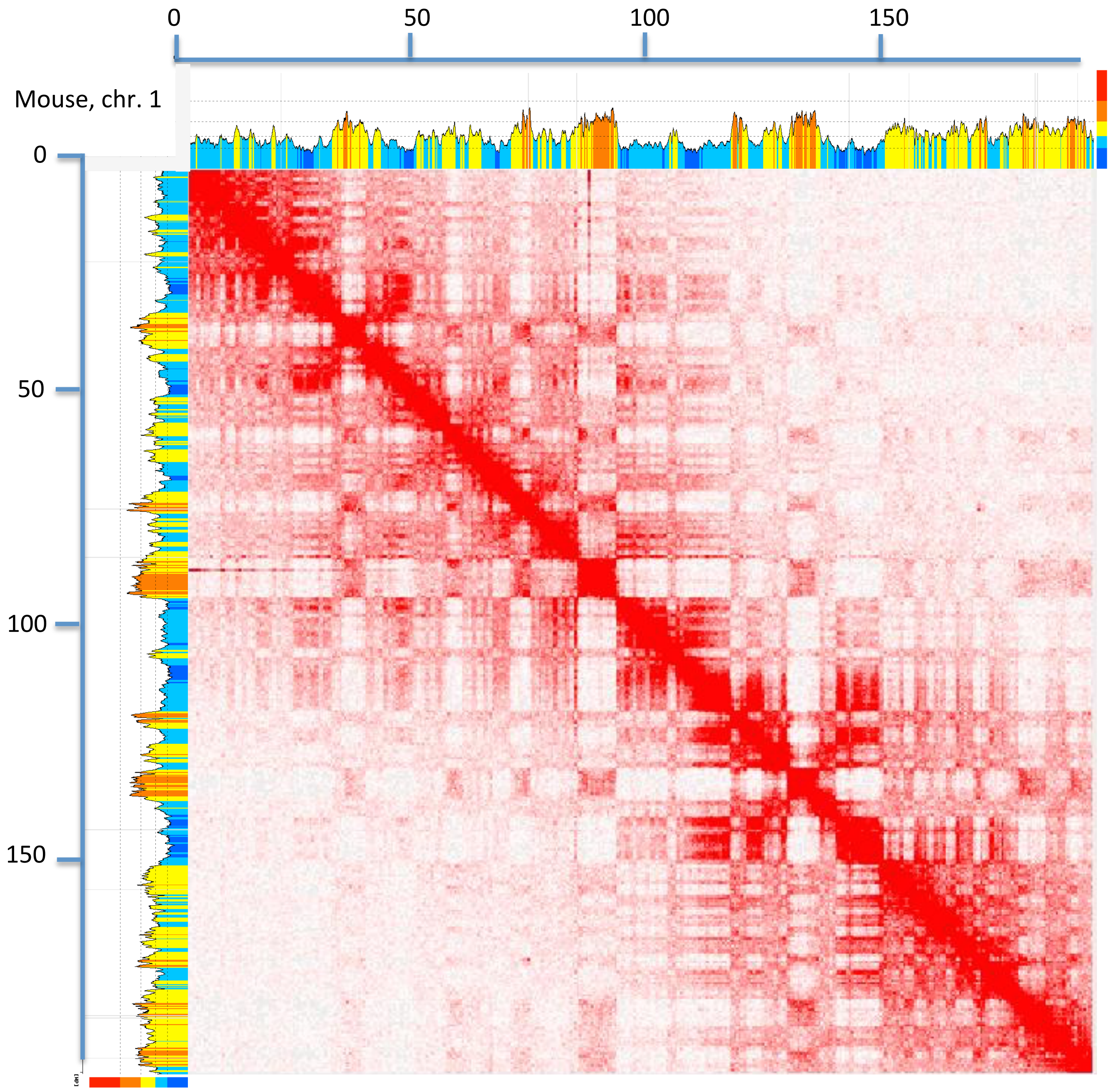

Fig. S5

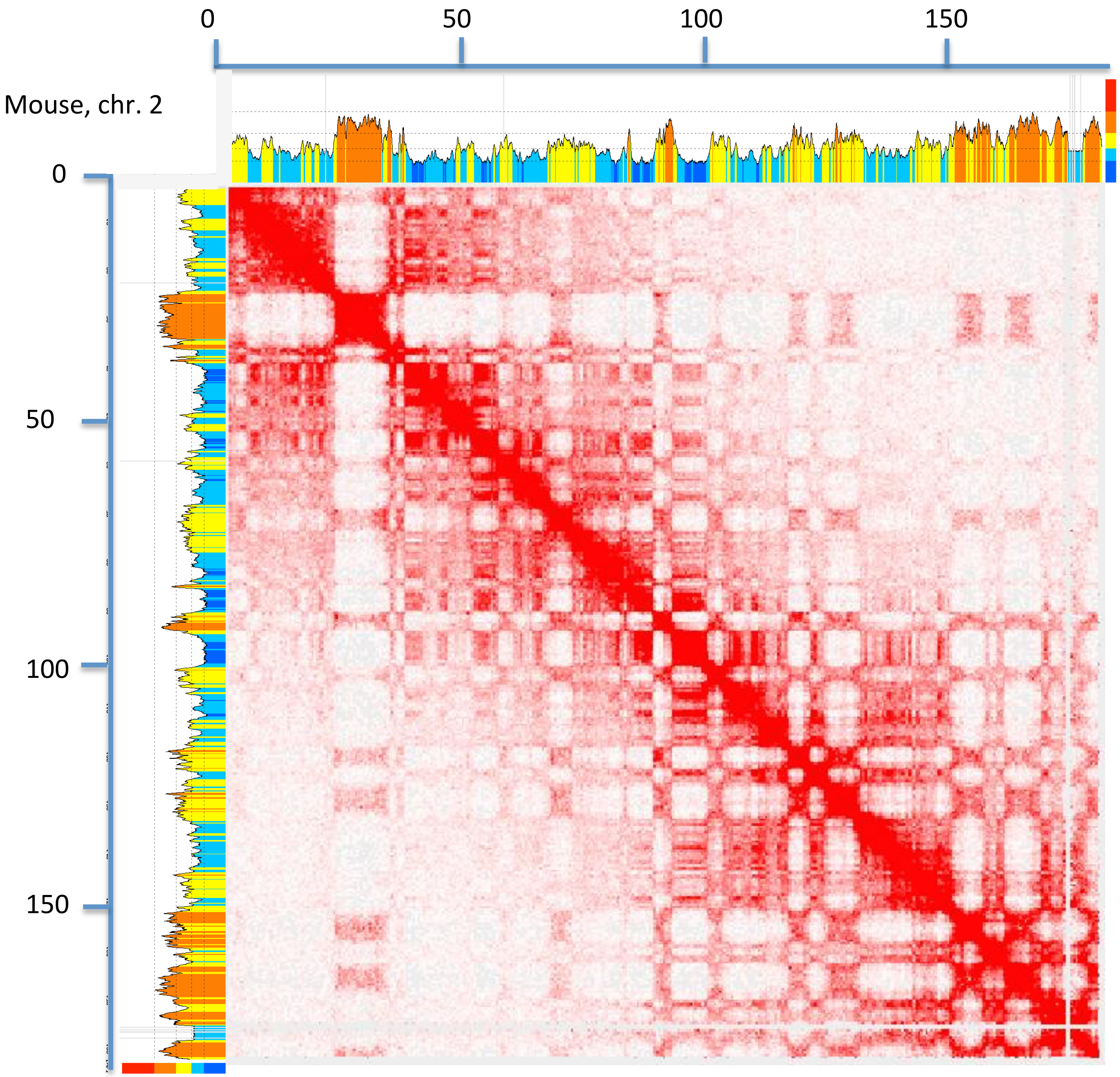

Fig. S6

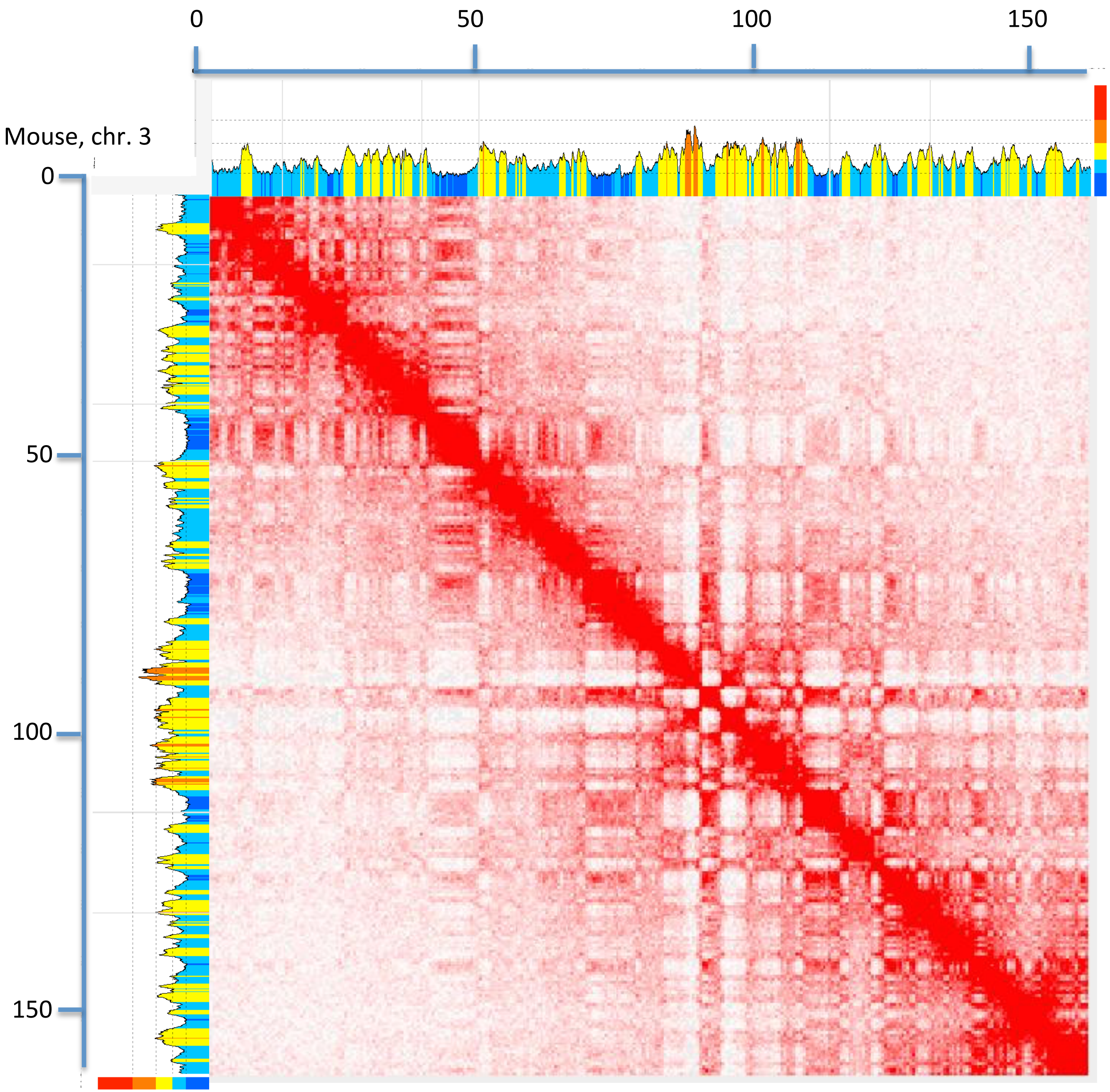

Fig. S7

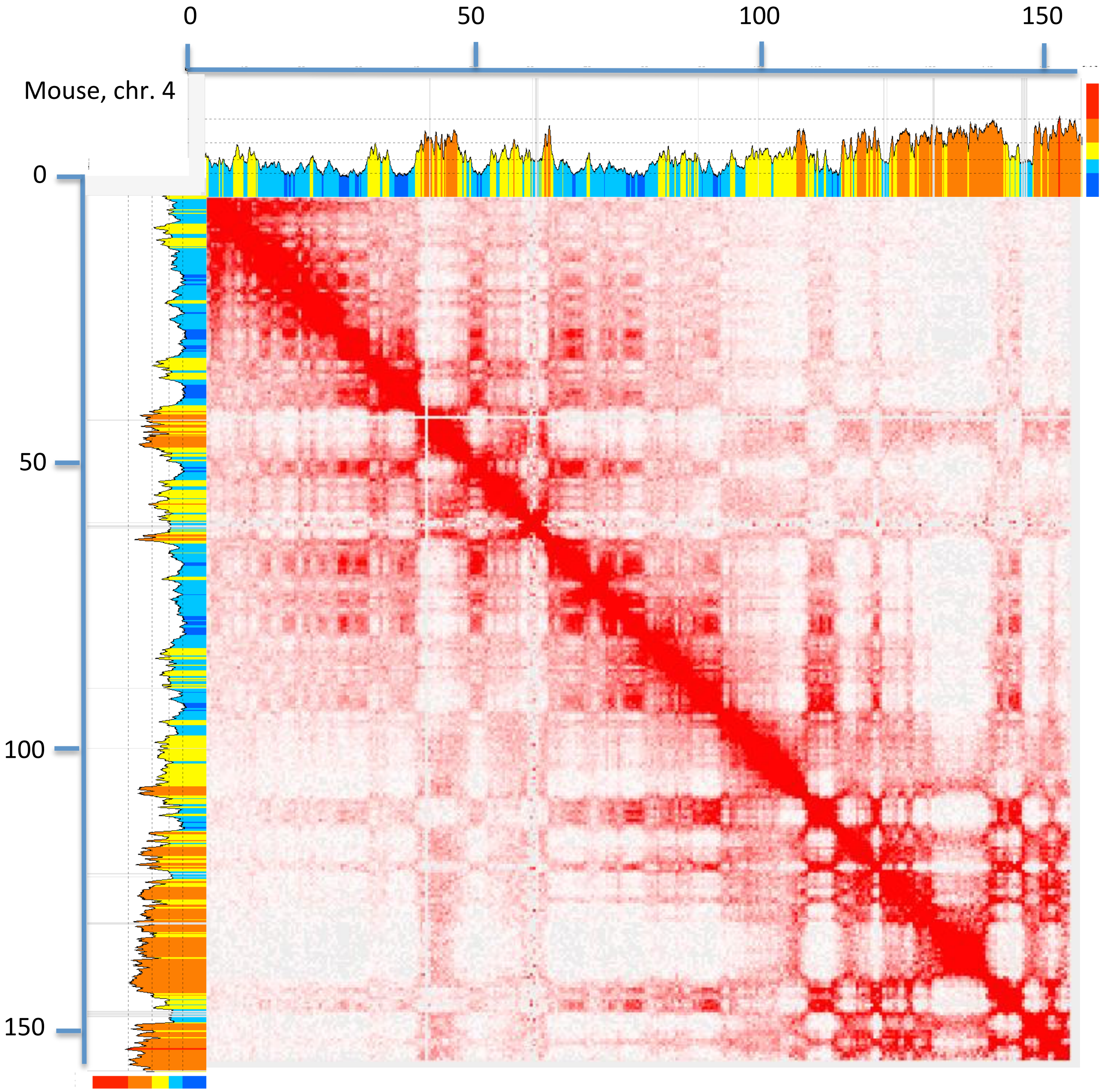

Fig. S8

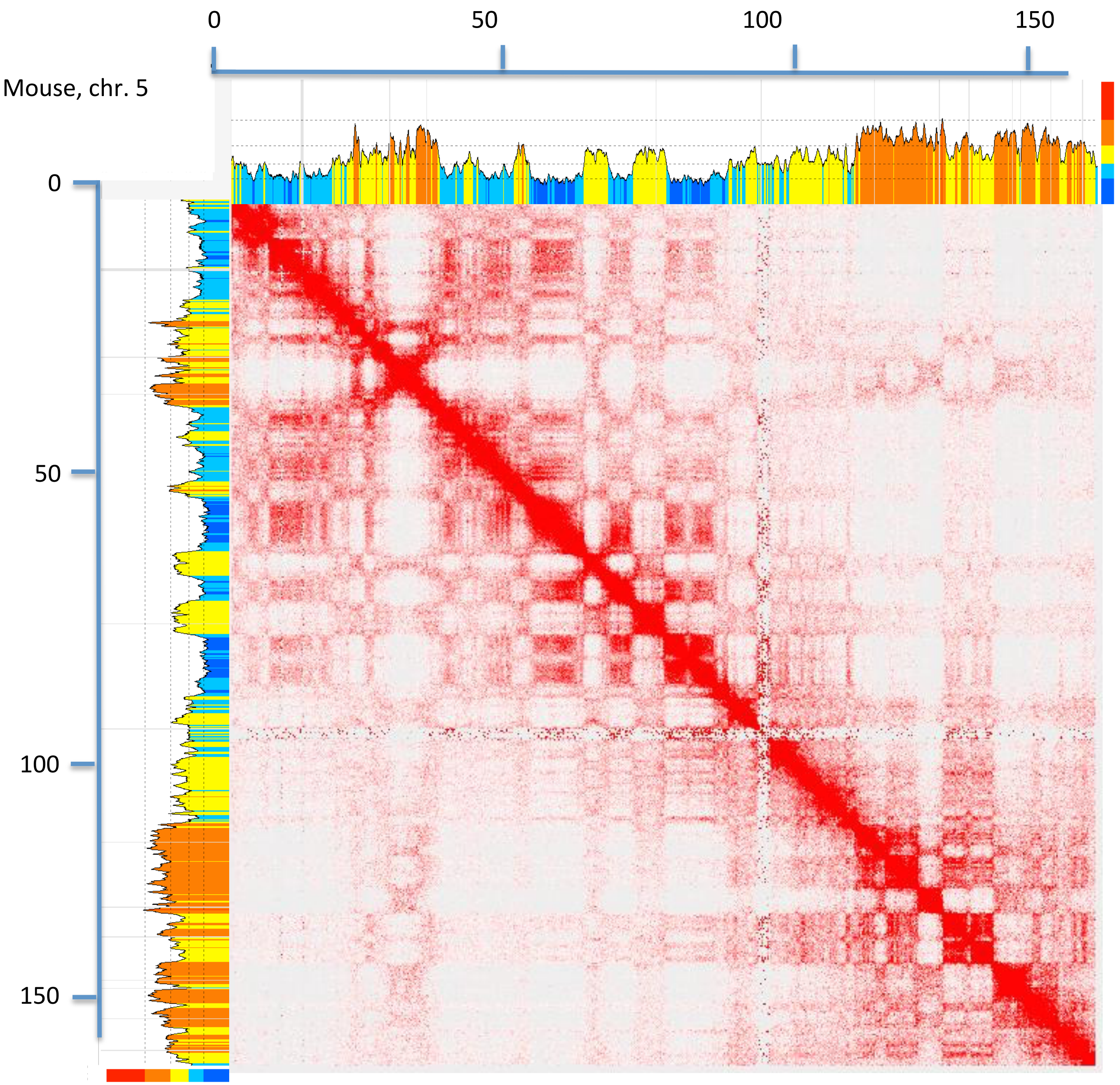

Fig. S9

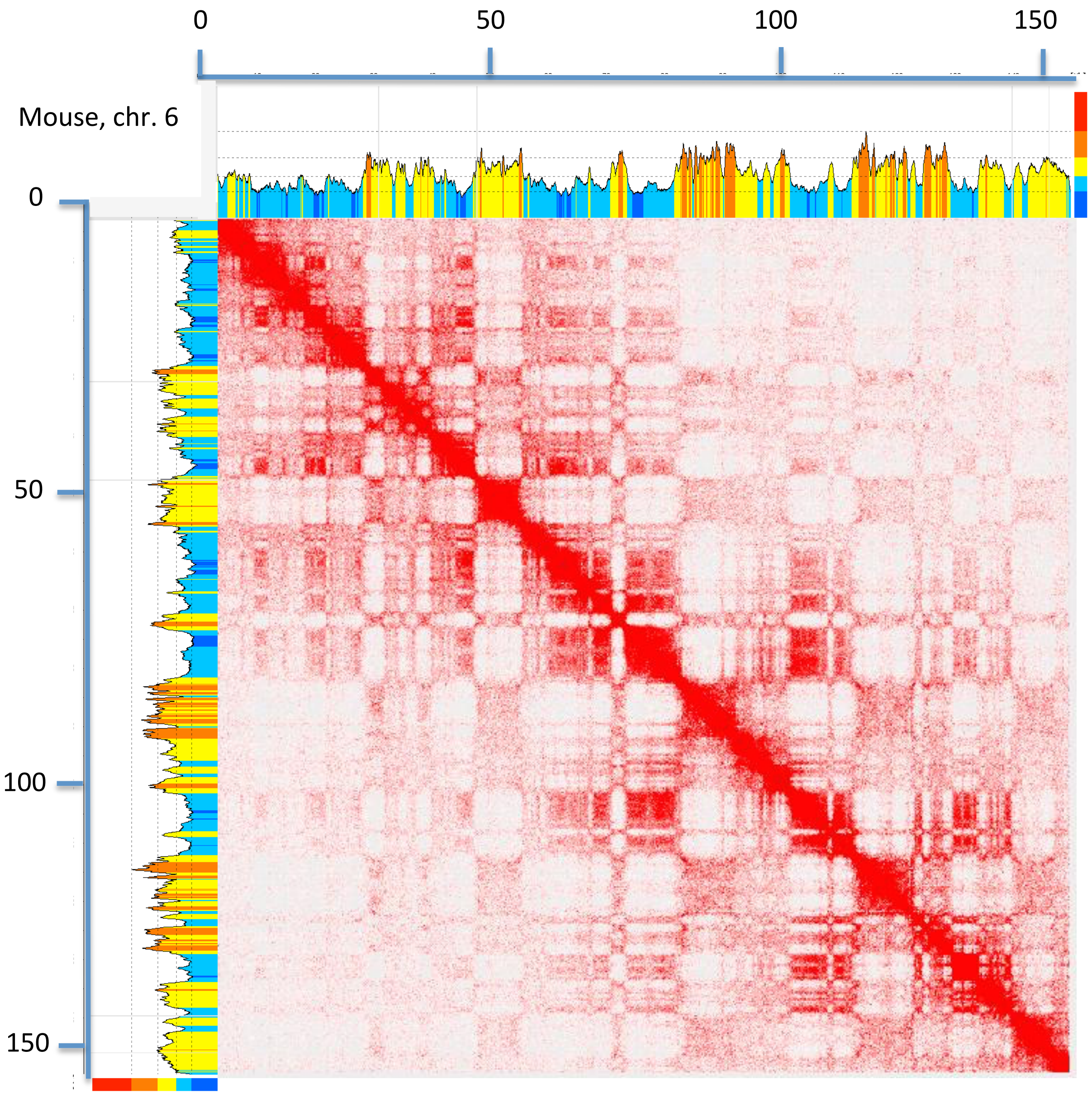

Fig. S10

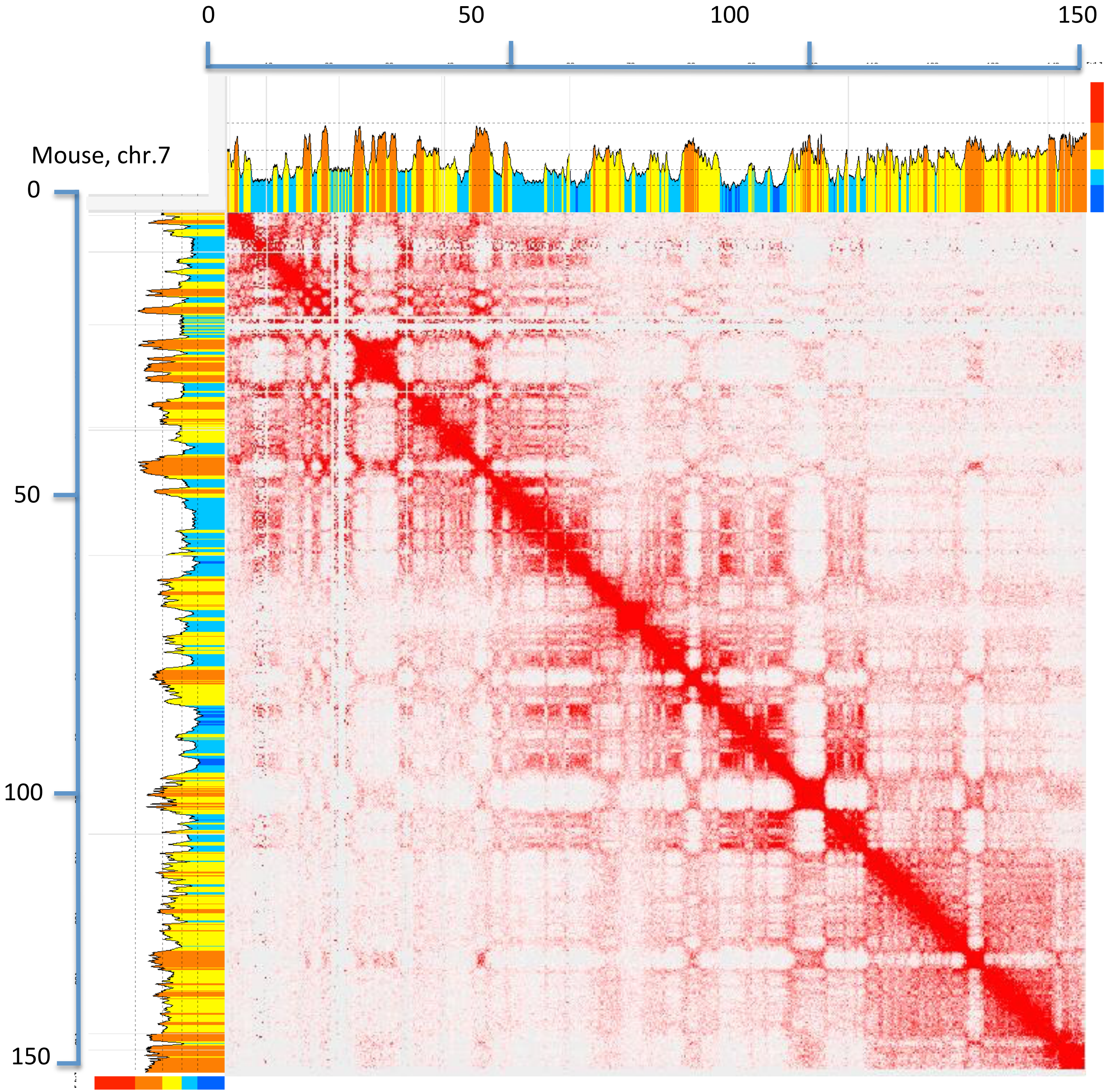

Fig. S11

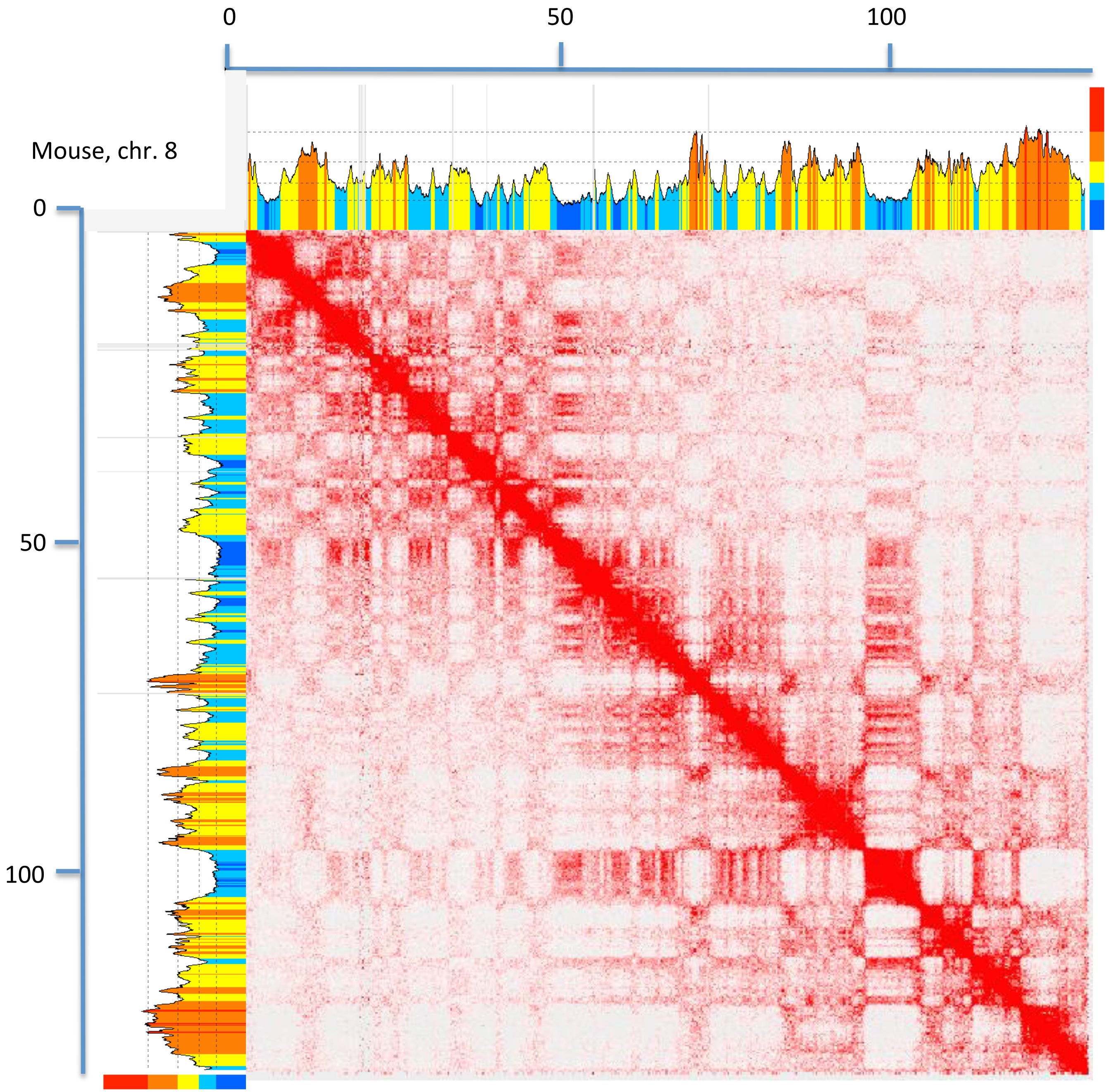

Fig. S12

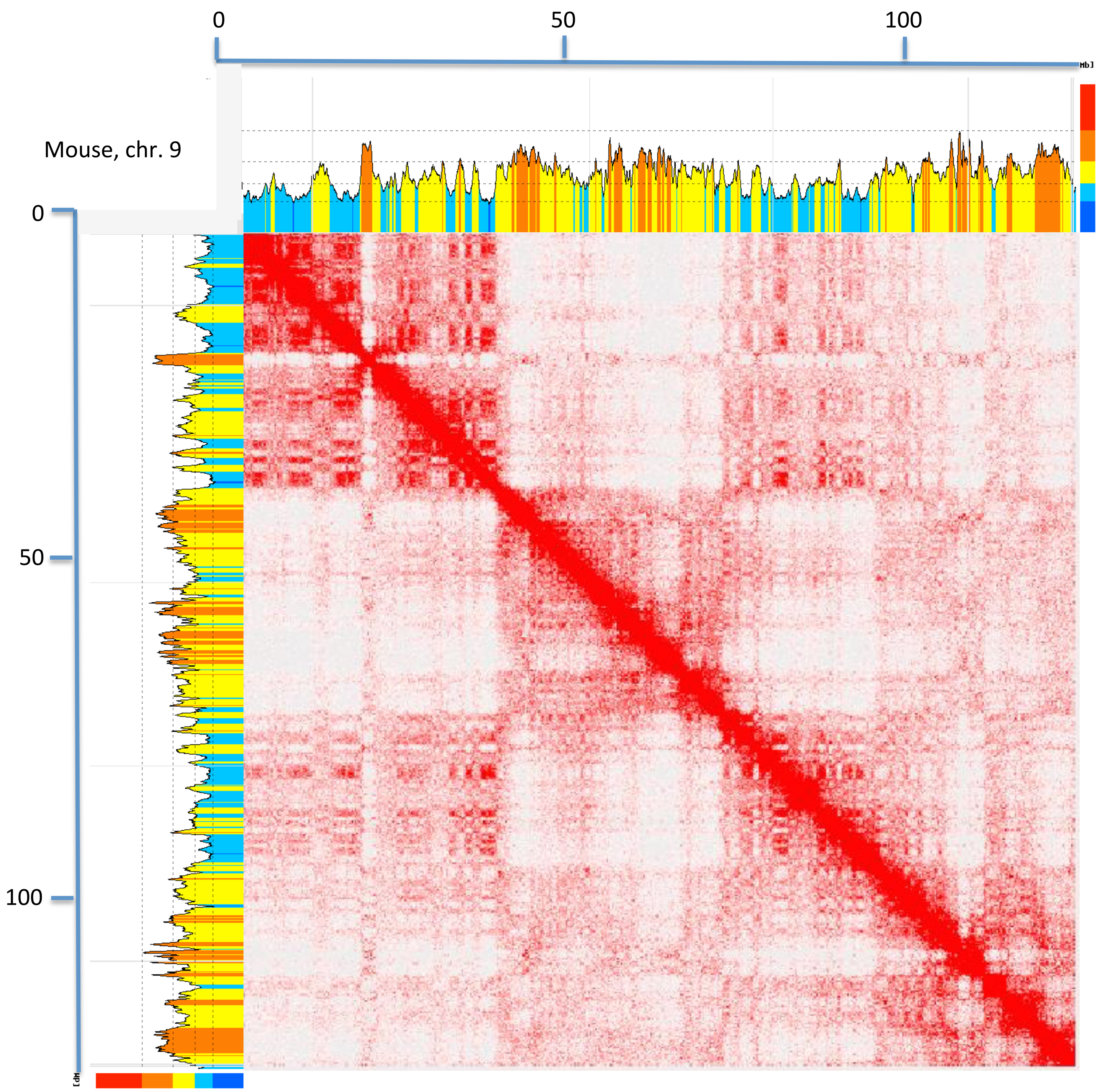

Fig. S13

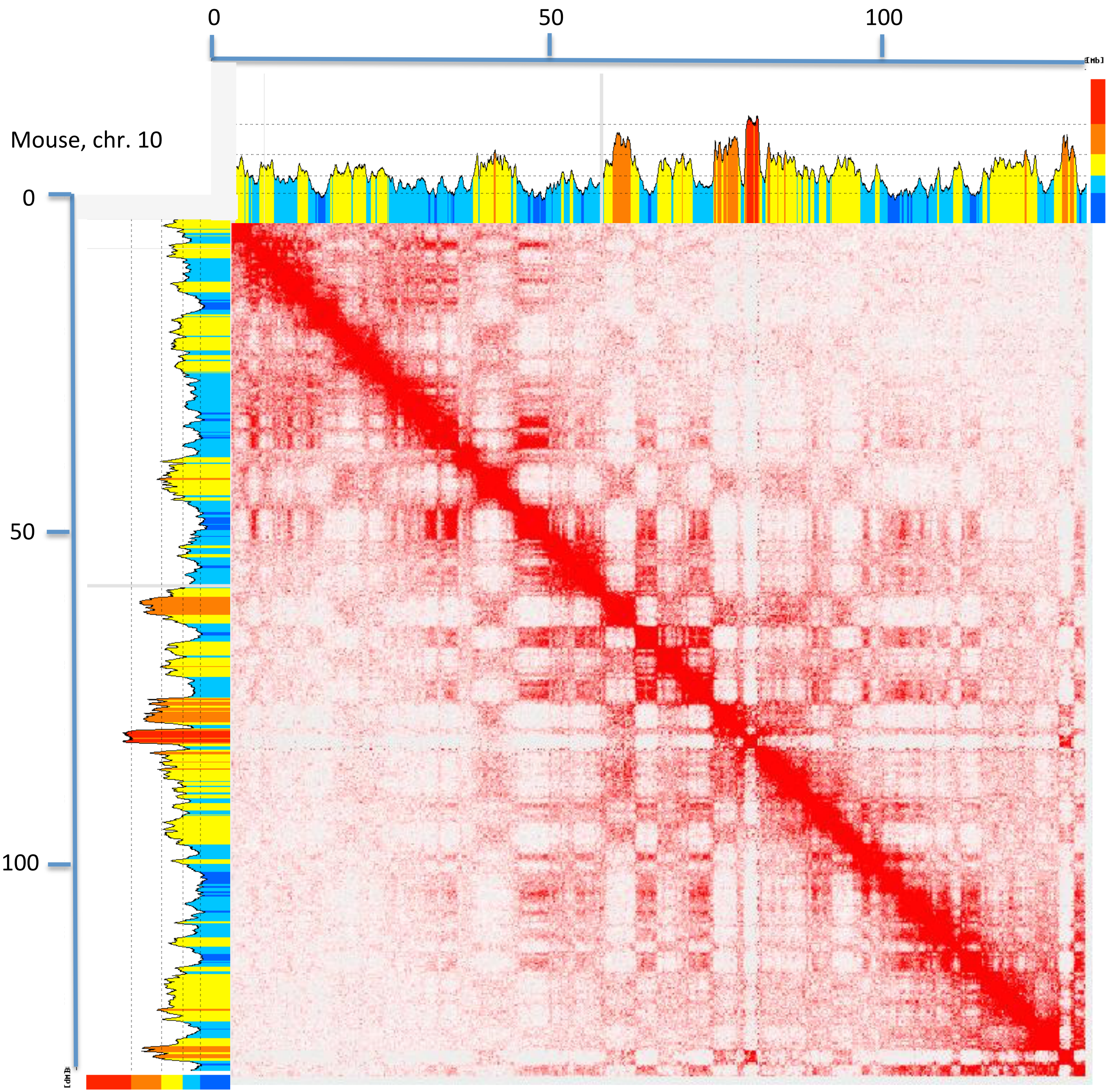

Fig. S14

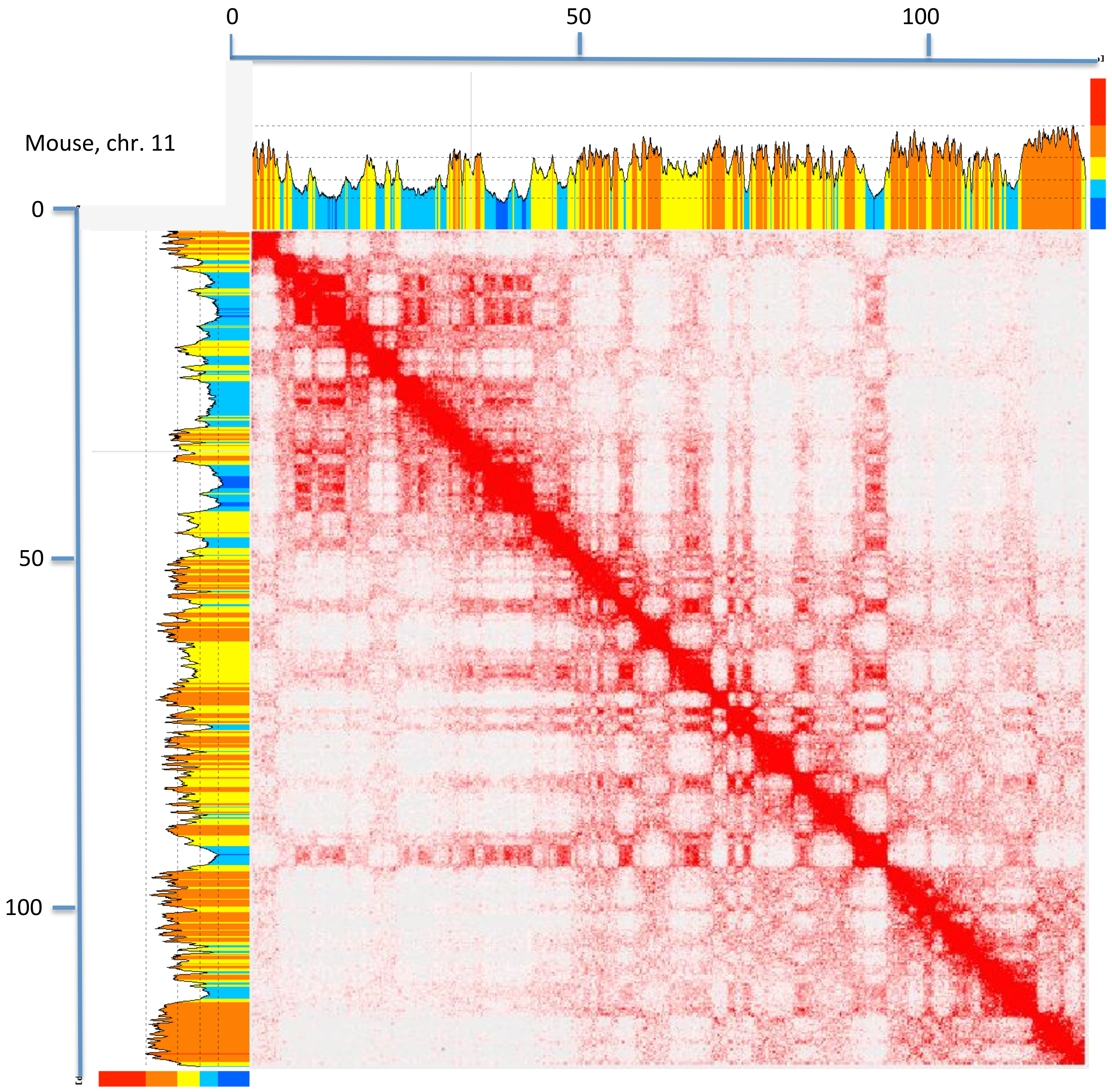

Fig. S15

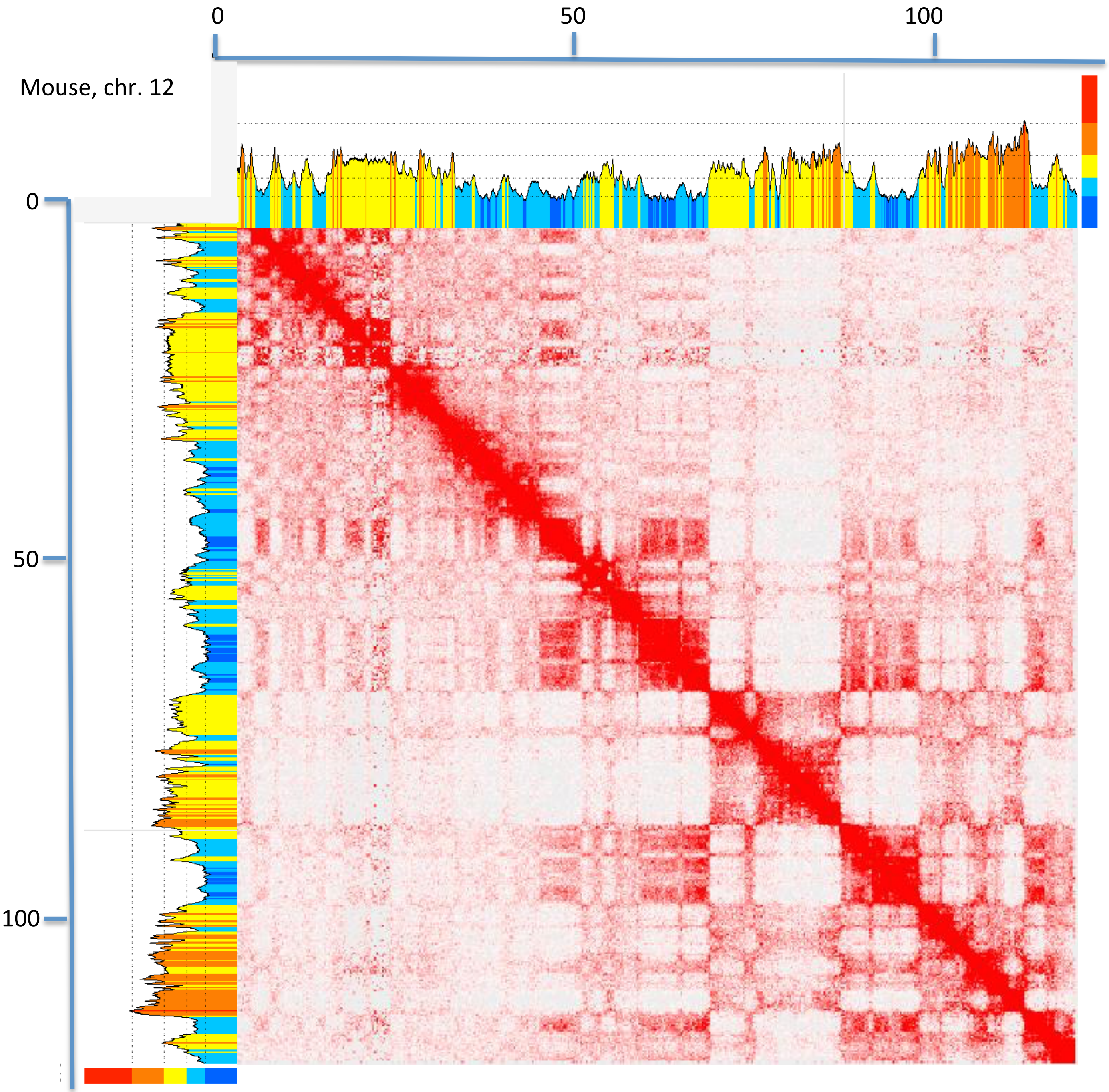

Fig. S16

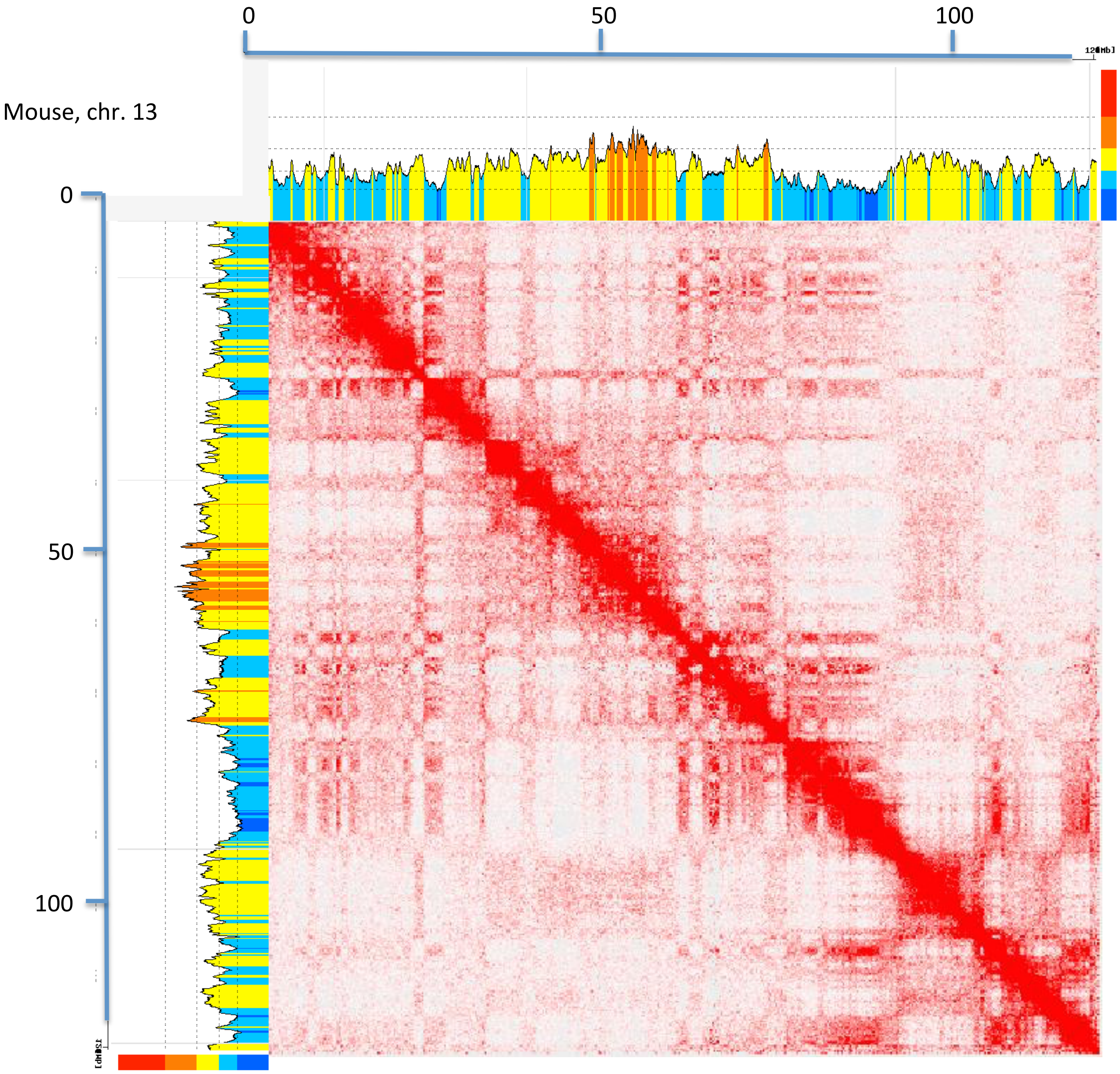

Fig. S17

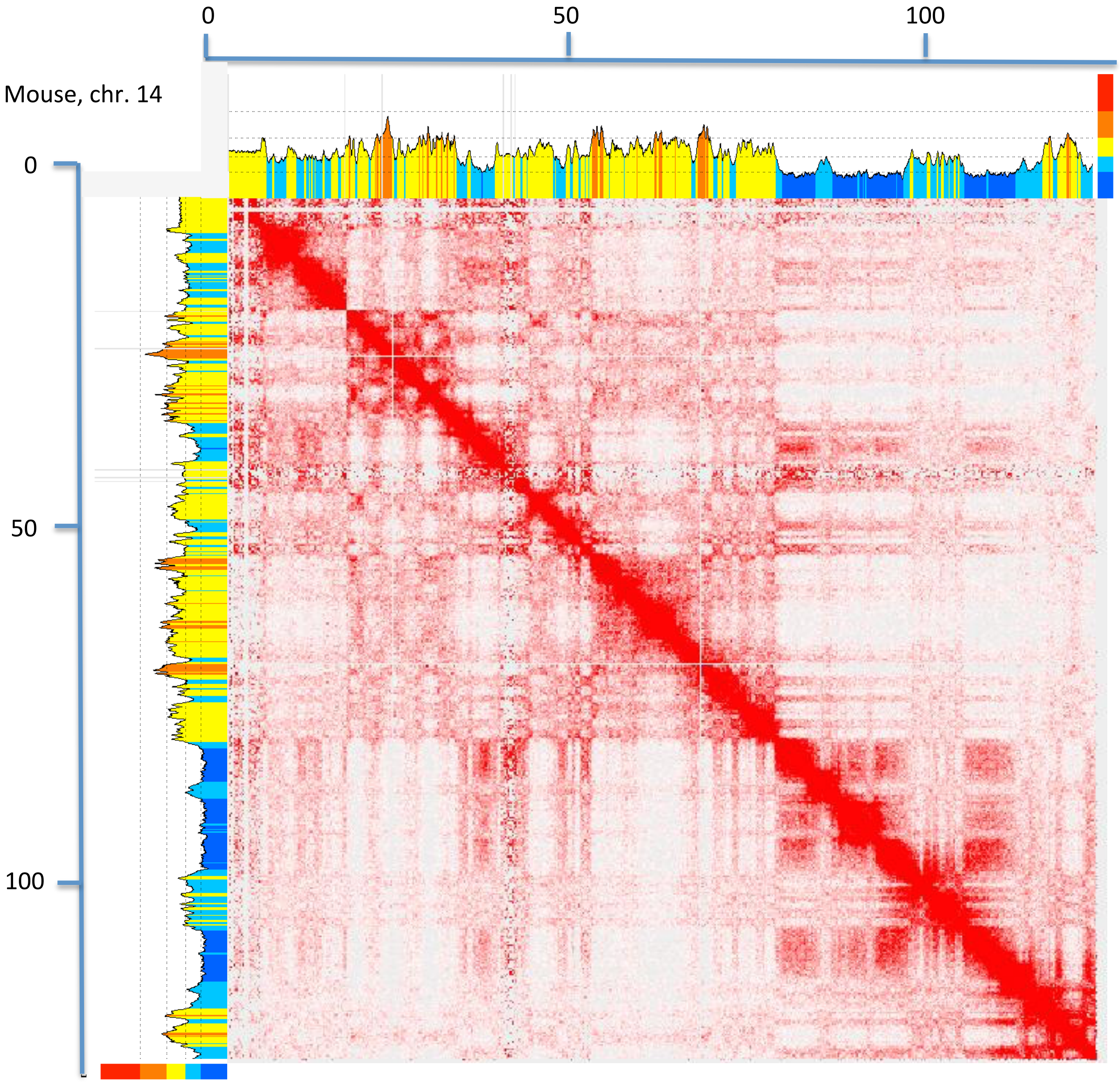

Fig. S18

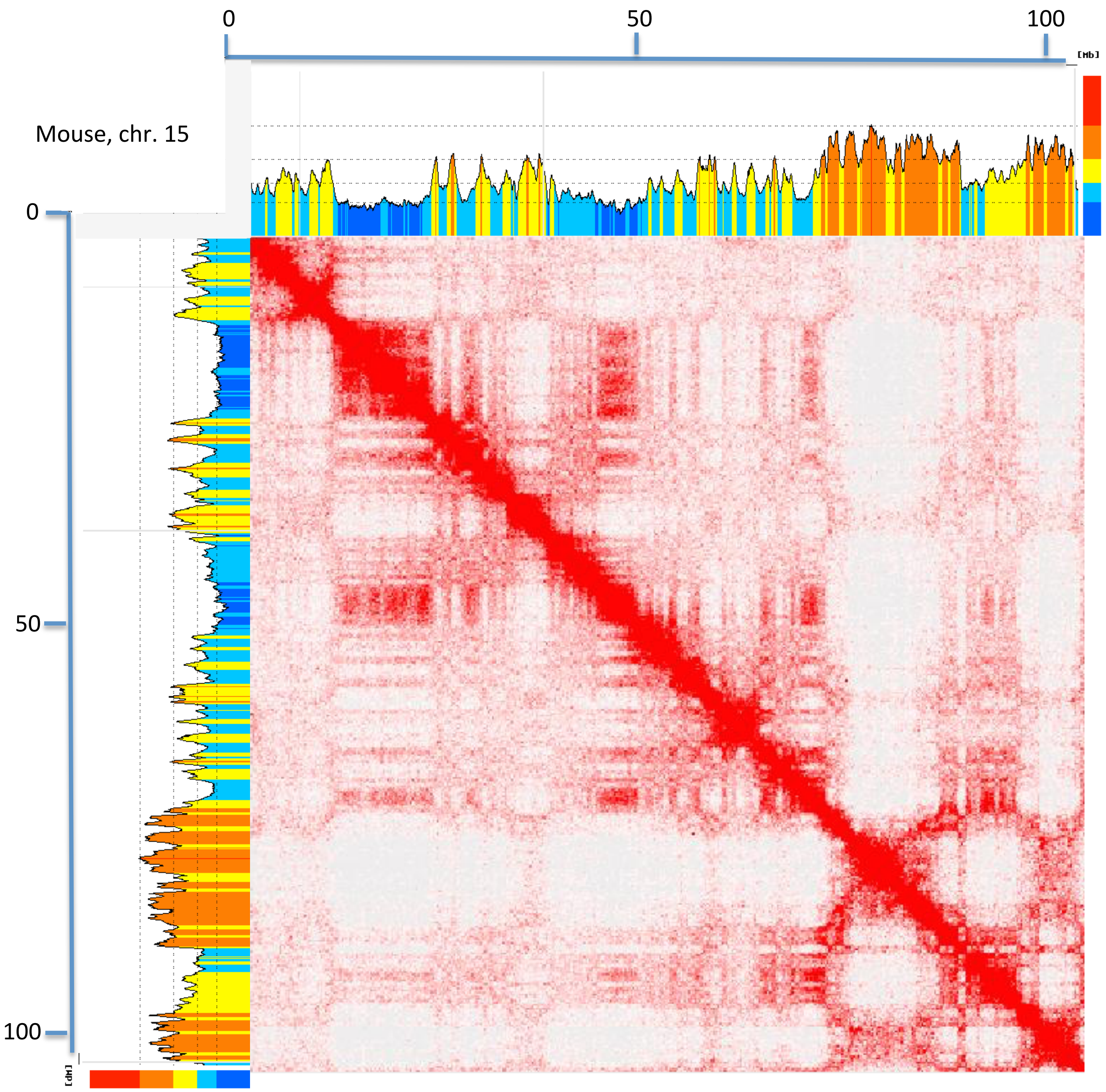

Fig. S19

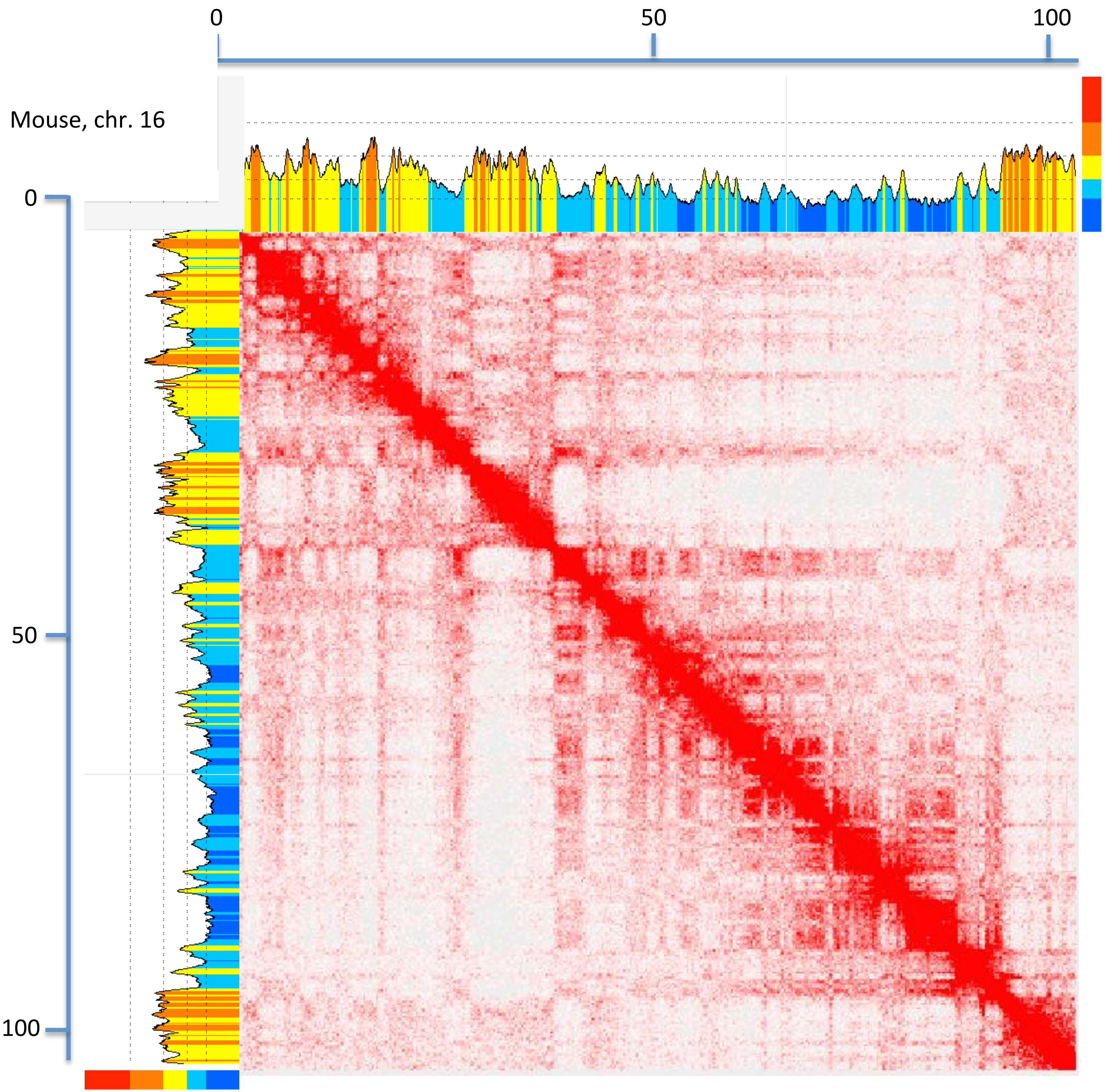

Fig. S20

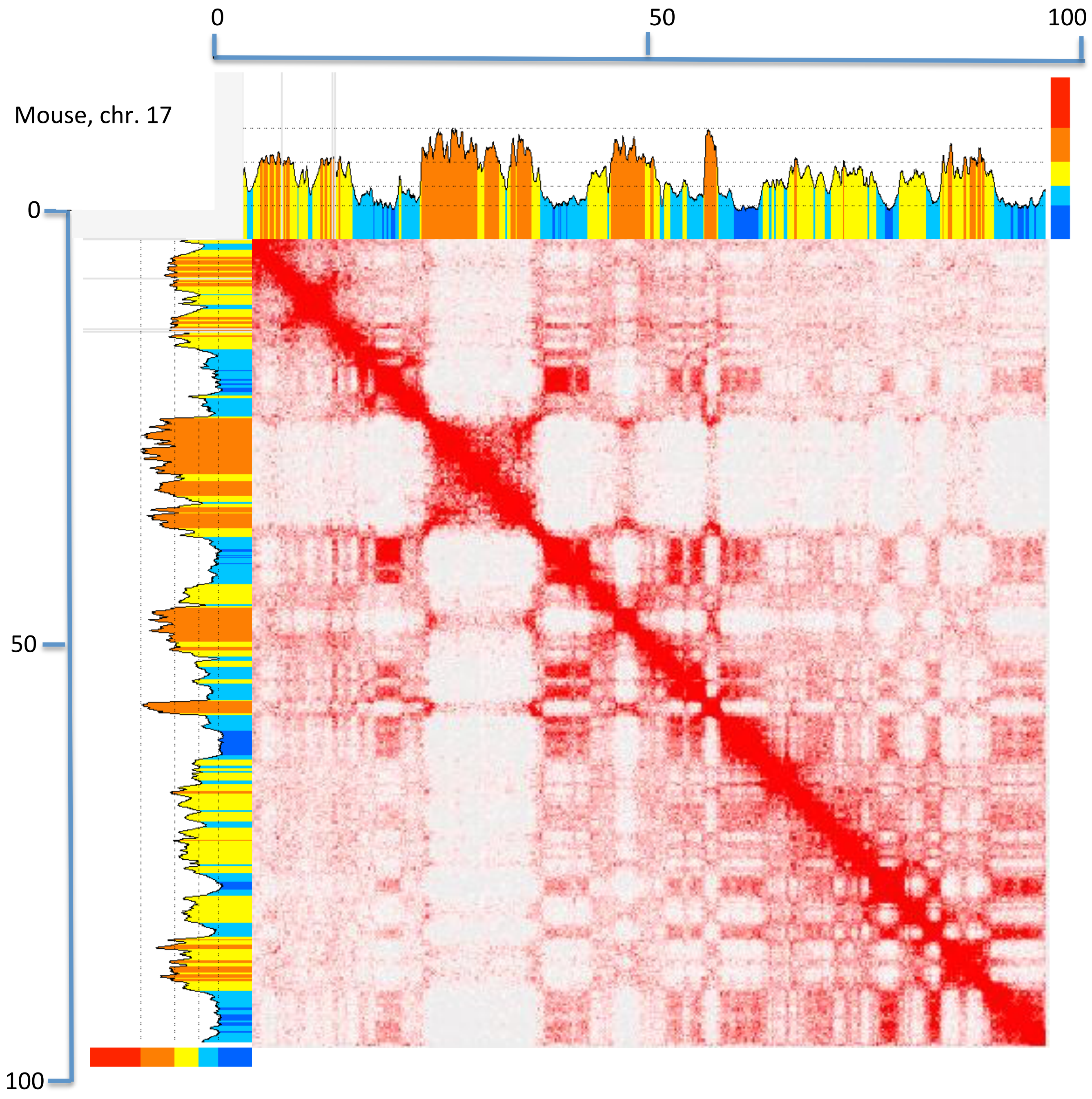

Fig. S21

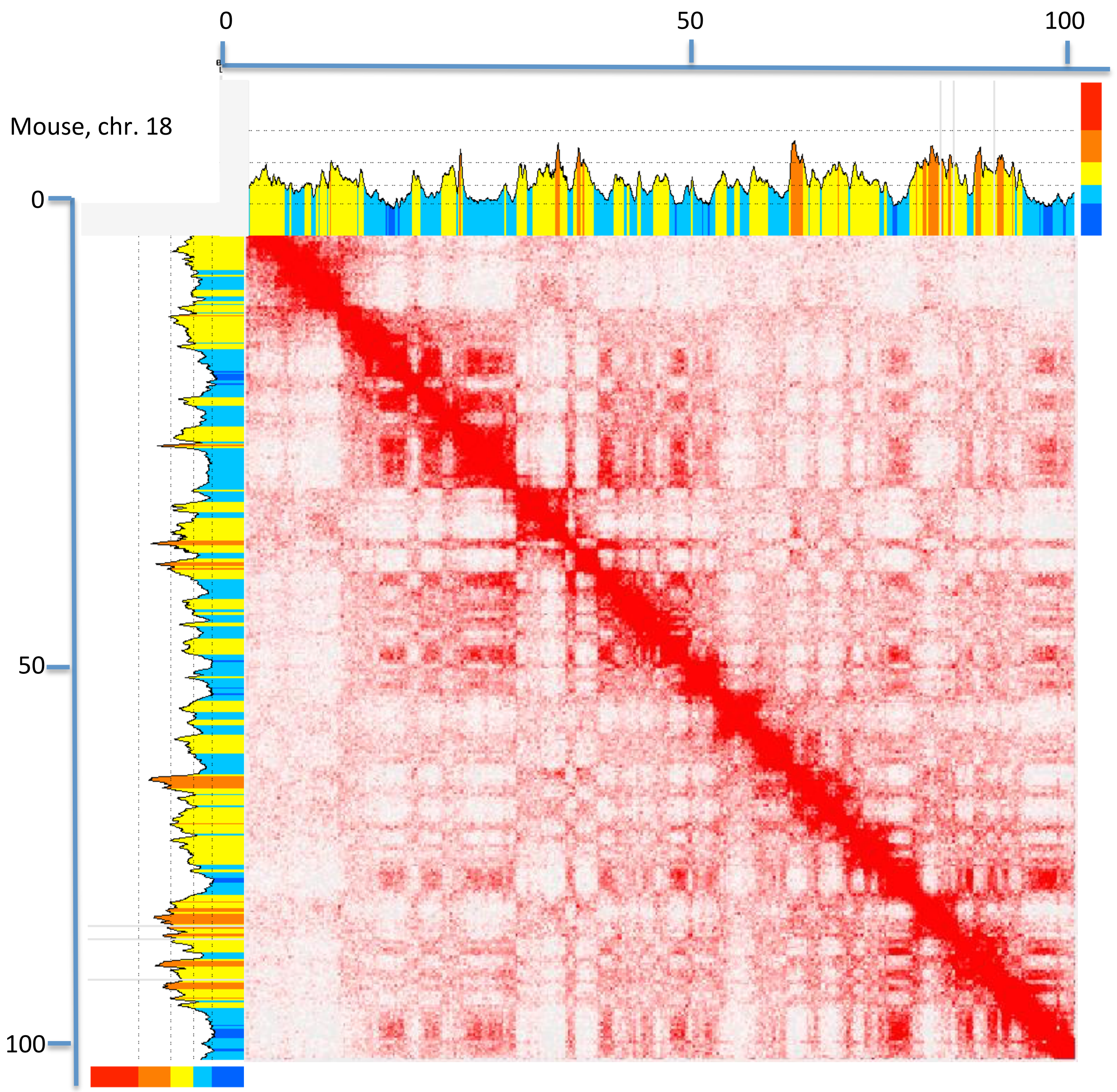

Fig. S22

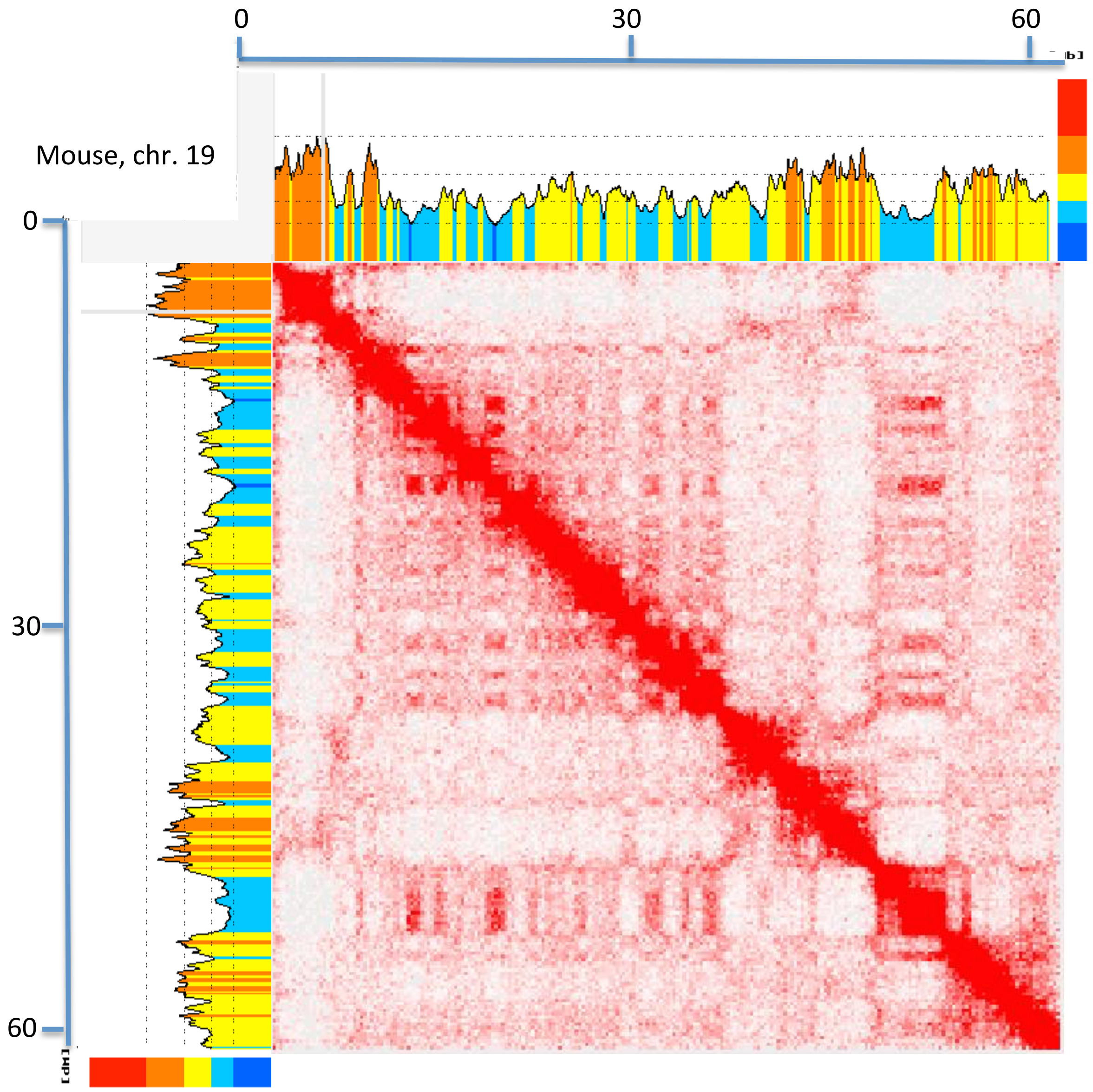

Fig. S23

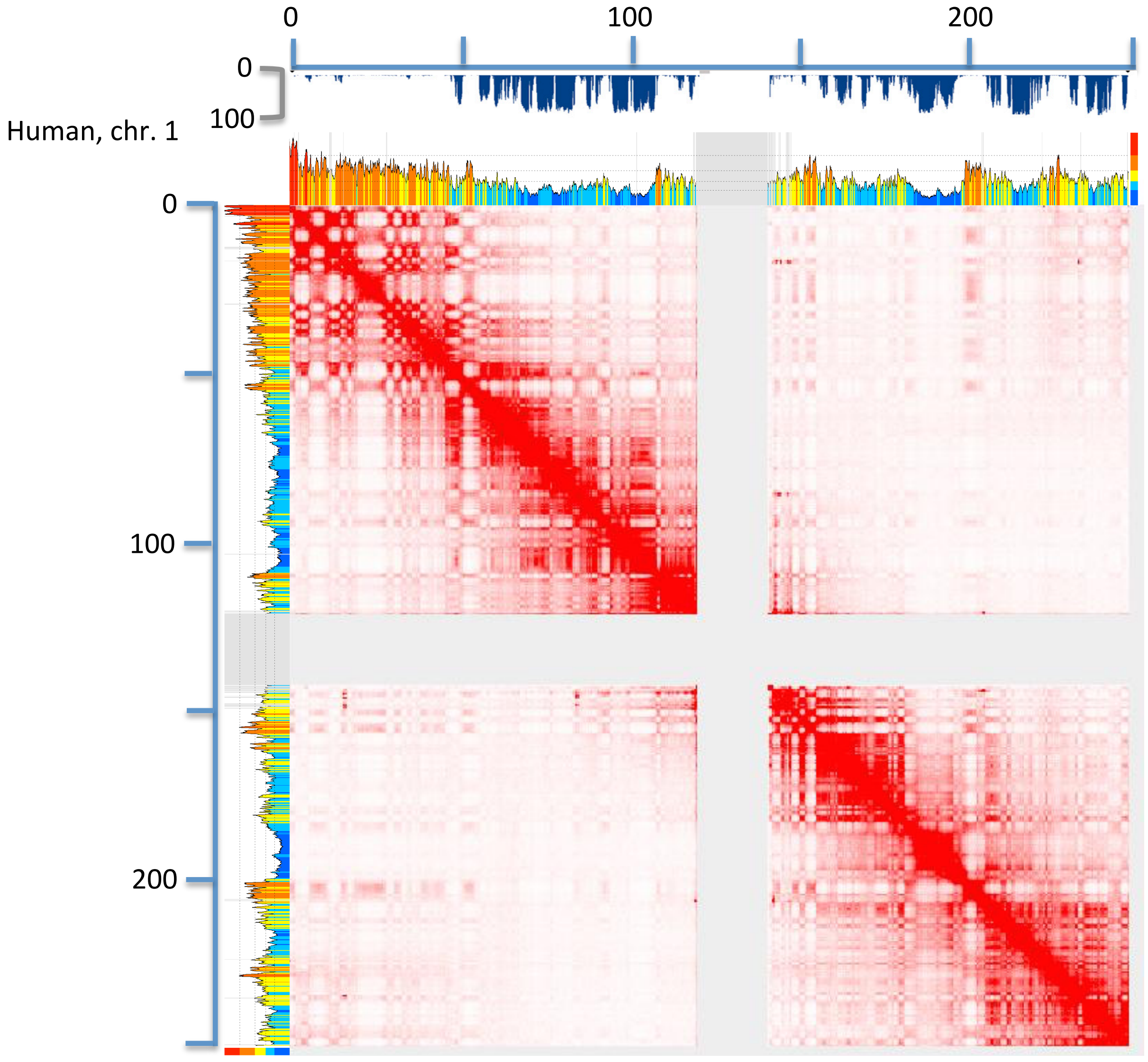

Fig. S24

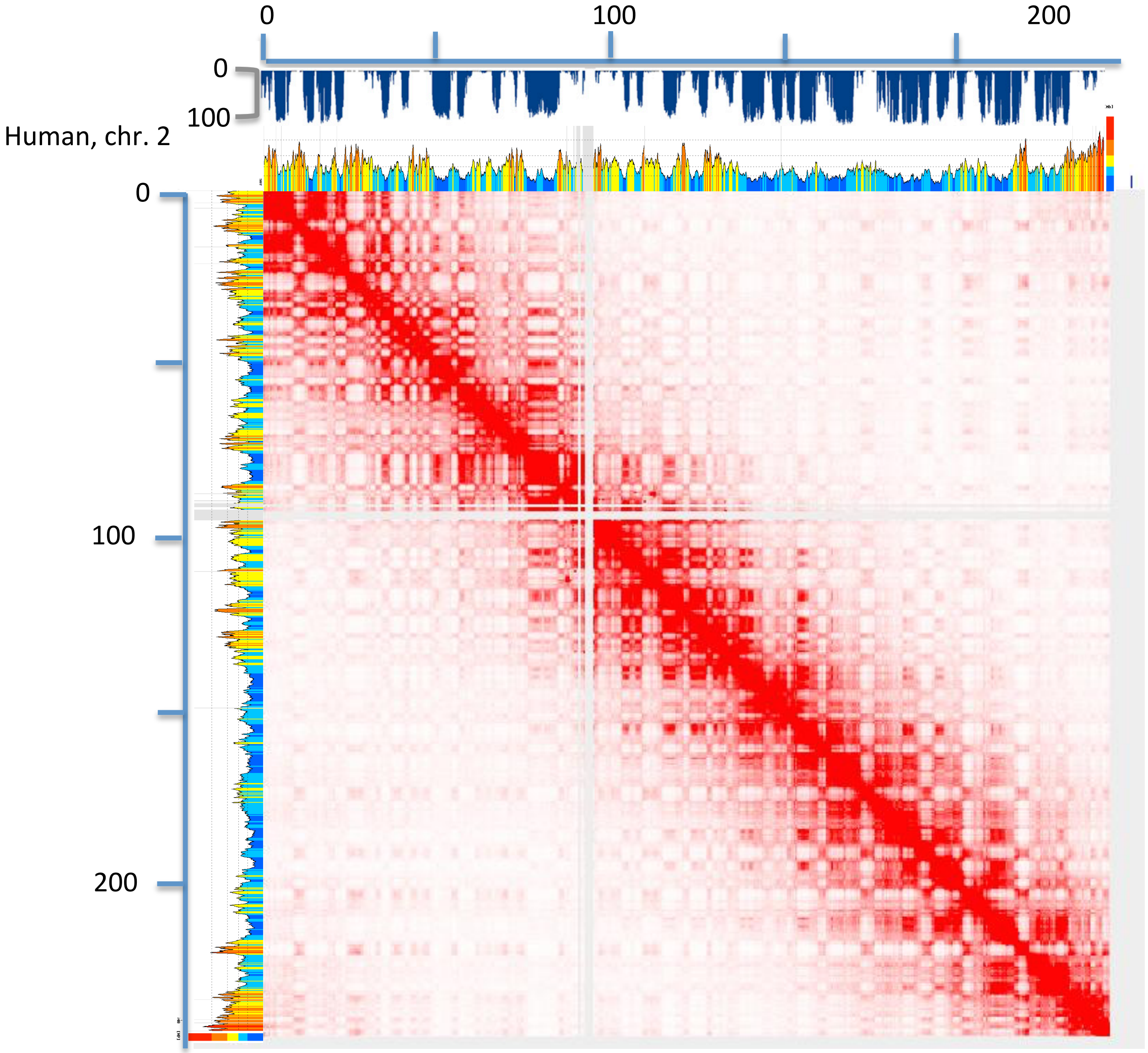

Fig. S25

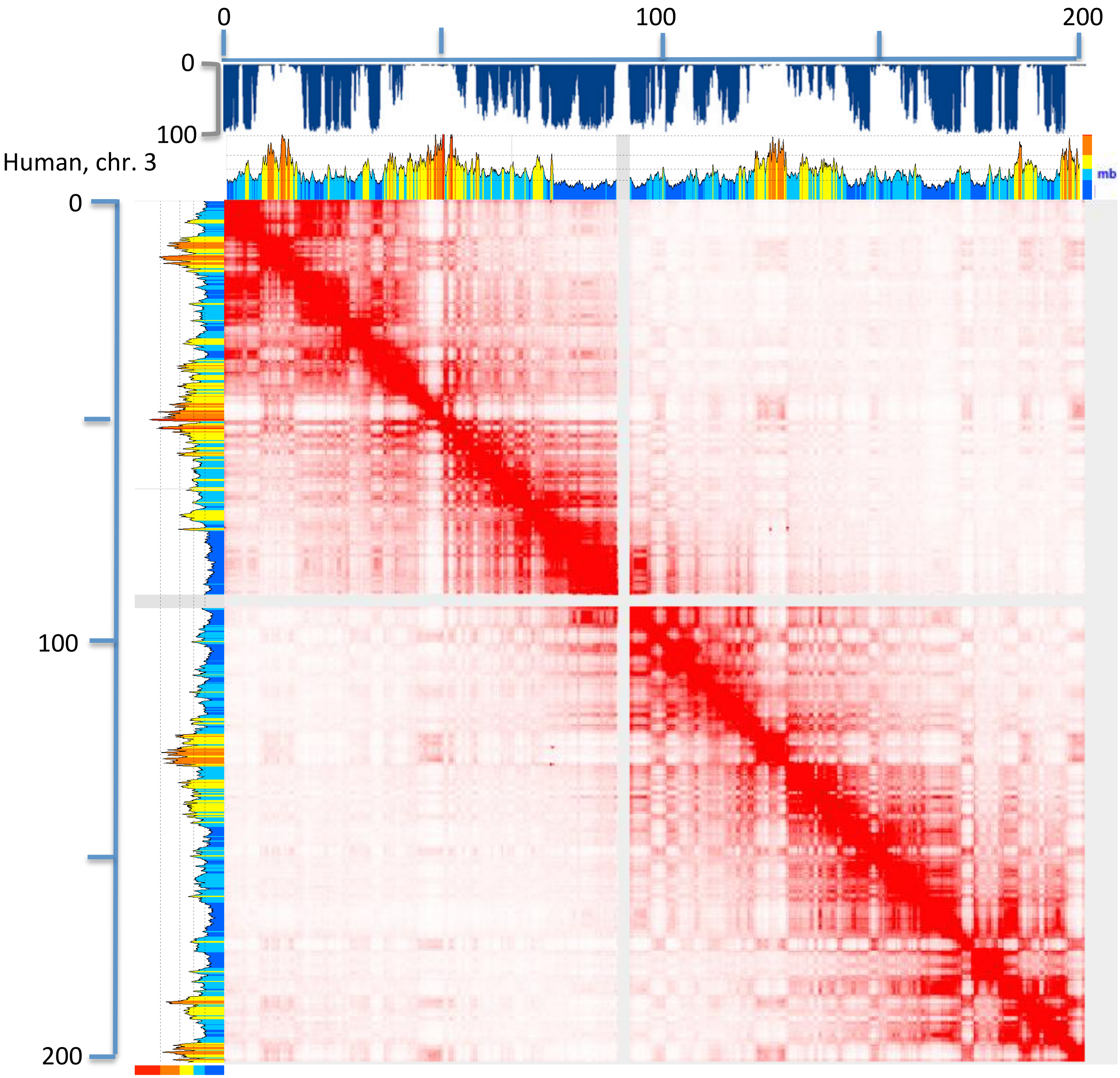

Fig. S26

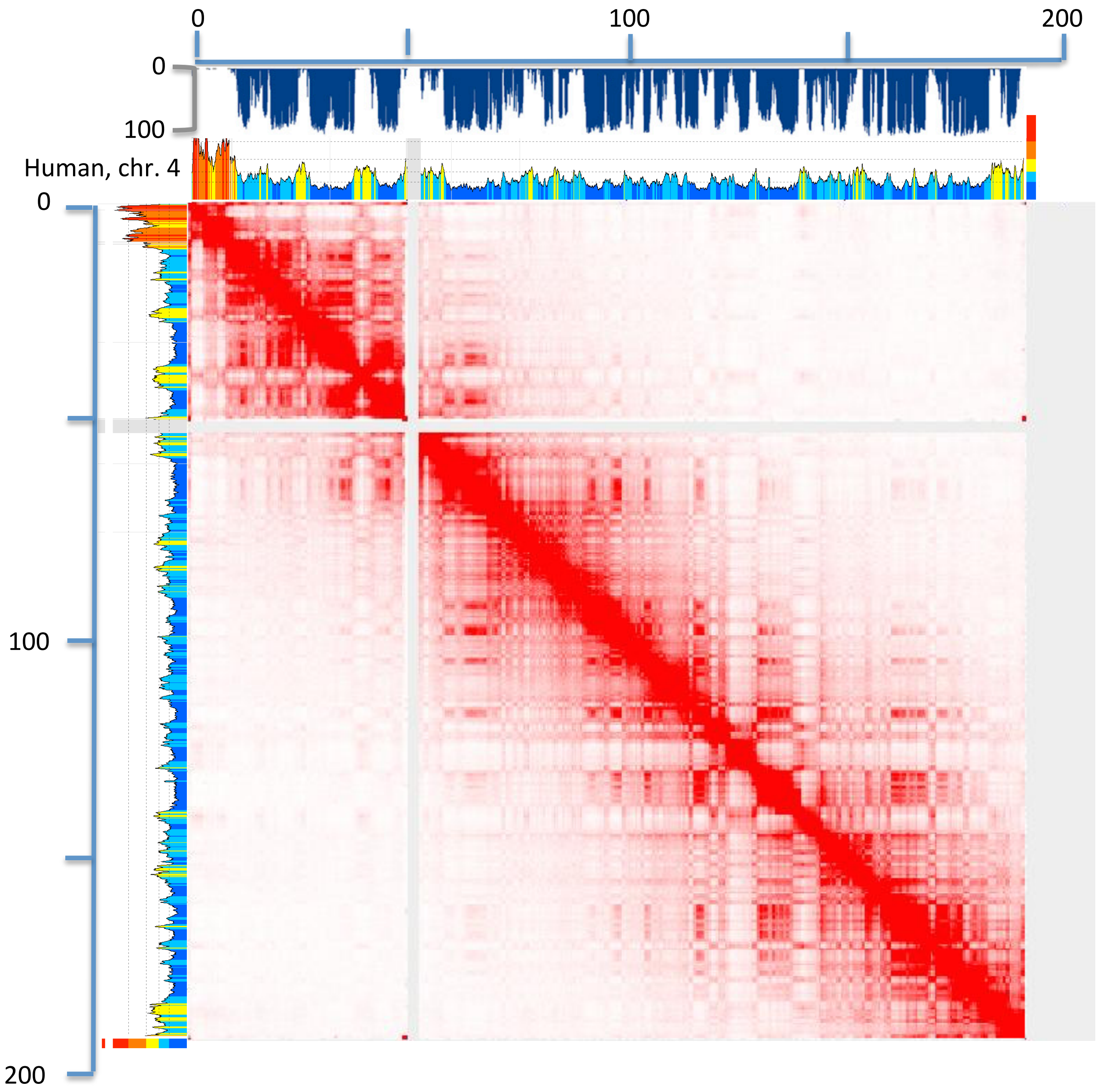

Fig. S27

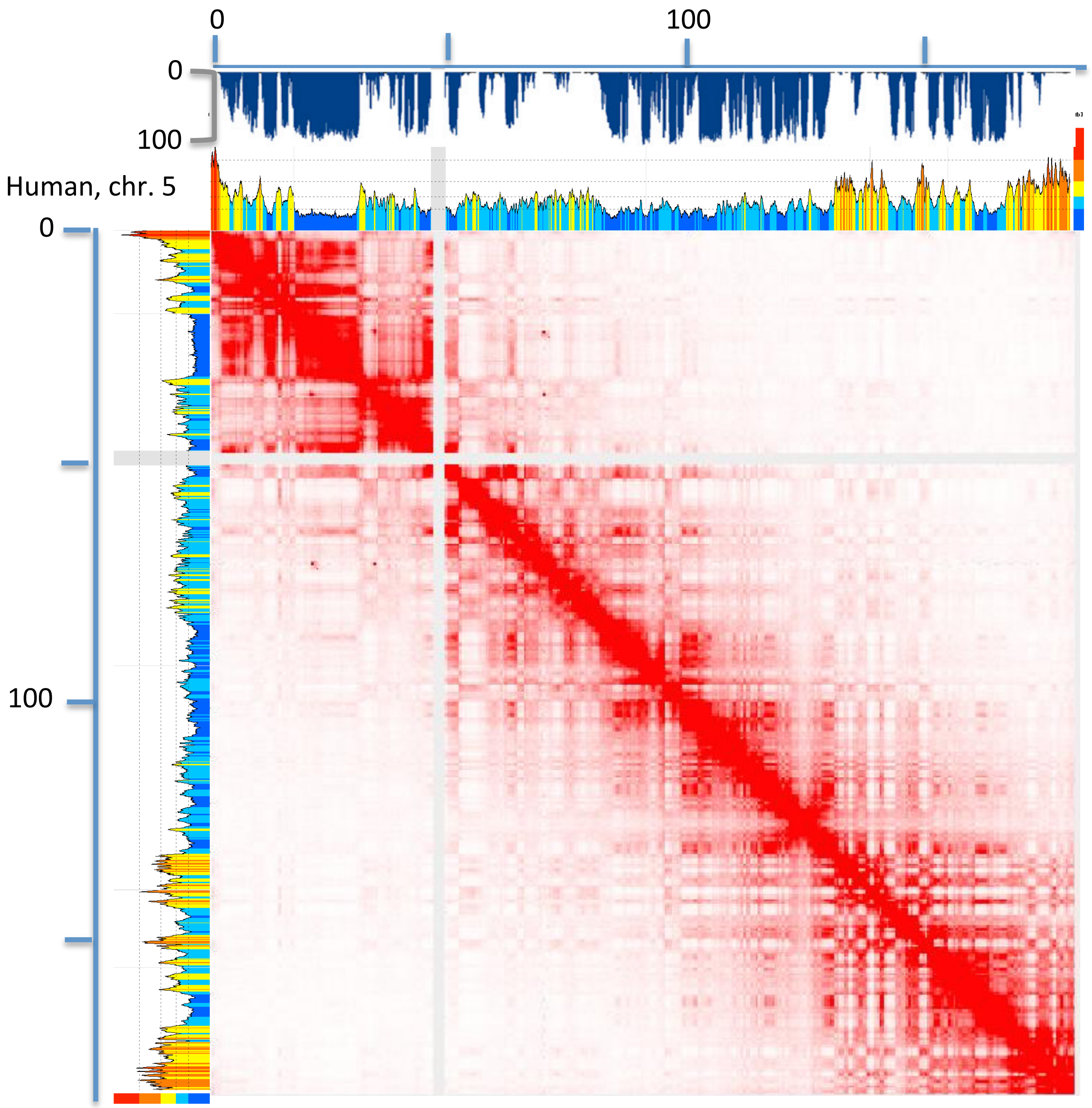

Fig. S28

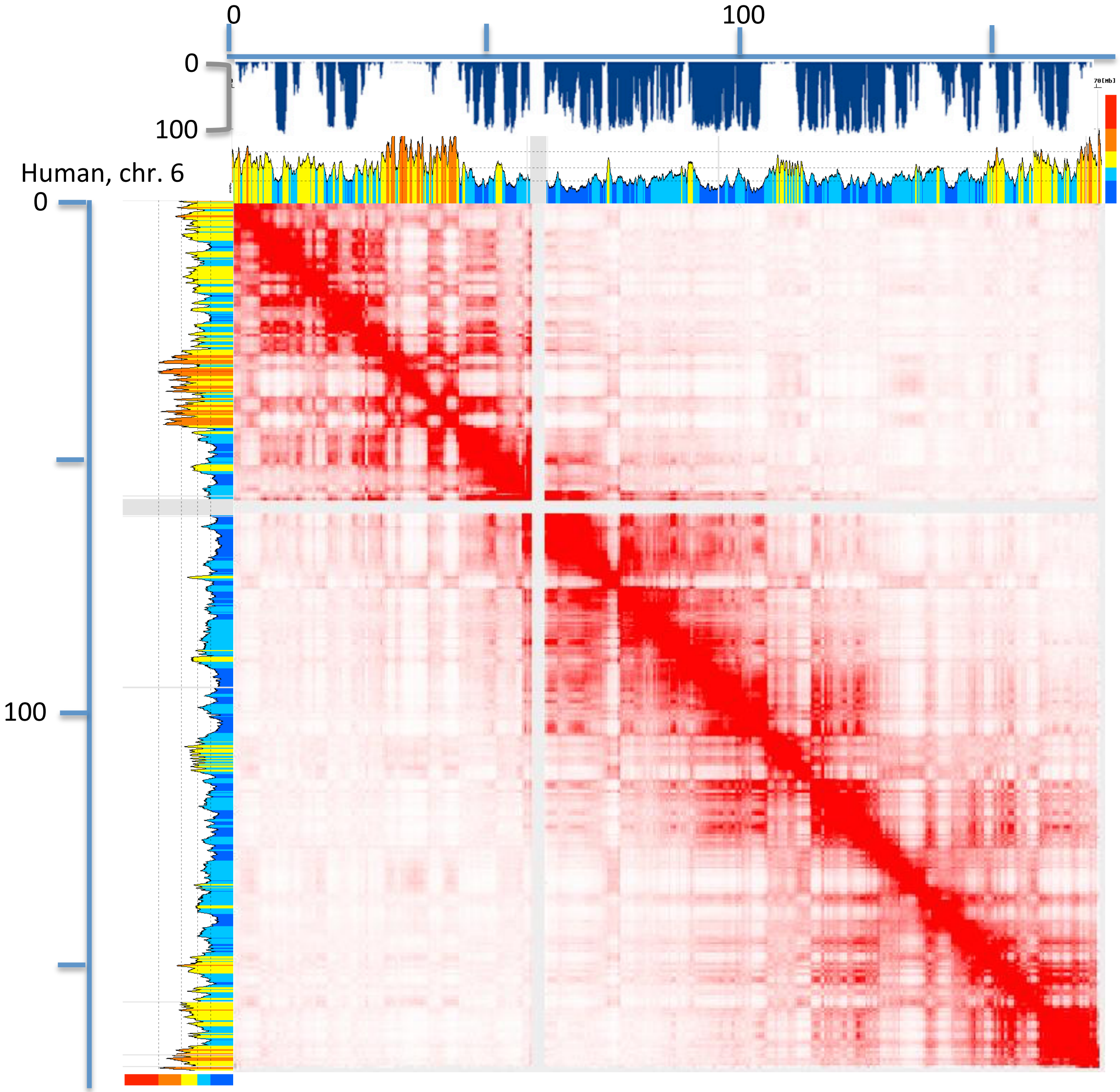

Fig. S29

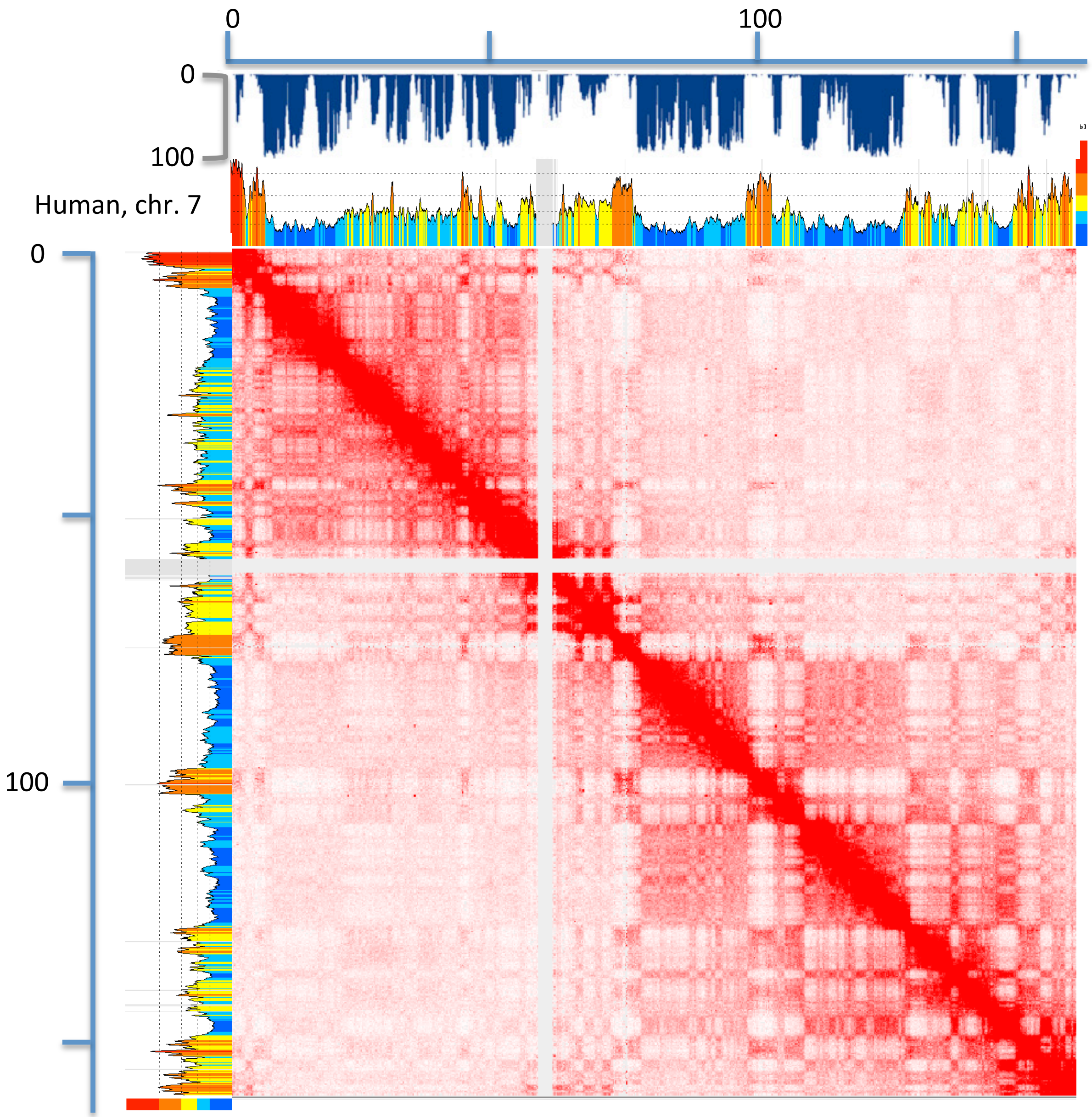

Fig. S30

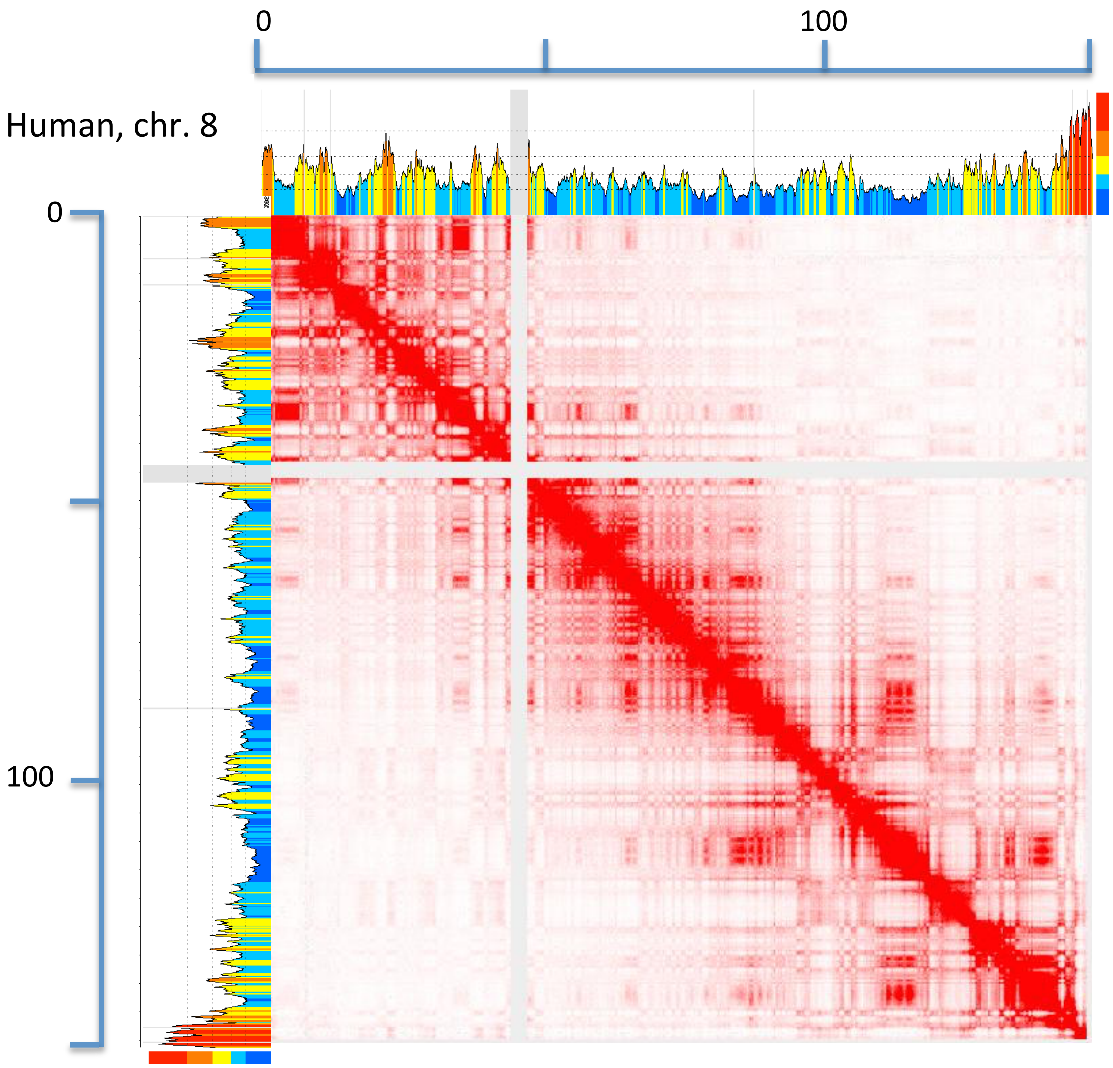

Fig. S31

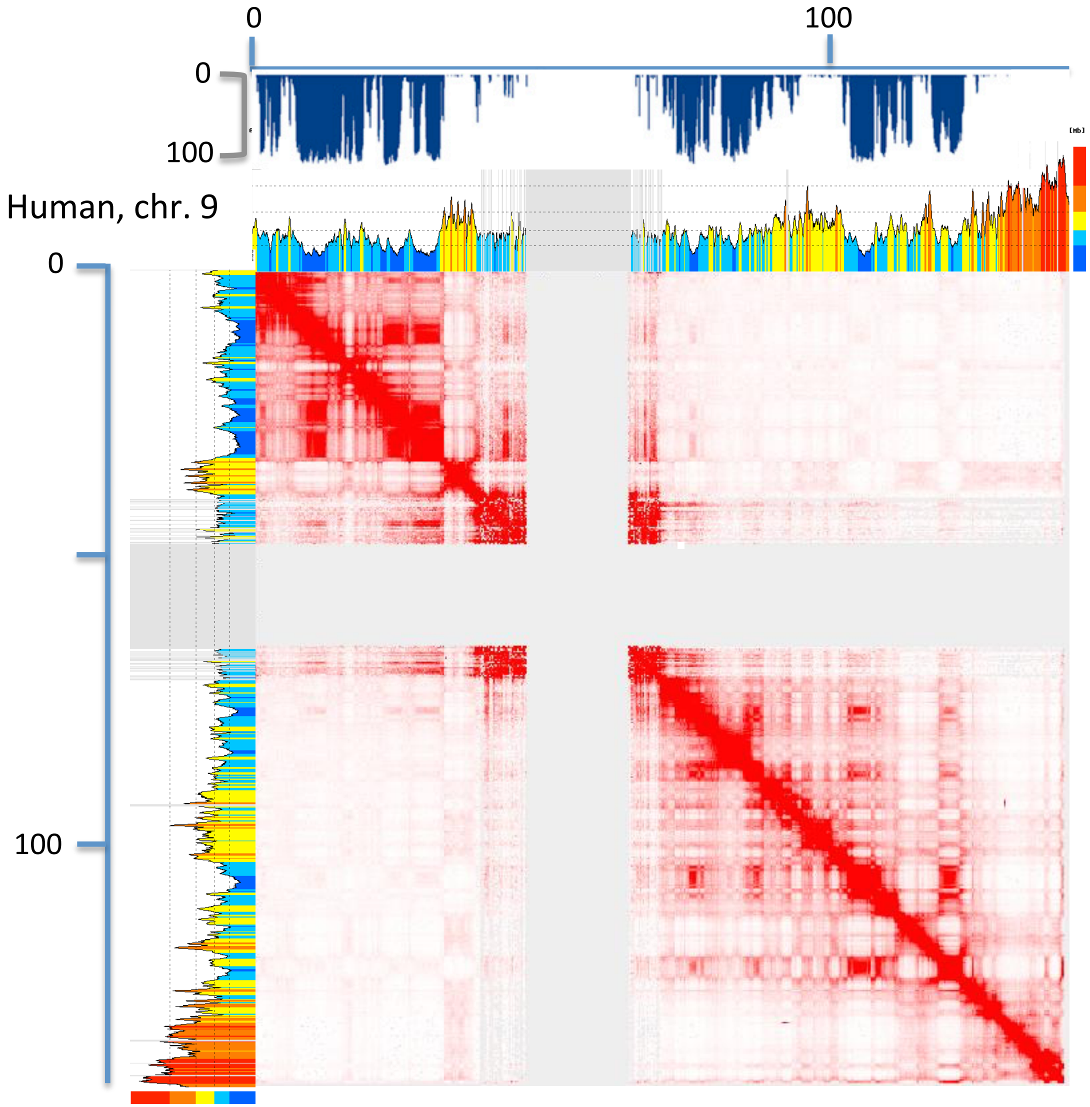

Fig. S32

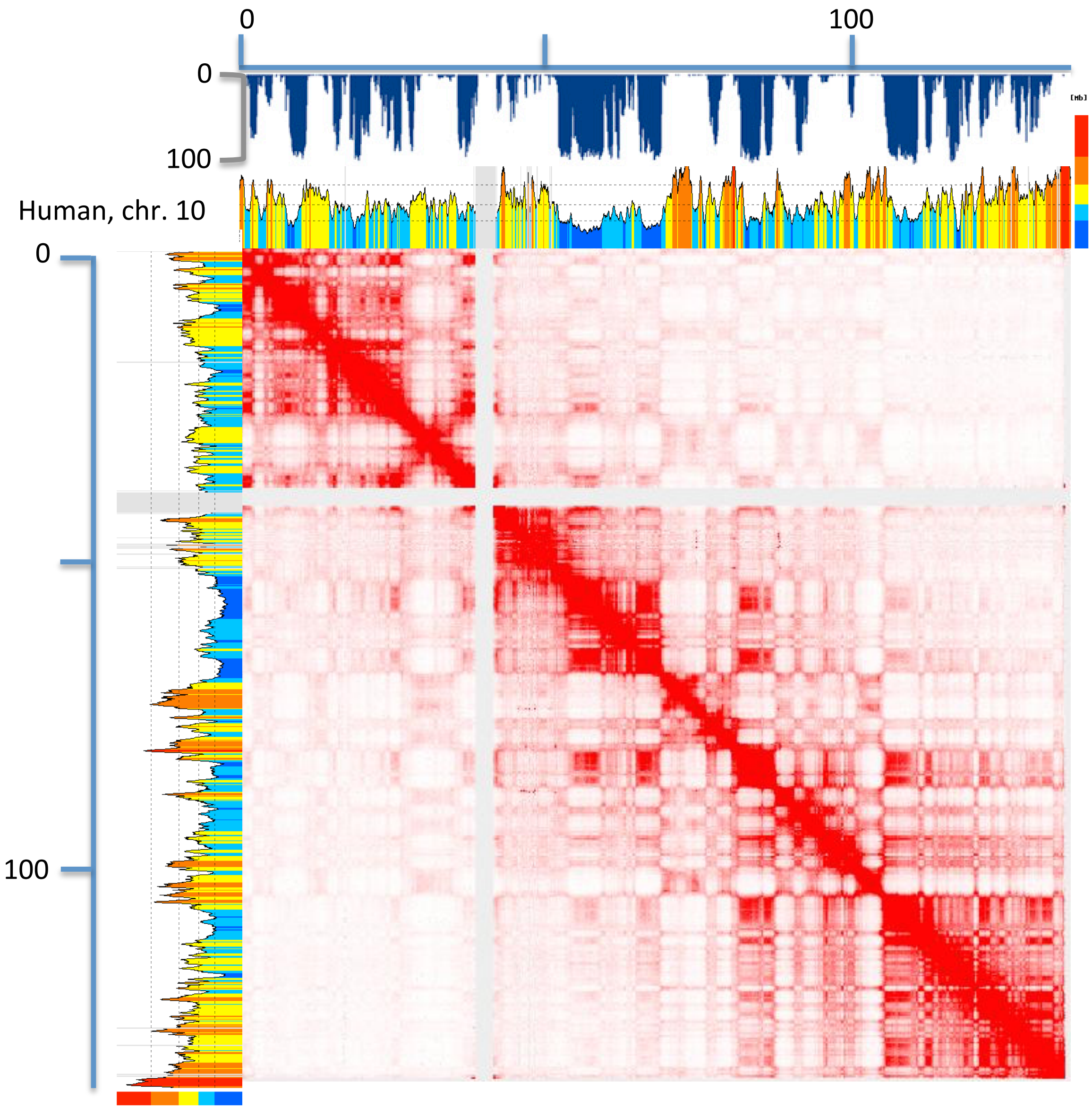

Fig. S33

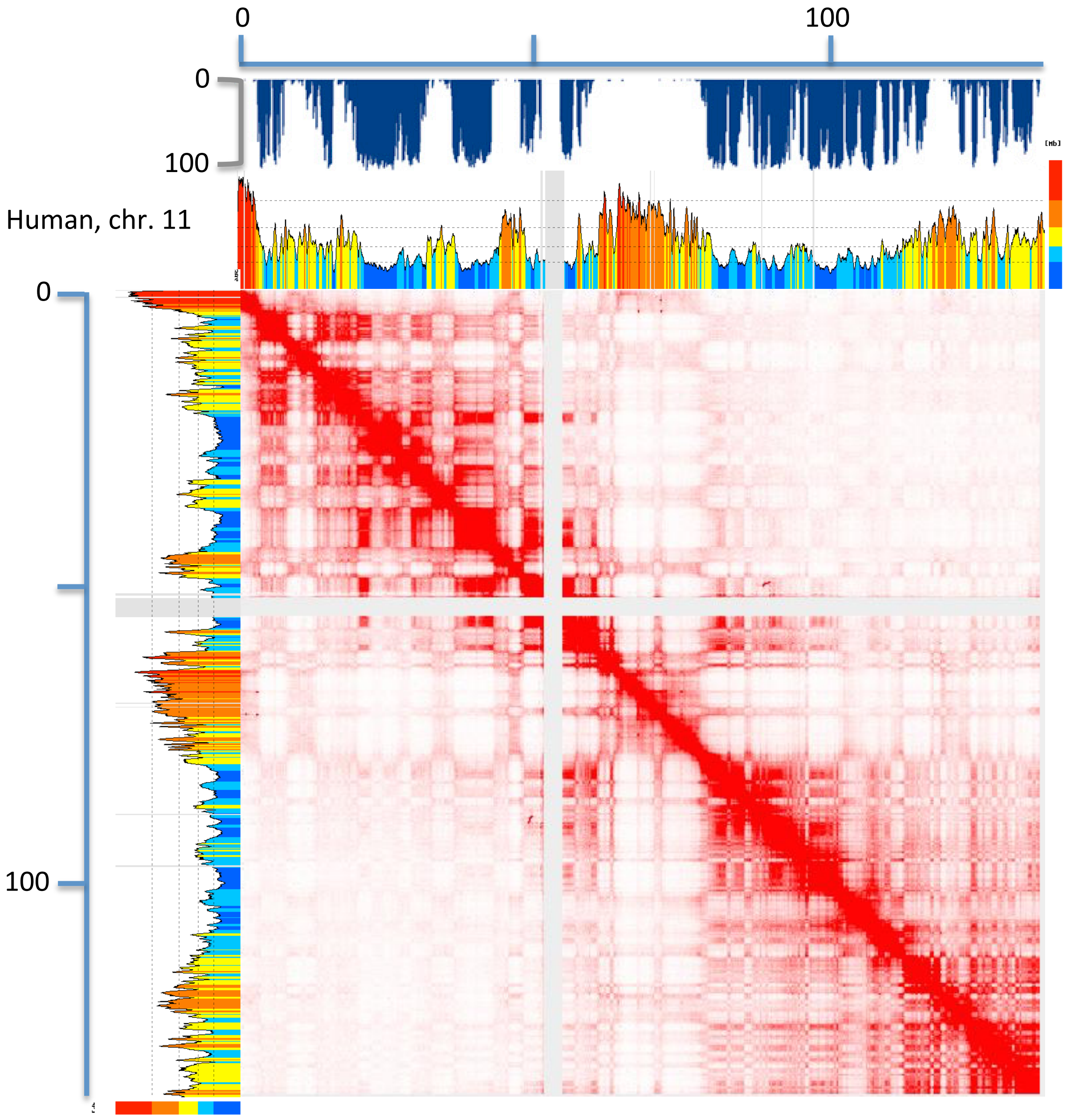

Fig. S34

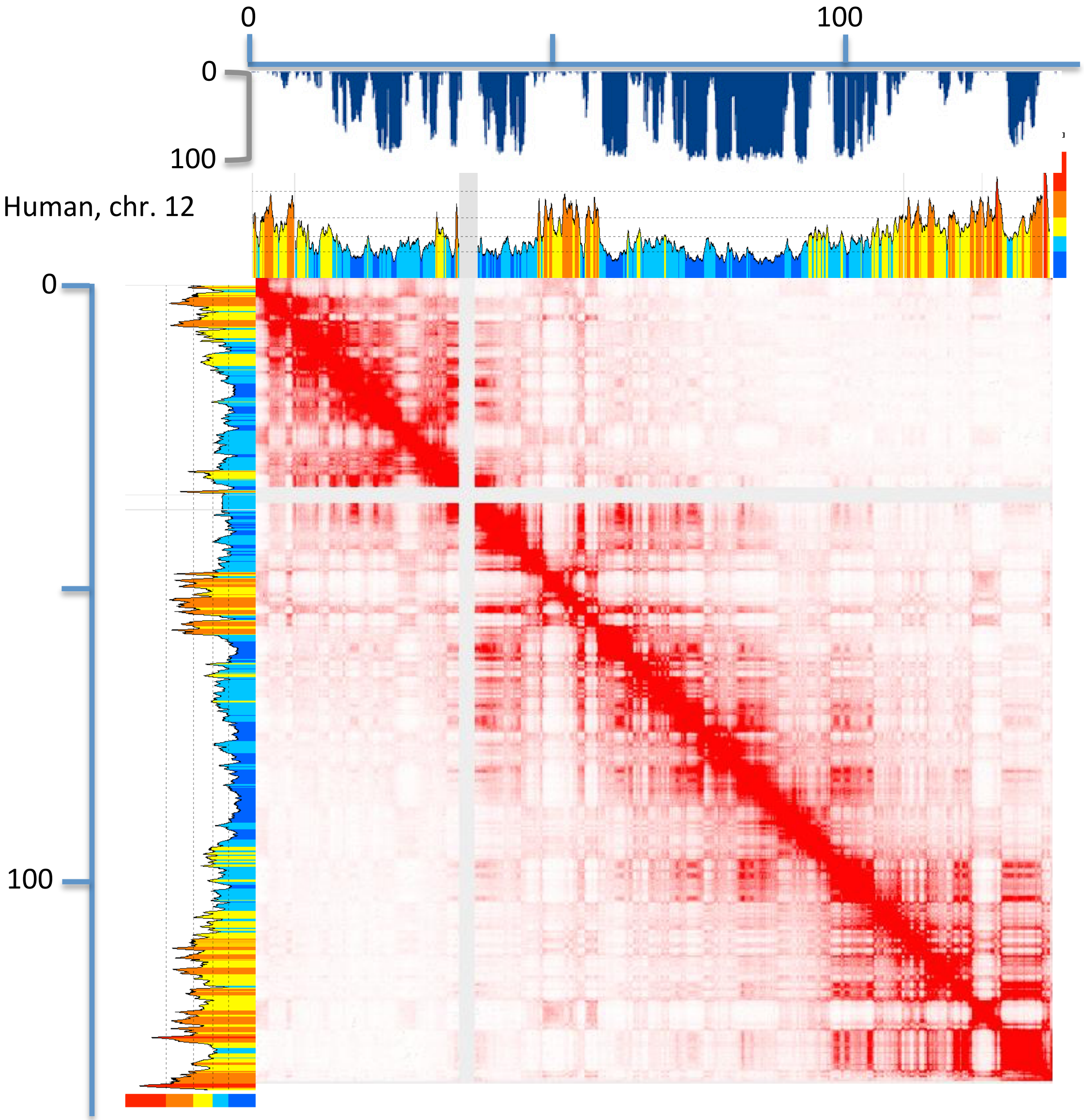

Fig. S35

Fig. S36

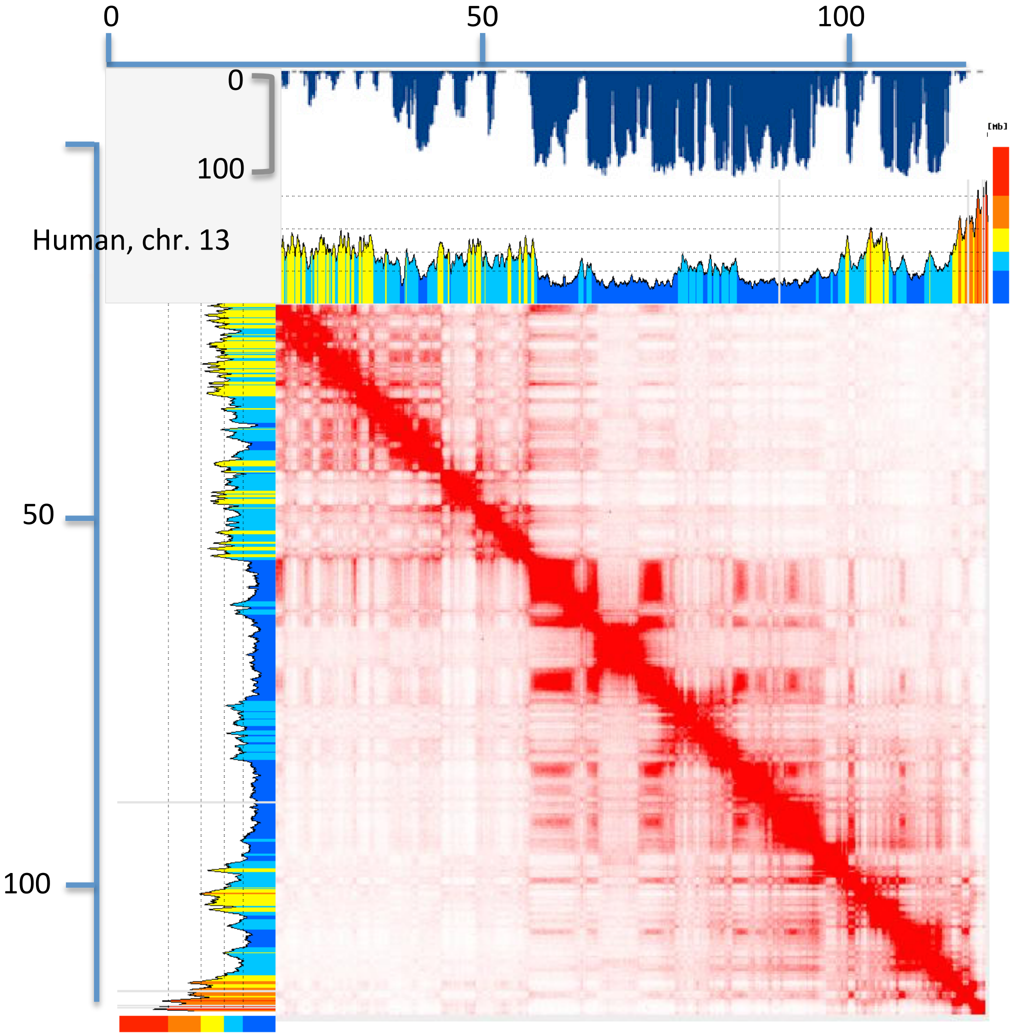

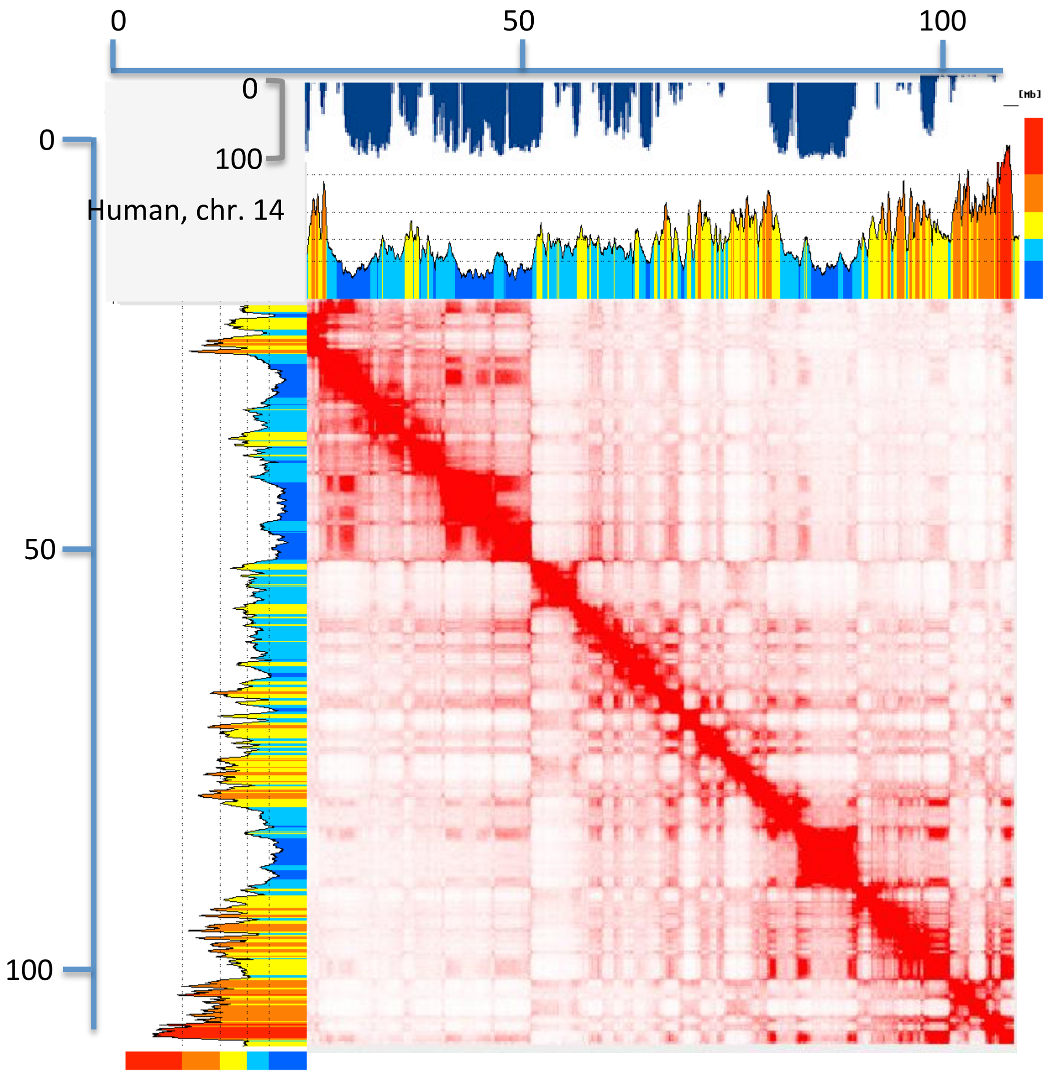

Fig. S37

Fig. S38

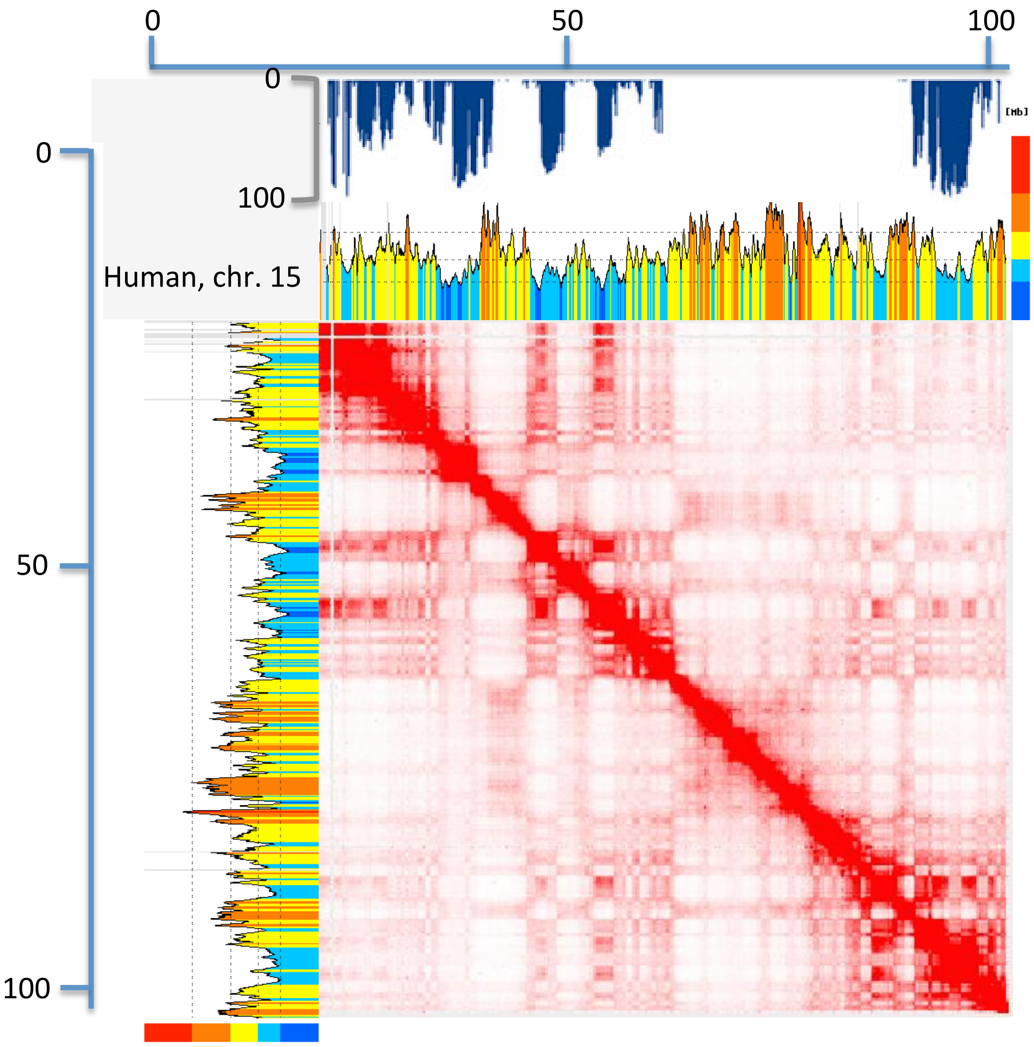

Fig. S39

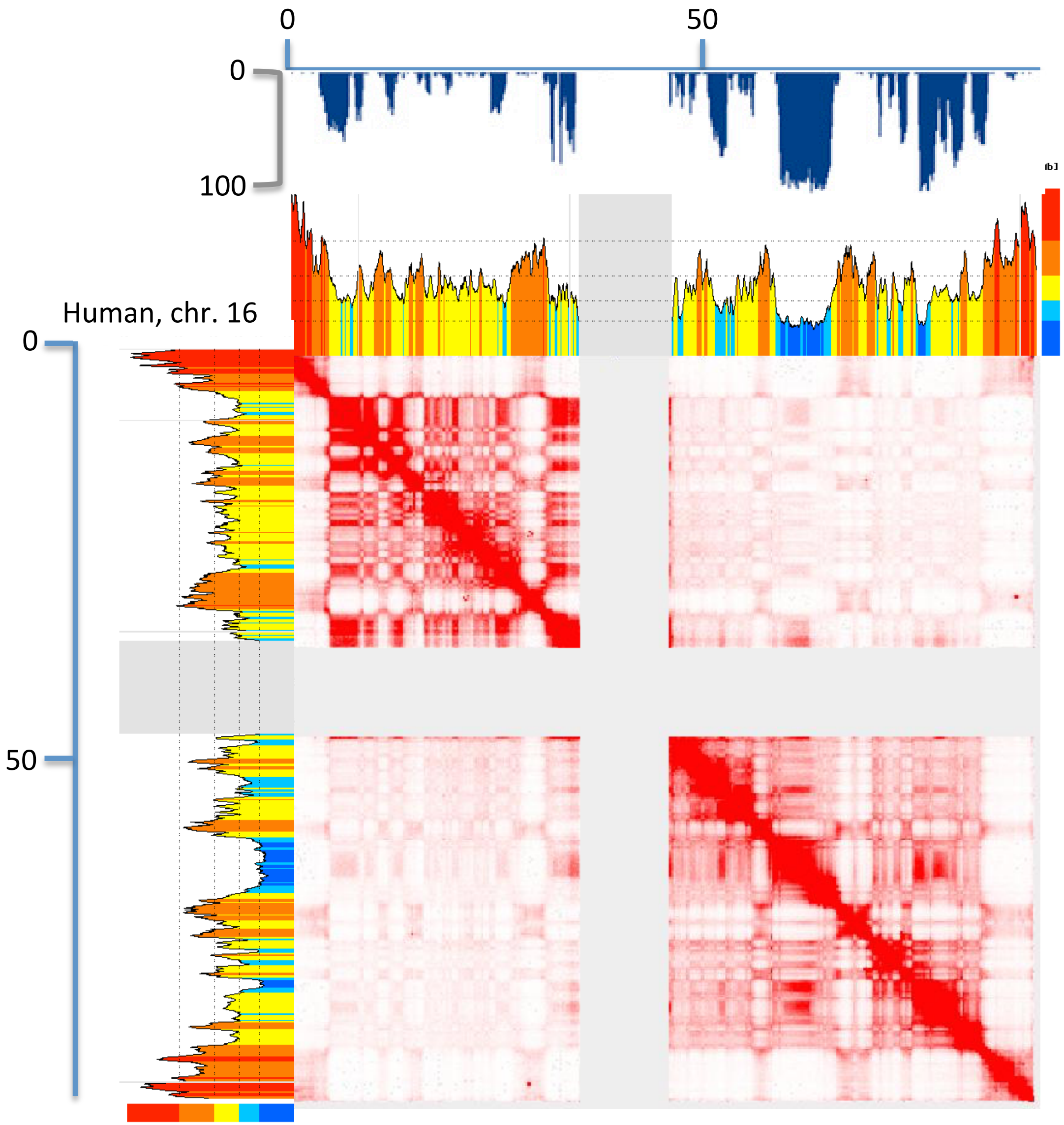

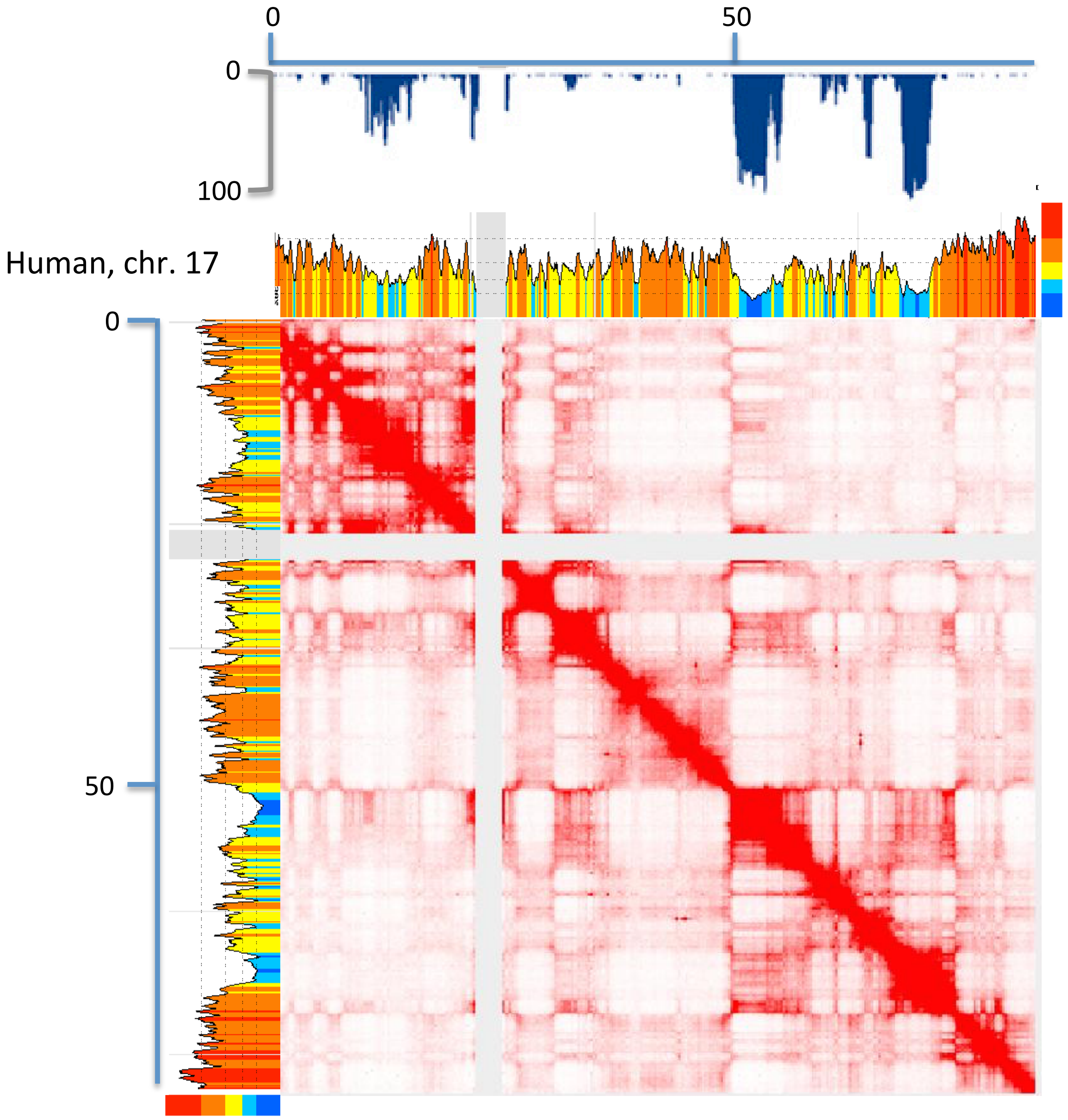

Fig. S40

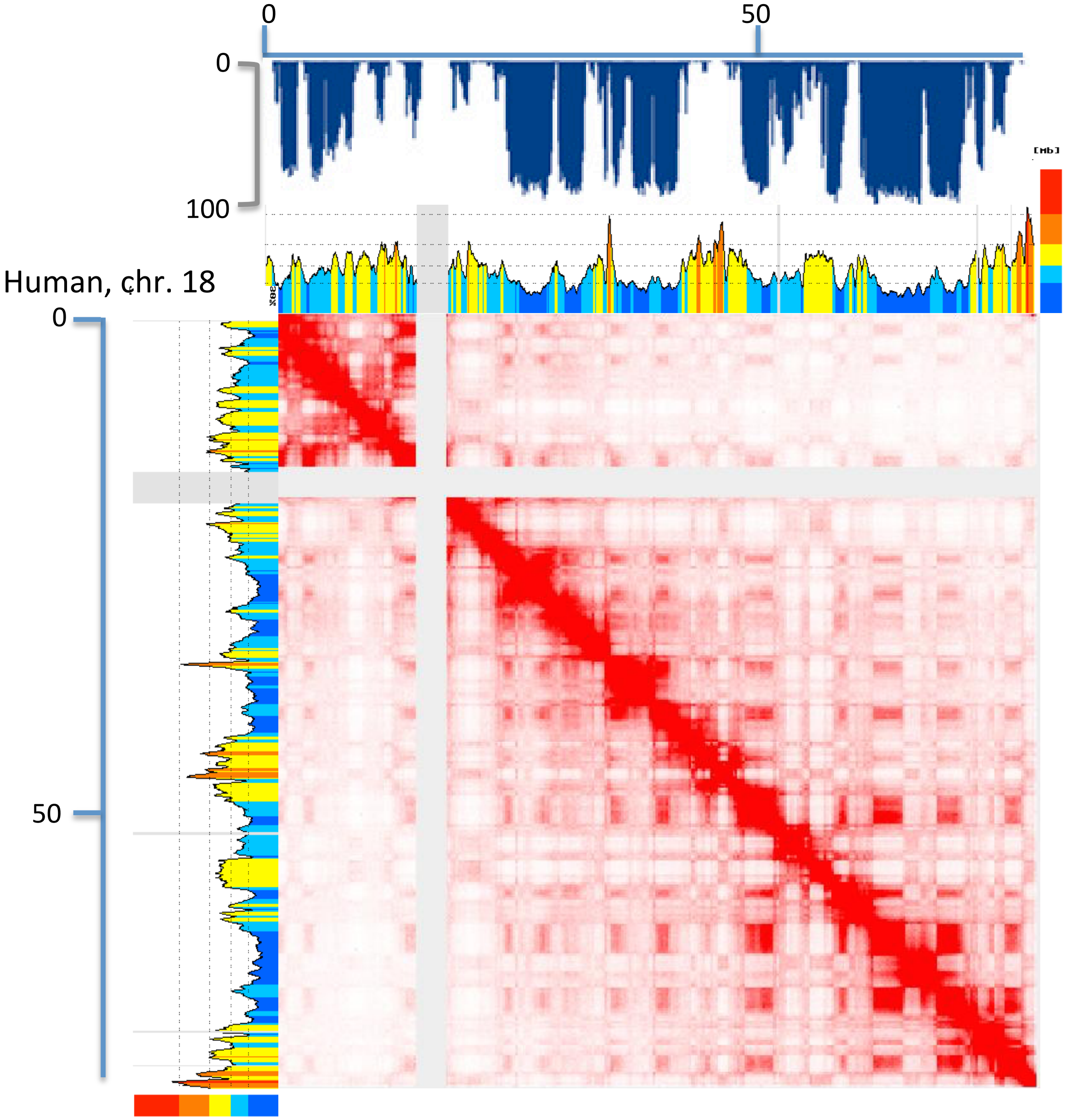

Fig. S41

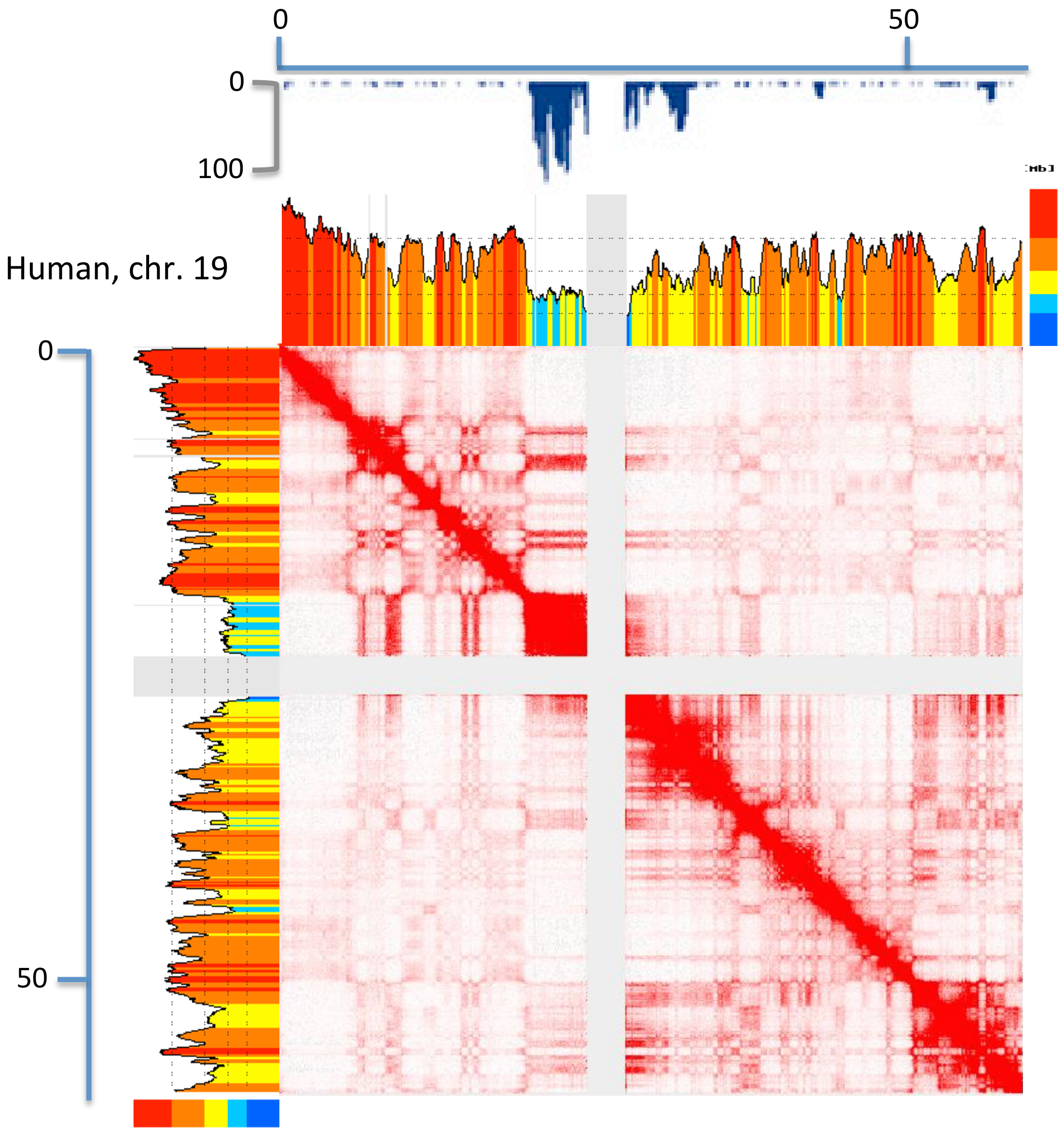

Fig. S42

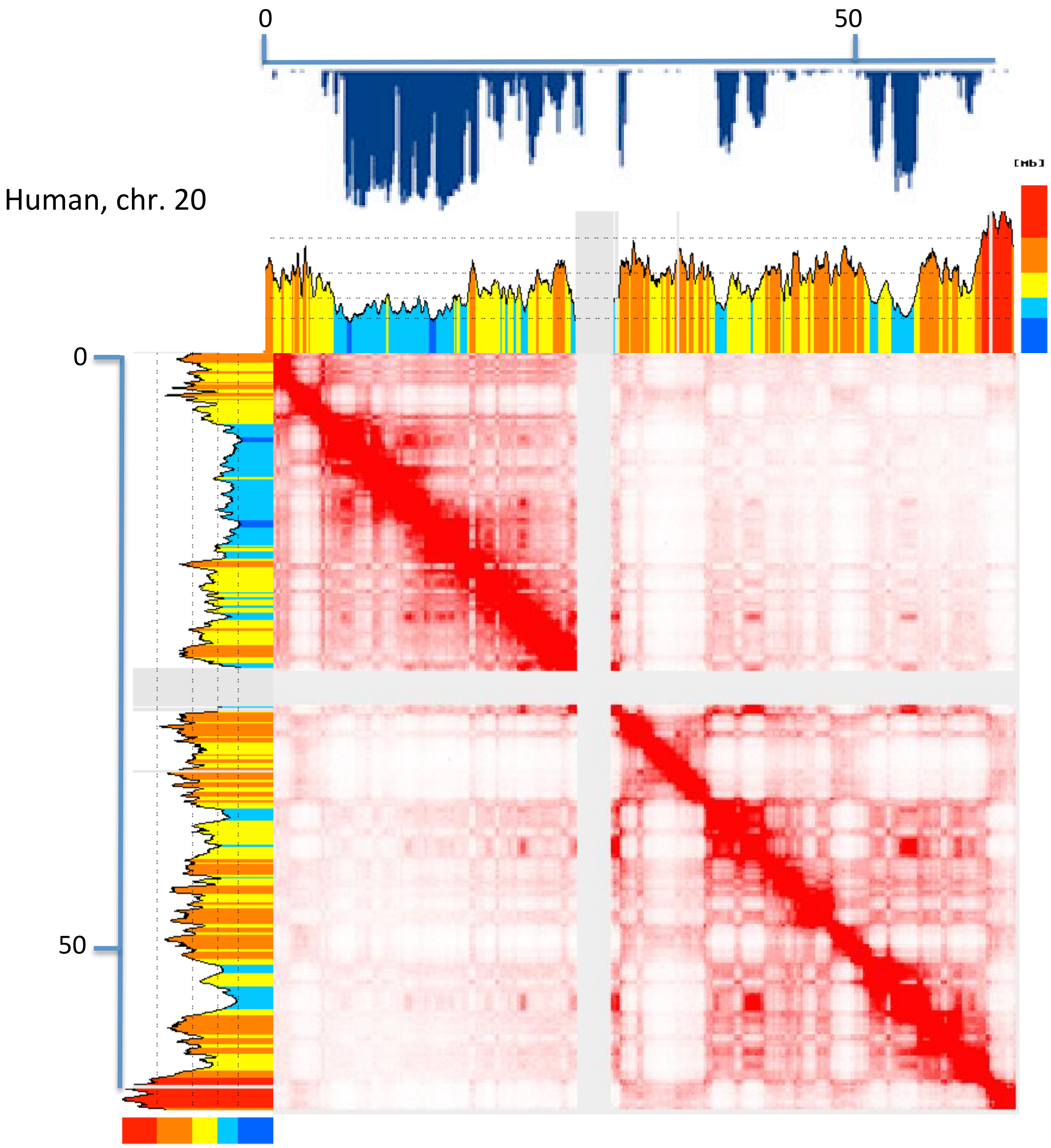

Fig. S43

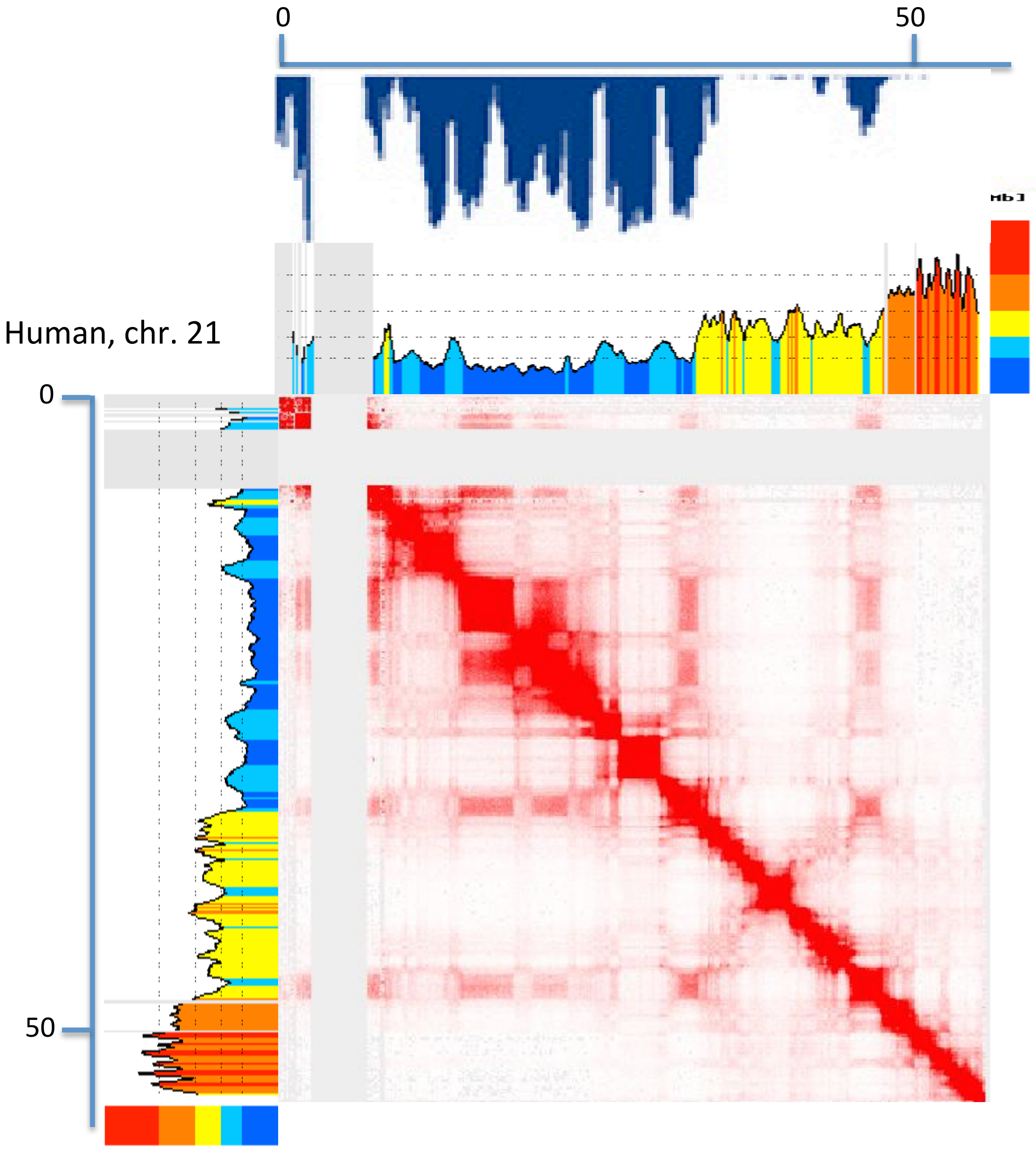

Fig. S44

Fig. S45

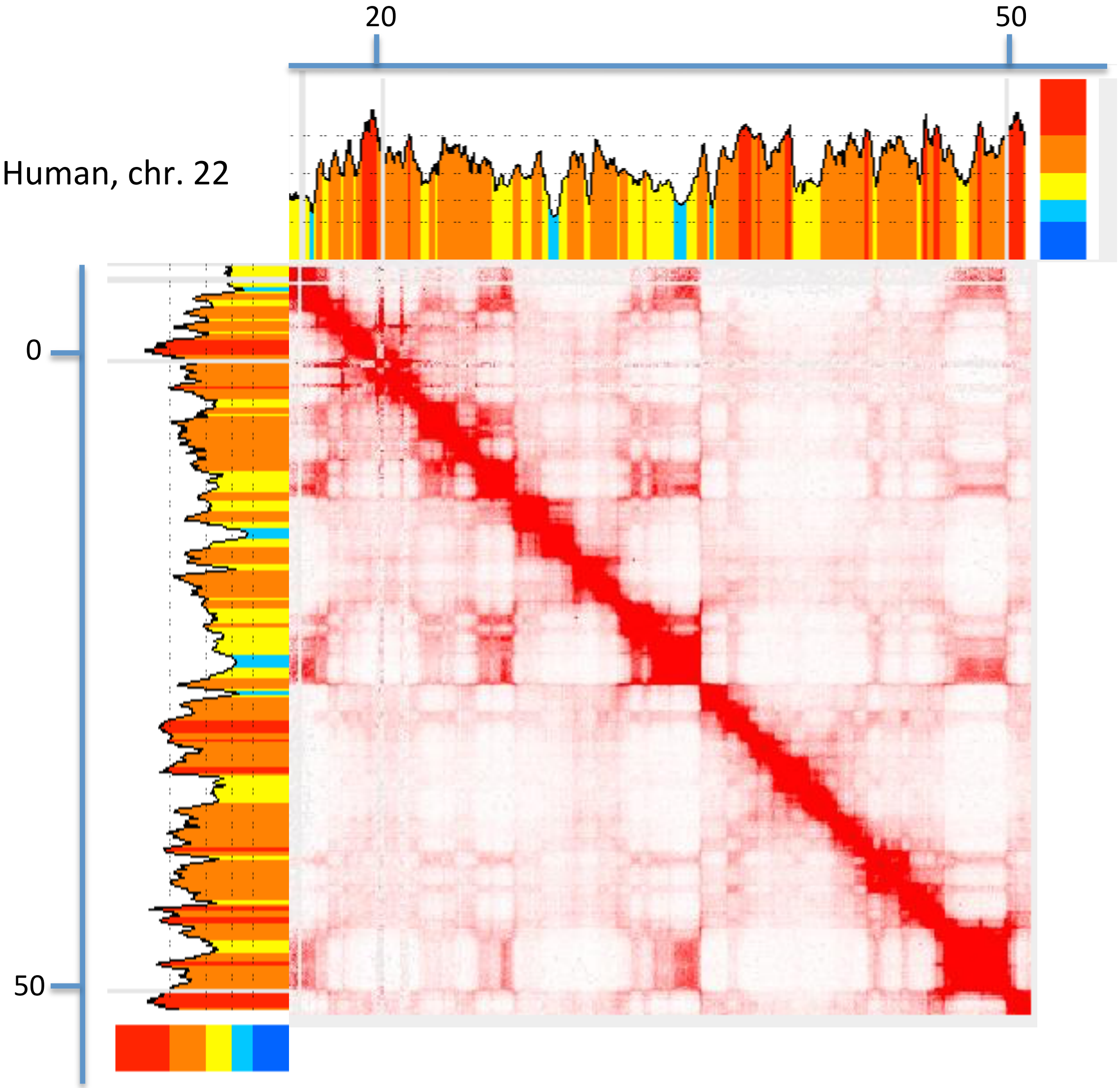

Supplement: S2 File — Fig S1. Association analysis of genomic regions based on permutation test: (A) TADs from human IMR90 fibroblast overlap significantly with TADs from human hESC cells. (B) Human isochores overlap significantly with TADs from human cells. (C) Human isochores overlap significantly with TADs from IMR90 fibroblast. Fig S2. LocalZscore" as a function of shifted intervals. As soon as we displace the regions away from their original positions, TADs vs. isochores z-scores (A) drop considerably in a similar fashion to the TADs-hESC vs TADs-IMR90 z-scores (B). Fig S3. CTCF binding sites density. Density of 23,751 CTCF binding sites (from Li et al, 2013) in the five isochore families of the human genome. Fig S4. Isochores and chromatin subcompartments. The figure shows an average match between isochores/isochore blocks and chromatin subcompartments. Blue/Black triangles are examples of good/bad matches. An additional subcompartment called B4 was detected only in human chr19 (Rao et al, 2014), a chromosome composed (see Fig S42) mainly of GC-rich isochores and short/weak anchors to the lamina; its GC-poor chromatin configuration can be viewed as a particular case of compartment B3. Figs S5-S23. Chromatin interactions of mouse chromosomes. Chromatin interactions (from Vietri Rudan et al, 2013) are compared at a resolution of 250 Kb with the corresponding isochore profiles (see legend of Fig 1A for further information). H3 isochores are known to be very scarce in the mouse compared to the human genome; this makes the mouse genomic landscape less rugged and easier to be visually explored (this is so even for small chromosomes; see for instance chr18). A common emerging property is that intrachromosomal interactions have similar features across all mouse chromosomes; GC-poor isochores (L1 and L2 families) interact with each other, whereas GC-rich isochore interact only weakly (see for example chr8). Figs S24-S45. Chromatin interactions of human chromosomes. Chromatin interactions (f [file pone.0168023.s002.pdf]
